# SCW: building the whole-genome 3D structures based on extremely sparse single-cell Hi-C data

**DOI:** 10.1186/s12859-026-06421-3

**Published:** 2026-03-17

**Authors:** Hao Zhu, Tong Liu, Bishal Shrestha, Zheng Wang

**Affiliations:** 1 Department of Computer Science, Florida Memorial University, 15800 NW 42 Ave, Miami Gardens, FL 33504 USA; 2 Department of Computer Science, University of Miami, 330M Ungar Building, 1365 Memorial Drive, Coral Gables, FL 33124-4245 USA

**Keywords:** 3D whole-genome reconstruction, Extremely sparse single-cell Hi-C, Computational genomics

## Abstract

**Background:**

The study of three-dimensional (3D) genome structures at the single-cell level is crucial for understanding cell-to-cell variability. However, it is challenging to reconstruct the 3D structures of the whole genome based on single-cell Hi-C data because of the sparseness of the single-cell Hi-C data and the complexity of the problem.

**Results:**

To address this, we developed a new computational method, named SCW (single-cell whole-genome), to build the high-resolution 3D genome structures of the whole genome based on extremely sparse single-cell Hi-C data (either zeros or ones in the Hi-C matrix). We evaluated our reconstructed 3D genome structures on various types of cells, checked the fitness of the reconstructed 3D structures to the single-cell Hi-C data, cross-validated the reconstructed structures with FISH data, bulk Hi-C data, and gene expression data, and then compared SCW with the state-of-the-art tools Nuc_dynamics, Hickit, and Tensor-FLAMINGO. SCW achieved better robustness as Nuc_dynamics failed on extremely sparse Hi-C matrices that contained only zeros or ones. The Pearson Correlation between our reconstructed 3D structure and the FISH data can reach 0.63, which is higher than the structures built by Hickit. SCW can also build better structures compared to Tensor-FLAMINGO based on multiple evaluations, particularly with the 20 Kbp resolution. Both the intra- and inter-chromosomal contact patterns are maintained in our reconstructed 3D structures, which also match the findings from single-cell gene expression data.

**Conclusions:**

SCW enables high-precision whole-genome 3D reconstruction from extremely sparse single-cell Hi-C data. SCW outperforms existing tools in structural accuracy and robustly maintains intra/inter-chromosomal contacts. Its versatility is validated across diverse cell types.

**Supplementary Information:**

The online version contains supplementary material available at 10.1186/s12859-026-06421-3.

## Background

The chromosomal structure is critical for understanding how cells encode and regulate genetic information [1]. The three-dimensional (3D) genomic structures can influence their accessibility to the cellular machinery responsible for reading and interpreting the genetic code. In recent years, various techniques have been developed to study chromosomal structures at different levels of resolution, such as Hi-C [2].

Hi-C is a powerful technique that can capture genome-wide chromatin interactions across a cell population. It works by cross-linking DNA, cutting fragment pairs that are physically in proximity in 3D space with the restriction enzyme, followed by ligation and sequencing. The resulting paired-end reads can be used to generate a map of the spatial relationships between different genomic regions.

While bulk Hi-C has provided valuable average insights into the genome-wide chromatin interaction maps, more recent advances in single-cell Hi-C [3] have allowed for the studies of chromosomal structures at the single-cell level. The single-cell Hi-C techniques provided a new way to explore cell-to-cell heterogeneity and relationships between 3D structures and external factors, such as the cellular environment [4] and genetic perturbations [5].

The studies of single-cell genome-wide DNA structures have the potential to shed light on a wide range of biological processes, including gene regulation [6] and the organization of the genome within the cell nucleus [7]. As such, it represents an exciting and rapidly growing field that has the potential to enrich our understanding of the molecular basis of cellular function [3, 7, 8].

There are a lot of tools that can be used to build 3D whole-genome structures based on both bulk and single-cell Hi-C data. Chrom3D [9] is software for 3D genome modeling based on the inclusion of positional input constraints from chromosomal interactions (Hi-C data) and nuclear Lamin-chromatin associations (Lamin ChIP-seq data). An application of Chrom3D has been demonstrated in the study of disease mechanisms, particularly using patient-specific positional constraints from FPLD2 cases with an LMNA mutant that alters its genomic associations.

Chrom3D is also capable of filtering and normalizing single-cell Hi-C data, subsequently generating 3D structures based on this data. However, its application has been limited to single-cell topologically-associated domains (TADs), which are regions of the genome with higher contact frequencies. While effective within TADs, Chrom3D cannot handle data across entire chromosomes, highlighting its limitations in comprehensive single-cell Hi-C data analysis.

In the study of [10], the researchers used a computational method called FLAMINGO to reconstruct the bulk and single-cell 3D structure of the human genome, which is the complete set of genetic instructions found within the human body’s cells. FLAMINGO uses statistical models to account for the noise in the Hi-C data and infer the true underlying structure of the genome. While FLAMINGO shows great performance in imputing missing data for bulk Hi-C datasets, the researchers stated that highly sparse single-cell Hi-C datasets still require further algorithmic improvements to accurately characterize the detailed structural variations across individual cells.

Tensor-FLAMINGO [11] is a tool that can take the single-cell Hi-C data of tens to hundreds of cells as input and infer 3D structures of all the cells through complex low-rank tensor completion. Its core technique involves low-rank tensor completion, which aims to impute missing data in sparse single-cell Hi-C maps by borrowing information across cells while preserving their unique structural variations. While this design optimally utilizes data from multiple cells (the single-cell Hi-C data of multiple cells as input), the tool can also be executed with input from only a single cell (the single-cell Hi-C data of only one cell as input). Unlike SCW, Tensor-FLAMINGO can only generate the 3D structures for individual chromosomes, not the 3D structure of the whole genome.

Nuc_dynamics [6] is a whole-genome single-cell structural modeling tool using a simulated annealing particle dynamics protocol [12]. For Nuc_dynamics, the quality of the output structure depends on having a sufficient number of contacts and a reasonable proportion of inter-chromosomal contacts. This tool is not effectively designed to work with extremely sparse single-cell Hi-C data containing only zeros and ones, but in general needs contact values in the range of 0–5.

Hickit is a software kit that can impute missing values in single-cell Hi-C data and infer 3D genome structures of single cells, and the computer program used in Hickit for reconstructing 3D genome structures is an upgraded version of Nuc_dynamics. Hickit has been used to build the 3D genome structures of single cells in high-impact work, including Dip-C [13] and HiRES [14]. However, Hickit requires more complex parameter inputs for 3D structure reconstruction, resulting in highly specific but less generalizable modeling performance. This parameter-intensive approach limits its applicability across diverse biological contexts compared to our SCW framework.

Here, we introduce SCW for building the whole-genome 3D structures based on extremely sparse single-cell Hi-C data that only contains zeros and ones. Despite requiring only minimal input: single-cell Hi-C contact pairs formatted as chromosomal position pairs (chrA_pos1, chrB_pos2), SCW achieves high-precision 3D whole-genome reconstruction. The robustness of SCW is further exemplified by its exceptional versatility across diverse cell types.

## Methods

### Overview

We set the Hi-C value to one for all the bead pairs that have ≥ 1 raw single-cell Hi-C contacts, and zero for all other bead pairs. In other words, our system is designed to work with the extremely sparse single-cell Hi-C data that contains only zeros and ones. The bead pairs having > 1 Hi-C contacts are only 0.04%, 0.0003%, and 0.00003% of all bead pairs in the Hi-C contact matrices of 500 Kbp, 50 Kbp, and 20 Kbp resolutions of the NXT896 cell (Supplementary Fig. S1), showing that flattening the Hi-C contacts into zero and one will not lose core information. These contact matrices that contain only zeros and ones (dominated by zeros) are input into SCW to build 3D genome structures. Notice that our previous tool SCL [15] can only build the 3D structure of individual chromosomes, but SCW is able to build the 3D structures of the whole genome.

The single-cell Hi-C data that are composed of only 0s and 1s are imputed in SCW. One imputation approach that we applied and benchmarked is based on the 2D Gaussian function introduced in our SCL [15], and the other imputation approach is a newly developed approach based on graph networks, which will be elaborated in a later section.

The large number of beads being considered for optimizing the loss function makes the algorithm have a heavy computational workload. To improve the efficiency and speed of the program, we used C++ to implement SCW and allow the calculations of the loss functions to be performed in parallel using multiple threads. This allows the whole problem to be divided into smaller, independent tasks that can be solved simultaneously, significantly reducing the overall time required to complete the task in sequential processing.

### 3D structural modeling and the loss function

Our method represents each chromosome as a chain of consecutive DNA beads, with each bead having a size equal to the resolution value (for example, 50 Kbp) and being put in a 3D cubic lattice with unit size. A chain of beads covering all chromosomes, following the order of chromosomes starting from chromosome 1 to the last chromosome (X or Y, depending on the cell), is formed. Notice that this sequential order of chromosomes is only for the initial random structure and will be altered during the following steps.

A random 3D structure of this chain of beads is put in a cubic lattice with a volume of $V = \left( {5l} \right)^{3}$, where *l* is the number of beads of the whole genome. Metropolis–Hastings sampling was then performed to find the 3D genome structure that best fits the loss functions.

The core formulation of the SCW loss function follows our prior paper SCL [15] and are summarized below. The SCW software package also includes a step of imputation using a 2D Gaussian function and another step of simulated annealing with a specific cooling schedule described in [15]. The initial temperature is set to $T_{0} = 10$, and then decreases according to $T_{c} = T_{0} \times 0.9^{c}$, where $c$ represents the number of temperature reductions performed so far, and $T_{c}$ is the current temperature. At each temperature, the algorithm continues until the system reaches equilibrium, defined as either an average of 10 accepted moves per DNA bead or a maximum of 100 attempts per bead has been achieved. Once either condition is met, the temperature is decreased, and the simulation continues at the new temperature.

Specifically, a theta matrix is generated to indicate the estimated probability of the existence of a single-cell Hi-C contact between each pair of beads. When there is a single-cell Hi-C contact present between a pair of beads, their theta value is set to one. For bead pairs that do not have a single-cell Hi-C contact, the closer they are to other bead pairs that do have a single-cell Hi-C contact, the higher their theta value will be assigned, which was modeled by a 2D Gaussian function [15].

Specifically, the loss function consists of three mathematical equations to handle three different situations for bead pairs:

Those with an imputed value of one. These bead pairs either have a > 1 single-cell Hi-C contact in the original unimputed contact matrices or were imputed to have a Hi-C contact. Therefore, the mathematical formula for these bead pairs is the most stringent, meaning the loss function will give the largest penalty if these interaction relationships are not maintained in the reconstructed 3D structure.

Those with an imputed value between 0.7 and 1. These bead pairs have an imputed contact probability between 0.7 and 1 and do not have a contact in the original single-cell Hi-C data. Therefore, we are not as confident in reinforcing spatial proximity for these bead pairs as in the previous case, so the mathematical equation for this case gives a lesser penalty if the in-contact relationships are not maintained in the reconstructed 3D structures. The value of 0.7 is determined based on tests of different numbers accordingly to the evaluation of the 3D structures generated based on FISH data, results shown in Supplementary Fig. S2. It can be found that thresholds 0.7 and 0.5 result in similar correlations to the FISH data, so we arbitrarily chose 0.7 as the threshold.

Those with an imputed value less than 0.7. We are least confident about these bead pairs having Hi-C contacts since their predicted probabilities of having edges are < 0.7. Therefore, the equation for this case is the loosest, allowing maximum fluctuations for their Euclidean distances in the reconstructed 3D structures.

The parameters used in SCW are the same as the ones used in SCL since these parameters have been benchmarked in our previous research for constructing 3D genome structures for individual chromosomes. Moreover, based on our evaluations in this research, these parameters work well for building whole-genome 3D structures.

### Using a graph autoencoder to impute the zero single-cell Hi-C contact data

We also developed and benchmarked a new imputation approach that utilized the edge prediction mechanism of a graph autoencoder to generate the imputed single-cell Hi-C data. The graph autoencoder (GAE) [16] operates on an undirected and unweighted graph $G = \left( {V,E} \right)$ with the number of nodes being $N = \left| V \right|$. It utilizes two matrices of $G$: an adjacency matrix $A$ and a degree matrix $D$. The adjacency matrix represents the connections between nodes in the graph $G$, with the diagonal elements set to 1 to indicate self-loops. The degree matrix describes the degree of all the nodes in the graph $G$.

GAE introduces stochastic latent variables $Z_{i}$ for each node, which are consolidated into an $N \times F$ matrix $Z$, where $N$ is the number of nodes and $F$ is the embedding size. We used 128 as the embedding size and tested the learning rates from 0.1, 0.001, 0.0001 to find the parameters that lead to the best results, which are AUG and AP both > 0.98. The features of the individual nodes are represented as an $N \times D$ matrix $X$. In this work, we treat a genome-wide single-cell Hi-C contact matrix as an input graph to GAE and utilize the frequency of single-cell Hi-C interactions as the node features in the graph. For any bead pairs with a raw Hi-C contact number > 1, we set one to their Hi-C contact matrices, and all other numbers in the matrices are set to zeros. These matrices were input into the autoencoder.

The GAE produces a reconstructed adjacency matrix $\hat{A}$, represented as: $\hat{A} = \sigma \left( {ZZ_{T} } \right),$ in which $Z = GCN\left( {X,A} \right)$. The $\sigma \left( . \right)$ is the logistic sigmoid function. The $GCN\left( . \right)$ is a two-layer graph convolutional network, defined as $GCN\left( {X,A} \right) = \hat{A}ReLU\left( {\hat{A}XW_{0} } \right)W_{1}$, with $W_{i}$ being the weight matrices, $ReLU\left( . \right) = {\mathrm{max}}\left( {0, .} \right)$, and $\hat{A} = D^{{\left( { - 1/2} \right)}} AD^{{\left( { - 1/2} \right)}}$. This reconstructed adjacency matrix can be considered as a reconstructed graph that fits the patterns of the input graph, but with newly predicted edges added along with their probabilities. In our problem, when two beads do not have an edge, it is equivalent to that these two beads have zero single-cell Hi-C contact, and when the two beads are predicted with a certain probability to have an edge, it has the same effect of replacing a zero in the single-cell Hi-C matrix with a non-zero value or imputing the zero-dominated single-cell Hi-C contact matrices.

For the 1 Mbp diploid 3D structures comparative evaluation that we will mention in the Results section, the GAE-imputed matrices were benchmarked as an approach for constructing the theta matrix and compared with the 2D Gaussian method. For the high-resolution structures like 50 Kbp and 20 Kbp, GAE-imputed matrices were not available due to the limitation of the memory size in our computational server.

### Revised loss function with inter- and intra-chromosomal balancing

To address the imbalance between the number of inter- and intra-chromosomal interactions in contact frequency, we introduce a balancing coefficient $C_{{{\mathrm{ratio}}}}$. This coefficient is derived from the ratio of genome-wide intra-chromosomal contact counts (${\mathrm{count}}_{1}$) to genome-wide inter-chromosomal contact counts (${\mathrm{count}}_{2}$):

$C_{{{\mathrm{ratio}}}} = \frac{1}{{\left\lfloor {{\mathrm{count}}_{1} /{\mathrm{count}}_{2} } \right\rfloor + 1}}$, where $\left\lfloor \cdot \right\rfloor$ denotes the floor function. When calculating a loss value, intra-chromosomal loss terms are weighted by $C_{{{\mathrm{ratio}}}}$, while inter-chromosomal losses are weighted by $1 - C_{{{\mathrm{ratio}}}}$. This adjustment ensures inter-chromosomal contact patterns are maintained in the reconstructed 3D genome structure, which otherwise would be dominated by intra-chromosomal contacts due to the large amount of intra-chromosomal contacts.

### Two-step structural generation framework from coarse-grained to fine-grained 3D structures

We developed a two-step modeling approach, generating a low-resolution structure first and expanding it to a high-resolution structure, and compared its performance with one-step approach, directly generating high-resolution structures.

In the two-step approach, a coarse-grained 3D chromatin structure is generated at a lower resolution, for example, 10 Mbp resolution, using simulated annealing. This initial model then undergoes the following resolution scaling operation to prepare for the second refinement step: (1) The coordinates of the low-resolution beads are linearly scaled by a factor of $0.12 \times \left( {{\mathrm{low}}\_{\mathrm{resolution}}/{\mathrm{high}}\_{\mathrm{resolution}}} \right)$, followed by (2) interpolating $\left( {{\mathrm{low}}\_{\mathrm{resolution}}/{\mathrm{high}}\_{\mathrm{resolution}} - 1} \right)$ equidistant beads between each pair of adjacent beads. The value 0.12 was chosen based on tests of different values and their fitness with FISH data, results shown in Supplementary Fig. S3. For example, when targeting 1 Mbp resolution, 9 new beads are inserted between each original bead pair if started at 10 Mbp resolution. The scaled and interpolated structure then serves as the initial structure for the second simulated annealing stage, which produces a high-resolution model. When the target resolution is at 0.5 or 1 Mbp, both modeling stages employ identical cooling schedules to maintain consistency while the first stage captures global chromatin folding patterns, whereas the second stage optimizes local bead packing precision.

Notably, for extremely high-resolution whole-genome modeling shown in the Results section (50–20 Kbp resolution), where bead counts exceed 500000 which incur prohibitive computational costs, we adapted our two-stage framework with different cooling schedules. In the first stage, coarse-grained structures at 0.5 Mbp resolution were constructed with an initial temperature $T_{0} = 10$ and stopped when three consecutive rounds had no accepted moves. The cooling coefficient was set to 0.9, meaning $T_{n + 1} = T_{n} \times 0.9$. In the second stage, we modified the simulated annealing cooling schedule from the open-ended protocol to a defined temperature range: temperature starting at 10 and simulated annealing stopped when the temperature reached 0.1, and also reduced the cooling coefficient from 0.9 to 0.6 to make the temperature decrease faster.

### Multi-threads for calculating the values of the loss function

To reduce the computational time, we utilized the multi-threading package in C+ + . We divided the overall whole genome structure into equal parts and assigned each part to a thread. Each thread was then assigned to calculate the loss between the randomly selected bead and all other beads located in its assigned part. The results from all threads were then combined by one thread to calculate the overall loss before and after moving the randomly selected bead.

### Data

In this study, we used the single-cell Hi-C data from mouse and human cells. For mouse data, we analyzed three single cells (NXT896, NXT43, and NXT1037) from the dataset published by [17]. Human data included 13 GM12878 cells (IDs: 2, 3, 5, 6, 7, 9, 11–17) and 18 PBMC cells (IDs: 1–18) from the study [13]. We evaluated radial preferences by comparing our generated whole-genome structures with the published DNA FISH data of diploid cells in [13]. Those cross-validation analysis utilized chromosome-scale spatial measurements, where nuclear radial positioning was quantified through average Euclidean distances to the nuclear center of mass. We downloaded the distances between eight probe pairs located on chromosomes 3 and 11, which were detected by the 3D-FISH in the mES cells [18]. We then computed Pearson’s correlation between these experimental distances and corresponding distances derived from our inferred 3D structure. All data were analyzed at 500 Kbp resolution.

We also downloaded the whole-genome human bulk Hi-C data from [19] for performing evaluations based on the bulk Hi-C and simulated single-cell Hi-C data. The data for performing benchmark of the simultaneous single-cell Hi-C and single-cell gene expression were downloaded from [14] (shown in the Results section).

### Comparison with other existing modeling tools

We executed Nuc_dynamics [6], a 3D chromatin structure modeling tool, to benchmark against SCW using identical chromosomal inputs. The benchmarking utilized the single-cell Hi-C contact profiles from TH1 cell 1 (mouse type 1 T helper cells) in [6], formatted in the NCC data format (a description of NCC format can be found at https://github.com/tjs23/nuc_processing/blob/release 1.0/README.txt). For NCC format parsing, genomic coordinates were extracted from the first and third positional fields of each contact record to define the Hi-C contact values.

We also evaluated Tensor-FLAMINGO to assess its performance relative to SCW. Tensor-FLAMINGO is only capable of reconstructing 3D structures for individual chromosomes. As a result, the comparative benchmarking between Tensor-FLAMINGO and SCW was conducted exclusively at the intra-chromosomal level.

We further benchmarked SCW against Hickit [13], another computational tool for building the 3D chromosomal structures based on diploid single-cell Hi-C data. The results are presented in the Results section.

## Results

### Structural analysis based on the whole-genome diploid cells

**Fig. 1:**
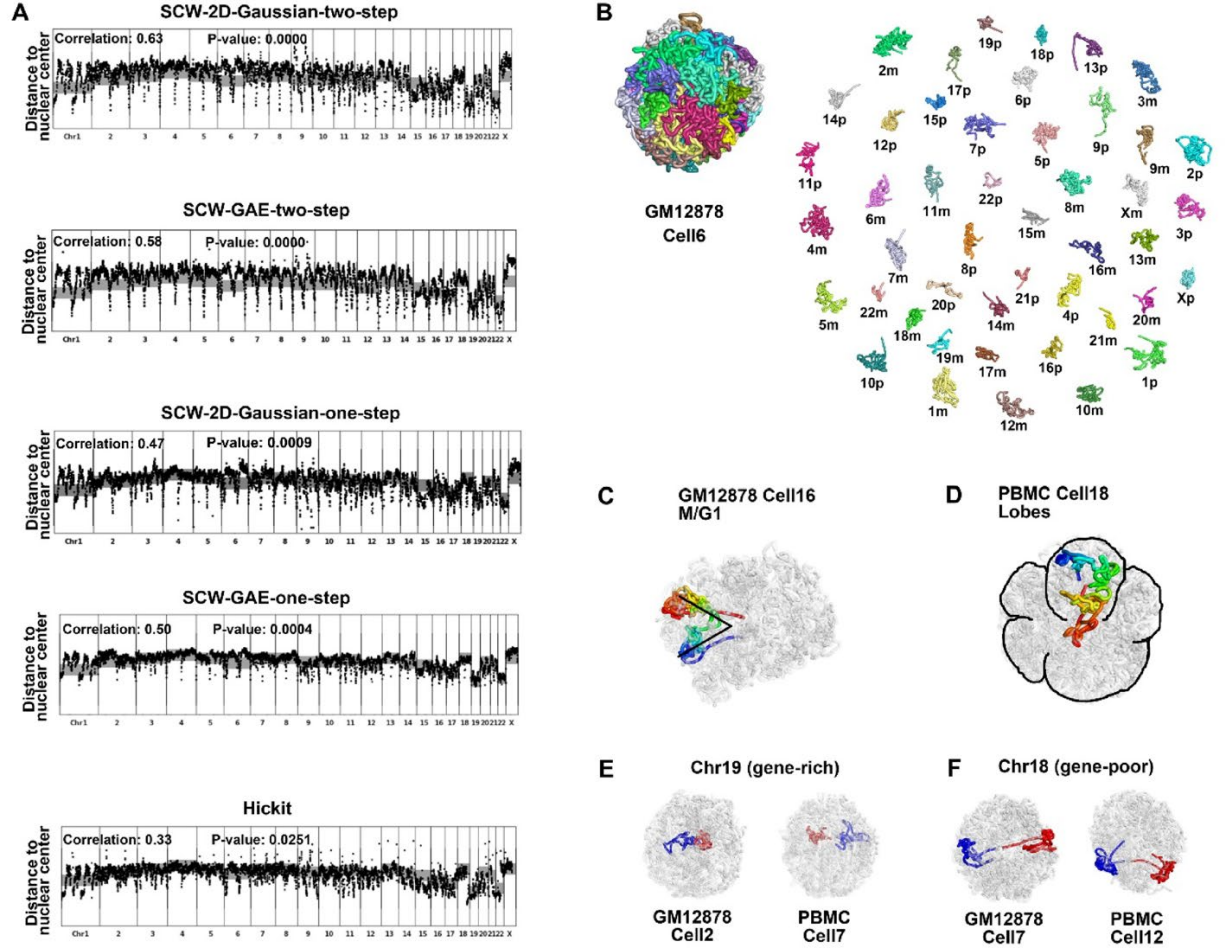
Structural analysis based on the whole-genome diploid cells. **A** Average distance to the nuclear center of every bead in the 1 Mbp whole-genome structures (black dots) generated by four SCW-variants and Hickit with published DNA FISH data (gray lines) on whole chromosomes. **B** The reconstructed 3D structures of the whole genome for GM12878 cell 6 at 1 Mbp resolution. **C** Observation of distinct nuclear morphology in 3D structure of SCW-inferred 1 Mbp GM12878 cell 16 that has recently undergone mitosis. **D** Observation of multiple nuclear lobes in 3D structure of SCW-inferred 1 Mbp PBMC cell 18. **E** SCW-inferred 3D structures of GM12878 cell 2 and PBMC cell 7 at 1 Mbp resolution. **F** SCW-inferred 3D structures of GM12878 cell 7 and PBMC cell 12 at 1 Mbp resolution

To systematically benchmark structural generation frameworks (two-step vs. one-step simulated annealing) and imputation strategies (GAE vs. 2D Gaussian), we implemented four SCW variants to reconstruct whole-genome 3D chromatin structures from 30 diploid single-cell Hi-C data, including 12 GM12878 cells (cells 2, 3, 5, 6, 7, 9, 11–15 and 17) and 18 PBMCs (cells 1–18). We quantitatively compared the average radial preferences of the SCW-inferred structures with the whole-genome DNA fluorescent in situ hybridization (FISH) data [20]. Figure 1A demonstrates radial positionings across the human genome (black dots) for all four SCW-derived and one Hickit-derived 1 Mbp resolution 3D structures, aligning with the whole-chromosome DNA FISH data (gray bars). We calculated the average radial distance of each chromosome to the nuclear center (mean across 30 single-cell structures) and derived Pearson correlation coefficients against whole-genome DNA FISH measurements. The two-step structural generation (10 Mbp structures first, and then 1 Mbp structures) with 2D Gaussian imputation demonstrated the best performance (Correlation = 0.63, *P* value = 0), establishing it as our standardized SCW framework for all subsequent analyses. In comparison, Hickit achieved a Pearson correlation coefficient of 0.33, with *P* value 0.0251.

We computed the Pearson’s correlation between the distances of eight probe pairs in the SCW-inferred (or Nuc_dynamics-inferred) 3D structure and the distances detected by 3D-FISH [18]. At 500 Kbp resolution, the correlation based on SCW-inferred structure is 0.57, whereas it is 0.53 based on the Nuc_dynamics-inferred structure. We generated the scatterplots related to the 8-pair FISH data and added them in the supplementary as Supplementary Fig. S4.

We reconstructed the overall 3D structures of the diploid human GM12878 cell 6 at 1 Mbp resolution for structural analysis (Fig. 1B). The top left panel displays the overall 3D structure, while the right panel shows the approximate spatial positioning of all chromosomes, in which”m” denotes the maternal chromosomes, and”p” denotes the paternal chromosomes.

During the M/G1-phase of a GM12878 cell, a well-established nuclear morphology is that the chromosomes retain their characteristic V-shaped configuration [13], a hallmark of recent mitosis. To show that our reconstructed 3D structure also maintains this finding, we present the SCW-reconstructed 3D structure of GM12878 cell 16 at 1 Mbp resolution (Fig. 1C). To enhance visibility, we colored the reconstructed 3D structure of paternal chromosome 2 and rendered the other structures in grey transparency. We observed that the paternal chromosome 2 structure displays a V-shaped opening, further emphasizing the preservation of the chromosomal architecture during the M/G1-phase of the cell cycle.

In several peripheral blood mononuclear cells (PBMCs), the presence of multiple nuclear lobes was observed, reminiscent of the partially segmented nuclei seen in low-density neutrophils and other blood cell types [13]. We reconstructed the 1 Mbp 3D structure of PBMC cell 18 (Fig. 1D). As we did for GM12878 cell 16 (Fig. 1C), we colored the paternal chromosome 5 with a rainbow color scheme and rendered the other chromosomes in grey transparency, demonstrating that the presence of multiple nuclear lobes is evident in the figure.

In Fig. 1A, both the DNA FISH data and the SCW-inferred 3D structures show that the gene-rich chromosome 19 tends to occupy the nuclear interior, whereas the gene-poor chromosome 18 tends to occupy the nuclear periphery. To further validate this, we generated whole-genome structures of GM12878 cell 2 and PBMC cell 7 at 1 Mbp resolution (Fig. 1E). The maternal and paternal chromosomes 19 are colored red and blue, respectively, while the other chromosomes are rendered in transparency. From this, we can observe the preference for gene-rich chromosome 19 located in the nuclear centers.

Similarly, we generated 3D structures of GM12878 cell 7 and PBMC cell 12 at 1 Mbp resolution (Fig. 1F), which shows that gene-poor chromosome 18 is in the nuclear periphery. These results match the findings from DNA FISH [20], which further validated the 3D whole-genome structures built by SCW.

### Evaluations of the 3D structures based on intra-chromosomal interactions

**Fig. 2:**
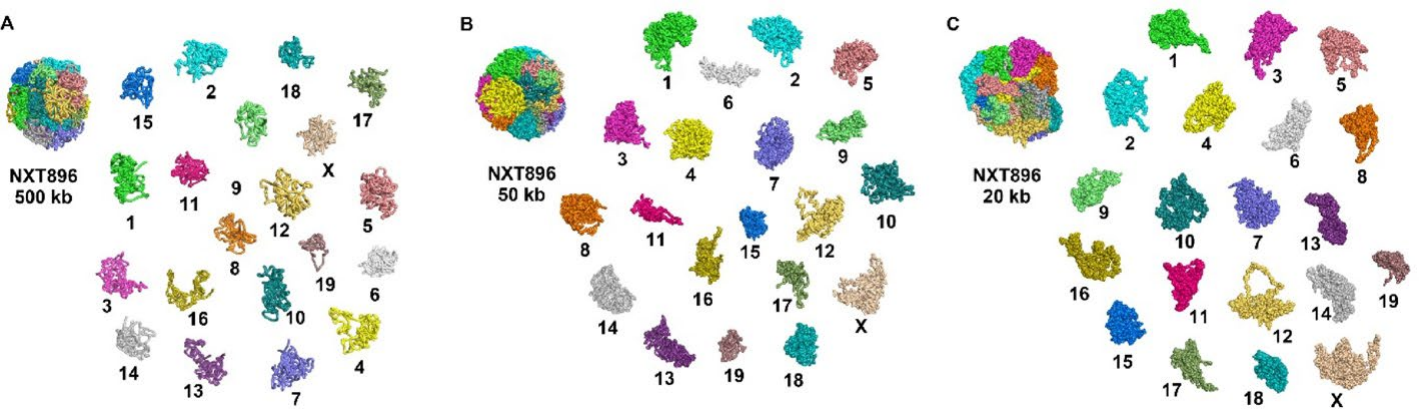
Whole-genome 3D structures and the separated chromosomes 3D structures for a mouse NXT896 cell. **A** 3D structures at 500 Kbp resolution. **B** 3D structures at 50 Kbp resolution. **C** 3D structures at 20 Kbp resolution

We used SCW to reconstruct the 3D structures of a haploid mouse NXT cell [17] and then evaluated the SCW 3D whole-genome structures at both 500 Kbp (two-step structural generation, 10 Mbp first and then 500 Kbp), 50 Kbp (two-step, 1 Mbp first and then 50 Kbp), and 20 Kbp (two-step, 1 Mbp first, and then 20 Kbp) resolutions using multiple criteria. In Fig. 2, we showed the SCW-inferred 3D whole-genome structures of the haploid mouse NXT896 cell at three resolutions, in which each chromosome was labeled in a unique color. The left panel displays the overall 3D structure of the whole genome, while the right panel illustrates the approximate spatial positioning of each chromosome. Figure 2A, B, and C display the structures at 500 Kbp, 50 Kbp, and 20 Kbp resolution, respectively.

**Fig. 3:**
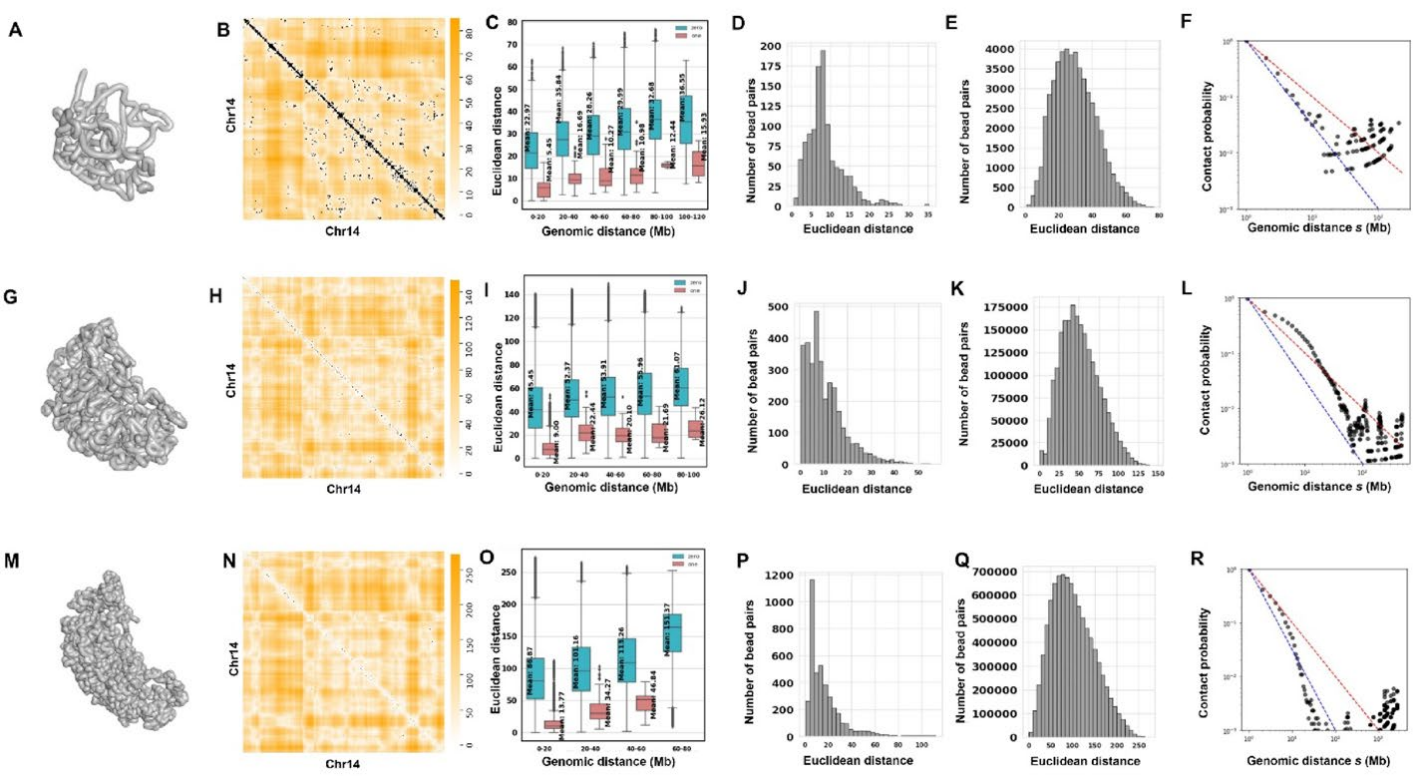
Comparison of SCW-inferred 3D structures with intra-chromosomal single-cell Hi-C data at 500 Kbp, 50 Kbp, and 20 Kbp resolutions. **A** The 3D chromosome structure for chromosome 14 of NXT896 cell at 500 Kbp resolution. **B** The overlay of the Euclidean distances parsed from the inferred 3D structures and the original single-cell Hi-C contact matrix for chromosome 14 of NXT896 cell at 500 Kbp resolution. **C** Group box plots showing the Euclidean distances parsed from the inferred 3D structures of chromosome 14 of NXT896 cell at 500 Kbp resolution. **D** The distribution of the Euclidean distances parsed from the inferred 3D structures of the bead pairs that have single-cell Hi-C contacts for chromosome 14 of NXT896 cell at 500 Kbp resolution. **E** The distribution of the Euclidean distances parsed from the inferred 3D structures of the bead pairs that do not have single-cell Hi-C contact for chromosome 14 of NXT896 cell at 500 Kbp resolution. **F** The relationship between contact probability and genomic distance s was parsed from the inferred 3D structure for chromosome 14 of NXT896 at 500 Kbp resolution. The two straight lines are: red line s^−1^, indicating a fractal globule, and s^−3/2^, indicating an ideal chain/equilibrium globule. **G**–**L** Similar to (**A**)–(**F**), but for the SCW-inferred 3D structure of NXT896 cell at 50 Kbp resolution. **M**–**R** Similar to (**A**)–(**F**), but for the SCW-inferred 3D structure of NXT896 cell at 20 Kbp resolution

To evaluate the reconstructed 3D structures, we first calculated the Euclidean distances between all bead pairs based on the 3D coordinates generated by our tool, resulting in a Euclidean distance matrix. A heatmap was then generated for this distance matrix, overlaid with the original single-cell Hi-C matrix. This serves as an internal consistency check to validate the inferred 3D structure. If the inferred 3D coordinates fit the single-cell Hi-C data, then the locations that have single-cell Hi-C interactions should overlap with smaller values of Euclidean distances. Figure 3A, G, and M show the 3D structures of chromosome 14 of mouse NXT896 inferred by SCW at 500 Kbp, 50 Kbp, and 20 Kbp resolutions, respectively. In Fig. 3B, H, and N, the heatmaps show the Euclidean distances parsed from the SCW-inferred chromosome 14 structures superimposed with the single-cell Hi-C contact heatmaps (black dots) at three resolutions.

We can observe that for the bead pairs that have single-cell Hi-C interactions, the distances between them and their adjacent beads in the 3D structure are significantly smaller, as represented by lighter colors. This suggests that the 3D structures generated by SCW match the corresponding single-cell Hi-C data. It also indicates that our tool effectively captures the properties of intra-chromosomal interactions in single-cell Hi-C data.

We drew box plots of the Euclidean distances derived from the SCW-inferred structures of chromosome 14 at three resolutions, as shown in Fig. 3C, I, and O. To facilitate analysis, we divided the Euclidean distances into two groups: one for the bead pairs that have single-cell Hi-C contacts (red) and another for the bead pairs that do not have any Hi-C contacts (cyan). Additionally, we considered different genomic distance ranges: 0–20, 20–40, 40–60, 60–80, 80–100, and 100–120 Mbp. By comparing the two groups, we can observe that the Euclidean distances for the bead pairs that have Hi-C contacts are significantly smaller than those for the bead pairs that do not have Hi-C contacts.

Figure 3D, E, J, K, P and Q show the number of bead pairs with different Euclidean distances parsed from the inferred 3D structures at three resolutions. Figure 3D, J, and P (in the same column) show the bead pairs that have single-cell Hi-C contacts at 500 Kbp, 50 Kbp, and 20 Kbp, respectively. Figure 3E, K, and Q show the bead pairs without single-cell Hi-C contacts. The bead pairs with single-cell Hi-C contacts have a peak value of Euclidean distance smaller than the cases without single-cell Hi-C contacts.

Figure 3F, L, and R display the relationship between contact probability and genomic distance s at 500 Kbp, 50 Kbp, and 20 Kbp, respectively. The two straight lines represent s^−1^ (red line, indicating a fractal globule) and s^−3/2^ (blue line, indicating an ideal chain/equilibrium globule). Specifically, the segment of chromosome 14 from 5 to 20 Mbp is in an equilibrium globule state, while the remaining sections are between equilibrium and fractal globule states. The concept of fractal globule chromosome packing was mentioned in the studies [8] and [2], based on bulk Hi-C data. Fractal globules are compact but organized structures that avoid knots and tangles, and equilibrium globules are usually considered thermodynamically equilibrated or energy-minimized; both are crucial for biological structures.

### Assessment of the 3D structures based on inter-chromosomal interactions

**Fig. 4:**
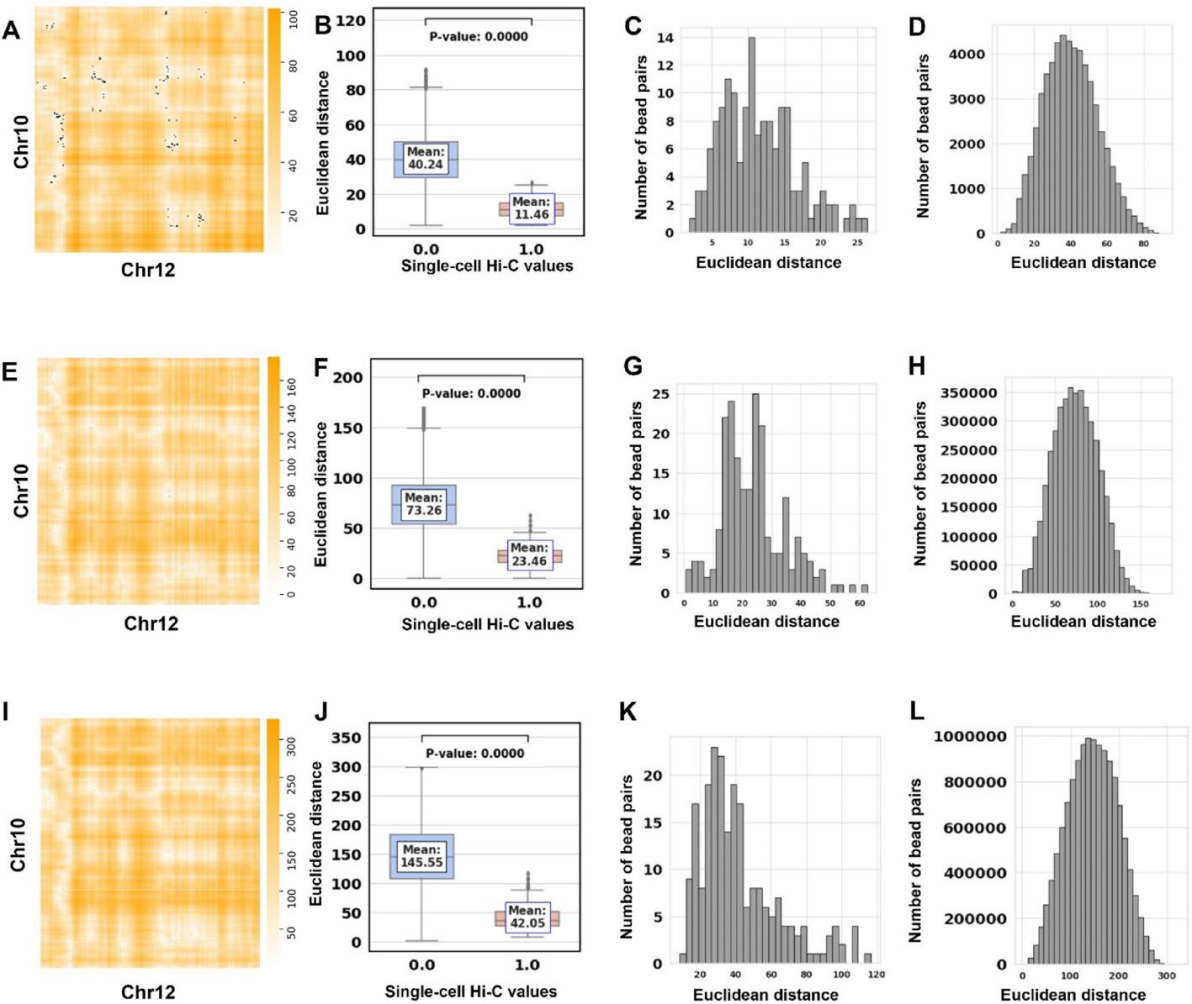
Comparison of SCW-inferred 3D structures with inter-chromosomal single-cell Hi-C data at 500 Kbp, 50 Kbp, and 20 Kbp resolution. **A** Overlay of the parsed Euclidean distances and single-cell Hi-C contacts between chromosomes 10 and 12 (NXT896 cell, 500 Kbp resolution). **B** Box plots showing the Euclidean distances parsed from the inferred 3D structure for the two chromosomes in (**A**). **C** The distribution of distances of bead pairs with single-cell Hi-C contacts in (**B**). **D** The distribution of distances of the bead pairs without single-cell Hi-C contacts in (**B**). **E**–**H** Similar to (**A**)–(**D**), but for the structure at 50 Kbp resolution. **I–L** Similar to (**A**)–(**D**), but for the structures at 20 Kbp resolution

We created an overlay that combines the single-cell Hi-C contact matrix (black dots) between chromosomes 10 and 12 (these two chromosomes were randomly picked) with the heatmap of Euclidean distances derived from bead pairs of the two chromosomes in mouse NXT896 at 500 Kbp resolution (Fig. 4A). We found that the number of inter-chromosomal single-cell Hi-C contacts is significantly lower than the number of intra-chromosomal single-cell Hi-C contacts. Furthermore, most bead pairs having single-cell Hi-C contacts are in spatial proximity to each other, as indicated by their smaller Euclidean distances.

We built box plots of the Euclidean distances between bead pairs from the two chromosomes, derived from the SCW-inferred 3D structure, see Fig. 4B. The bead pairs are divided into two groups based on the presence or absence of single-cell Hi-C contacts, with zero indicating the absence of contacts (cyan) and one indicating the presence (red). This also reveals that the Euclidean distances for the bead pairs that have Hi-C contacts are significantly smaller than those for the bead pairs that do not have Hi-C contacts. Figure 4C and D show the distributions of Euclidean distance for bead pairs with and without the single-cell Hi-C contacts based on the structure generated by SCW at 500 Kbp resolution. Figure 4D also shows the range of the Euclidean distances in the heatmap Fig. 4A.

Figure 4E–H illustrate the same analysis series but for the same cell NXT896 at 50 Kbp resolution. The plot in Fig. 4F demonstrates that the Euclidean distances between bead pairs with single-cell Hi-C contacts are significantly (*P* value = 0) smaller than those between bead pairs without having single-cell Hi-C contacts. This suggests that the inferred 3D structures also capture the properties of inter-chromosomal interactions. We also conducted similar analyses at 20 Kbp resolution and reached the same conclusions (Fig. 4I–L).

To show that our SCW works on multiple cells, we plotted the whole-genome 3D SCW-inferred structures for 30 cells based on their single-cell Hi-C data [13]. We also generated the superimposed heatmaps of intra-chromosomal and inter-chromosomal Hi-C and distance matrix derived from SCW-inferred structures. These figures can be found in Supplementary Figs. S5–S94. The superimposed heatmaps demonstrate that distances derived from the inferred structures exhibit high consistency with single-cell Hi-C contacts.

### Whole-genome 3D structure modeling for haploid cells

**Fig. 5:**
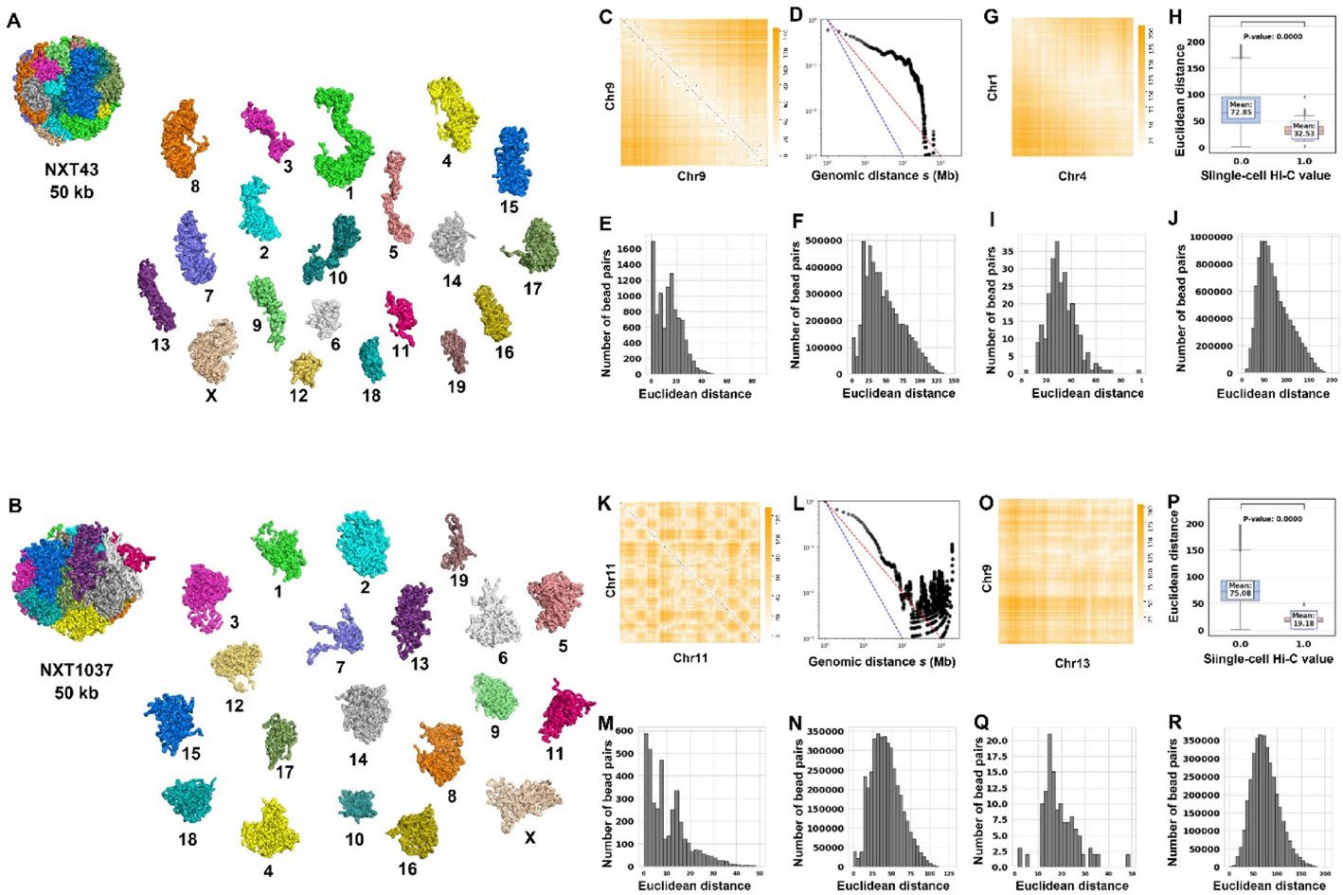
SCW whole-genome 3D structure modeling for haploid cells. **A** The reconstructed 3D structures of the whole genome for NXT43 cell at 50 Kbp resolution. **B** The reconstructed 3D structures of the whole genome for NXT1037 cell at 50 Kbp resolution. **C** The overlay of the Euclidean distances parsed from SCW-inferred 3D structures and the single-cell Hi-C contact matrix for chromosome 9 of the NXT43 cell at 50 Kbp resolution. **D** The relationship between contact probability and genomic distance s in (**C**). **E**–**F** The distributions of distances of bead pairs with or without single-cell Hi-C contacts in (**C**). **G** The overlay of the parsed Euclidean distances and single-cell Hi-C contacts between chromosomes 1 and 4 in NXT43 cell at 50 Kbp resolution. **H**–**J** The integrated box plots (**H**) and the separate distributions (**I**) and (**J**) of Euclidean distances of the bead pairs with and without the single-cell Hi-C contacts for chromosomes 1 and 4 in the NXT43 cell at 50 Kbp resolution. **K**–**N** Similar to (**C**)–(**F**), but for chromosome 11 of NXT1037 cell at 50 Kbp resolution. **O**–**R** Similar to (**G**)–(**J**), but for chromosomes 9 and 13 in the NXT1037 cell at 50 Kbp resolution

We tested SCW on modeling the whole-genome 3D structures for a mitosis cell (NXT43) and a late G1 cell (NXT1037) at 50 Kbp resolution in Fig. 5A and B. It can be found that the 3D structures were decondensed from a rod-like mitotic conformation to more spherical structures, which fits the findings in [17].

Figure 5C displays the overlapped heatmap of the Euclidean distance matrix parsed from the inferred structures with the single-cell Hi-C contact matrix (black dots) for chromosome 9 of the NXT43 cell. Figure 5D, E, and F show the relationship between contact probability and genomic distance, as well as the distributions of the Euclidean distances of the bead pairs with and without single-cell Hi-C contacts for the inferred 50 Kbp chromosome 9 structure.

Figure 5K–N show the same series of analyses for chromosome 11 of the NXT1037 cell. Similar conclusions can be drawn from the intra-chromosomal analysis for NXT43 and NXT1037 cells, indicating that the inferred intra-chromosomal structures agree with the corresponding single-cell Hi-C data.

Figure 5G shows the overlapped heatmap of the Euclidean distance matrix with the single-cell Hi-C data (Black dots) between chromosomes 1 and 4 in NXT43 cell at 50 Kbp resolution. Figure 5H–J present the integrated box plots and the separated distributions of Euclidean distance of the bead pairs with or without single-cell Hi-C contacts for these two chromosomes. These results show that the Euclidean distances between bead pairs with single-cell Hi-C contacts are significantly smaller (*P* value = 0) than those between bead pairs without single-cell Hi-C contacts.

Figure 5O–R present another set of analyses between chromesomes 9 and 13 in the NXT1037 cell at 50 Kbp resolution. We can draw a similar conclusion for NXT1037 as from the inter-chromosomal analyses of the NXT43 cell.

### Comparisons with other 3D genome structure reconstruction tools

We applied SCW to a mouse TH1 cell (cell 1 in [6]) to generate whole-genome structures and then compared the 3D structures with the ones generated by Nuc_dynamics [6]. The single-cell Hi-C data were converted to zeros and ones (binary) before input into SCW, and for Nuc_dynamics, converting to binary made the software completely fail to run. Therefore, we input the non-binary or the original single-cell Hi-C data (bead pairs may have 0 or > 1 contacts compared to 0 and 1 for SCW) when running Nuc_dynamics, which means the Nuc_dynamics was given more data than SCW.

**Fig. 6:**
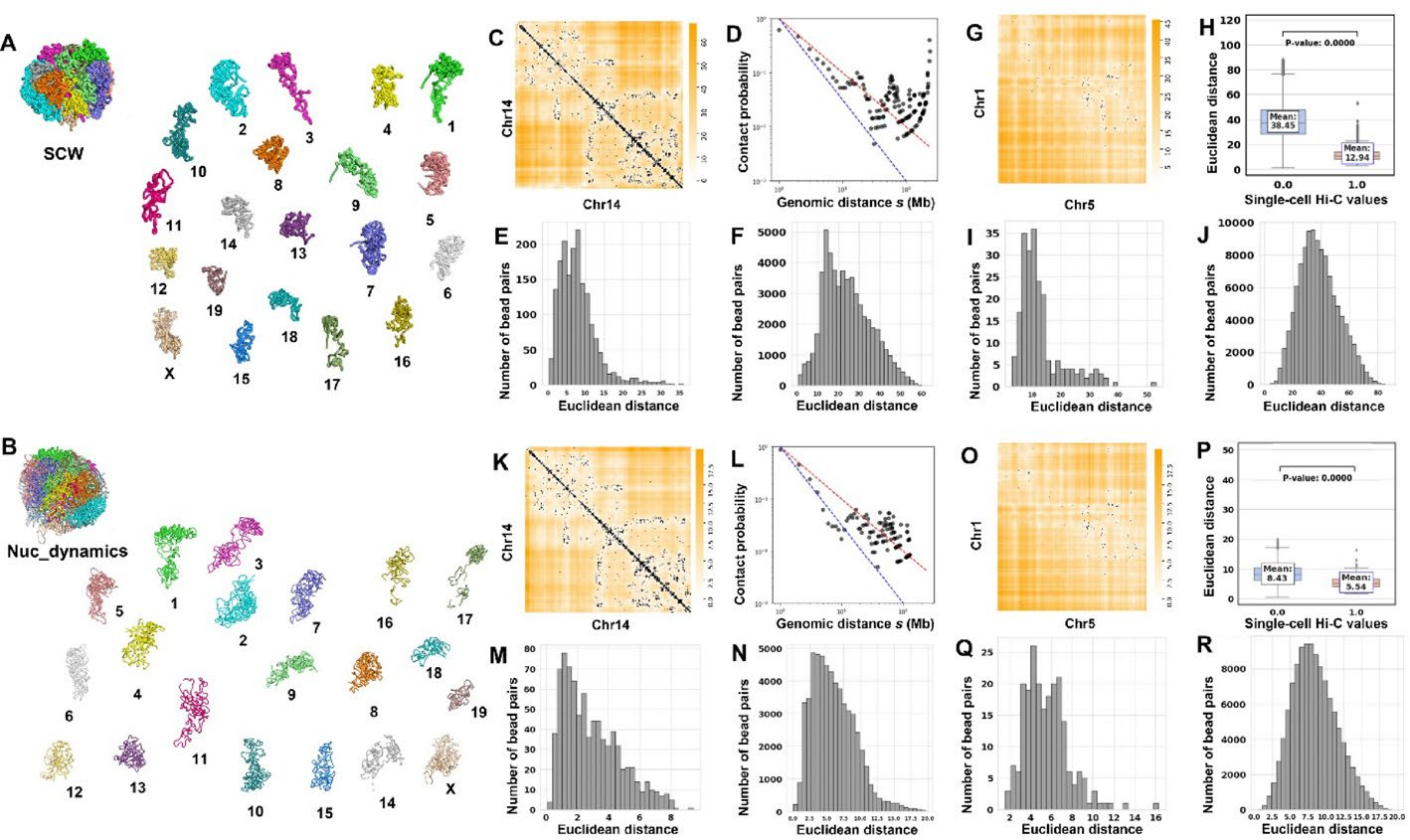
Structural analysis of the whole-genome haploid cell and comparisons with Nuc_dynamics. **A** The SCW-inferred 3D structures of the whole genome for mouse TH1 cell 1 at 500 Kbp resolution. **B** The Nuc_dynamics-inferred 3D structures of the whole genome for TH1 cell 1 at 500 Kbp resolution. **C** The overlay of Euclidean distances parsed from the SCW-inferred structures and the single-cell Hi-C contact matrix for chromosome 14 in mouse TH1 cell 1 at 500 Kbp resolution. **D** The relationship between contact probability and genomic distance s in (**C**). **E**–**F** The distributions of distances of bead pairs with and without single-cell Hi-C contacts in (**C**). **G** The overlay of the Euclidean distances parsed from the SCW-inferred structures and the single-cell Hi-C contacts between chromosomes 1 and 5 in mouse TH1 cell 1 at 500 Kbp resolution. **H**–**J**. The integrated distribution (**H**) and separate distributions (**I**) and (**J**) of Euclidean distances of bead pairs with and without single-cell Hi-C contacts for chromosome 1 and chromosome 5 in mouse TH1 cell 1 at 500 Kbp resolution. **K**–**R** Similar to (**C**)–(**J**), but for Nuc_dynamics-inferred mouse TH1 cell 1 structures at the same resolution

Figure 6A and B show SCW-generated (two-stage: 10 Mbp first, and then 500 Kbp) and Nuc_dynamics-generated structures of mouse TH1 cell 1 at 500 Kbp resolution. Figure 6C–F present the intra-chromosomal analyses for SCW-inferred structures for chromosome 14 in cell 1, whereas Fig. 6K–N show the same intra-chromosomal analyses for Nuc_dynamics-inferred structures for chromosome 14 in cell 1.

Figure 6G–J demonstrate the inter-chromosomal analyses for SCW-inferred structures based on chromosomes 11 and 9 in cell 1, whereas Fig. 6O–R present the same inter-chromosomal analyses for Nuc_dynamics-inferred structures on the same two chromosomes in cell 1. It can be found that both tools can model the structures by assigning smaller Euclidean distances for the bead pairs that have single-cell Hi-C contacts. However, the difference of SCW-inferred structures (38.45 vs. 12.94, which is more than three times) is much bigger than the Nuc_dynamics-inferred structures (8.43–5.54, which is less than twice). We added more intra-chromosomal comparisons between Hickit and SCW, and between Nuc_dynamics and SCW in Supplementary Figs. S95–S114.

**Fig. 7:**
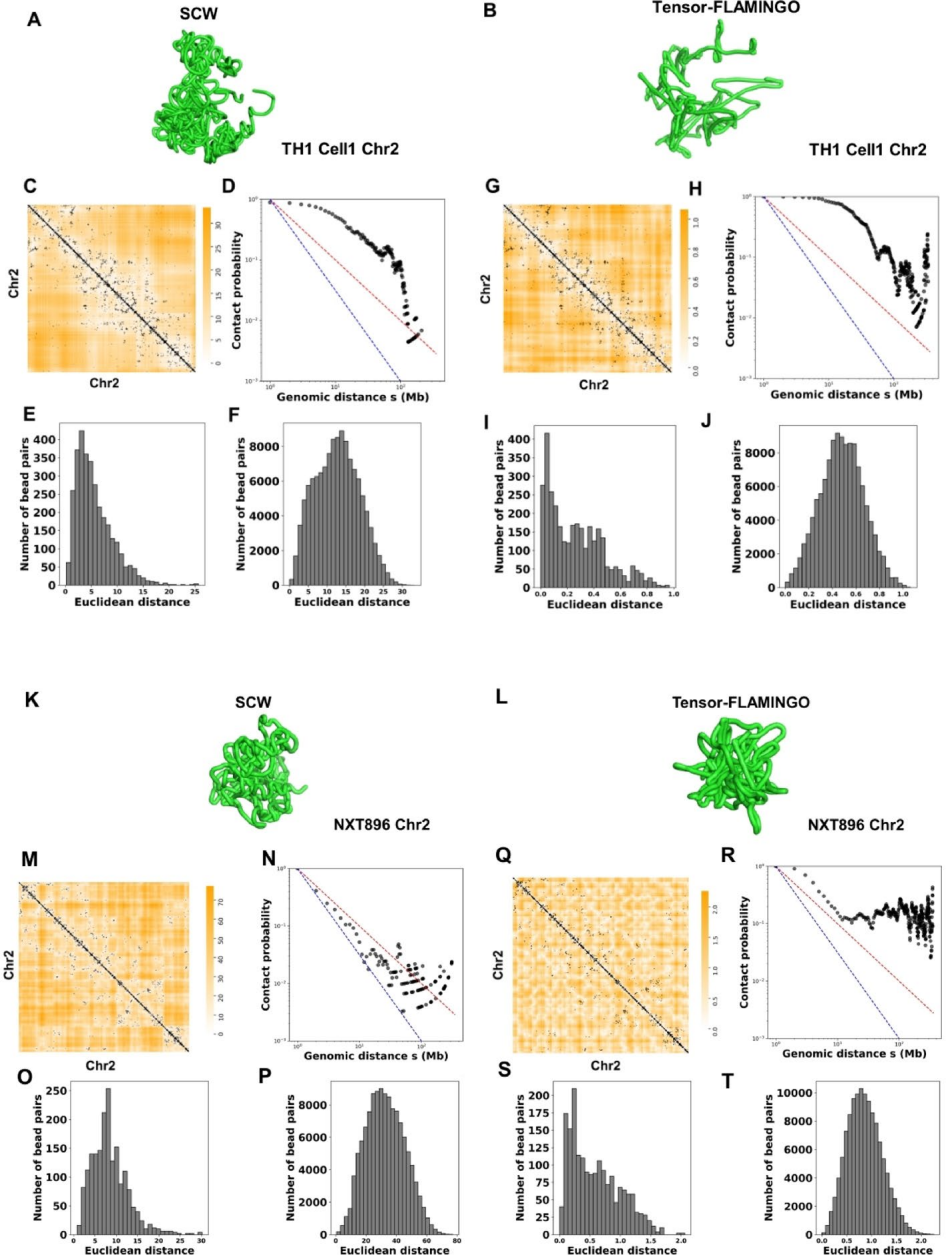
Structural analysis of the single chromosome and comparisons with Tensor-FLAMINGO. **A** The SCW-inferred 3D structure of chromosome 2 for mouse TH1 cell1 at 500 Kbp resolution. **B** The single-input Tensor-FLAMINGO-inferred 3D structure of the chromosome 2 for TH1 cell1 at 500 Kbp resolution. **C** The overlay of Euclidean distances parsed from the SCW-inferred structures and the single-cell Hi-C contact matrix for chromosome 2 in mouse TH1 cell1 at 500 Kbp resolution. **D** The relationship between contact probability and genomic distance s in (**C**). **E**–**F** The distributions of distances of bead pairs with and without single-cell Hi-C contacts in (**C**). **G**–**J** Similar to (**C**)–(**F**), but for single-input Tensor-FLAMINGO-inferred mouse TH1 cell1 chromosome 2 structure at the same resolution. **K** and **L** show the inferred 3D structures of chromosome 2 in NXT896 (500 Kbp resolution) generated by SCW and multi-input Tensor-FLAMINGO, respectively. The corresponding intra-chromosomal analyses are presented in **M**–**P** for SCW and **Q**–**T** for multi-input Tensor-FLAMINGO

We also evaluated and compared the 3D structures generated by Tensor-FLAMINGO. Since Tensor-FLAMINGO can impute missing data in sparse single-cell Hi-C inputs using either information from multiple cells or from one cell, we conducted two sets of evaluations. First, we only input the Hi-C data of TH1 cell1 into Tensor-FLAMINGO, obtained the 3D structure of chromosome 2, and compared it with the structure built by SCW. Notice that because SCW was designed to model the structure of the whole genome, but Tensor-FLAMINGO was designed to model the structure of individual chromosomes, we built the whole-genome structure of that cell using SCW, isolated the structure of chromosome 2, and compared only the structures of chromosome 2 for these two tools. Second, we randomly selected 100 NXT cells, input the Hi-C data of these 100 cells to Tensor-FLAMINGO, obtained the 3D structures of chromosome 2 s, and compared the structure of NXT896 with the SCW-inferred structure of chromosome 2. The results of this intra-chromosomal benchmark, which contrasts the performance of SCW and two input modes of Tensor-FLAMINGO (single-input and multi-input), are presented in Fig. 7.

Figure 7A and B show the inferred 3D structures of chromosome 2 in TH1 cell1 (500 Kbp resolution) generated by SCW and single-input Tensor-FLAMINGO, respectively. The corresponding intra-chromosomal analyses are presented in Fig. 7C–F for SCW and Fig. 7G–J for single-input Tensor-FLAMINGO.

Figure 7K and L show the inferred 3D structures of chromosome 2 in NXT896 (500 Kbp resolution) generated by SCW and multi-input Tensor-FLAMINGO, respectively. The corresponding intra-chromosomal analyses are presented in Fig. 7M–P for SCW and Fig. 7Q–T for multi-input Tensor-FLAMINGO.

The overlaid heatmaps in Fig. 7C and G show that the Euclidean distances from the SCW-inferred structure achieve a better fit to the single-cell Hi-C contact matrix than those from single-input Tensor-FLAMINGO. From Fig. 7H, it can be observed that the contact probability of the bead pairs for Tensor-FLAMINGO-inferred structure has a sharp increase as the genomic distances increase to a large value, which may not align with general knowledge, where the contact probability typically decreases with increasing genomic distance, as observed in Fig. 7D for SCW-inferred structure. This observation holds consistently across additional chromosomes tested, results shown in Supplementary Figs. S115–S124 (only for single-input Tensor-FLAMINGO).

When the Hi-C data of 100 cells were input into Tensor-FLAMINGO, it was found that the inferred structure did not lead to better agreement, compared to SCW, with the original Hi-C input data. This is demonstrated in Fig. 7M and Q, where the Euclidean distances from the SCW-inferred structure still achieve a closer fit to the single-cell Hi-C contact matrix than those from multi-input Tensor-FLAMINGO.

**Fig. 8:**
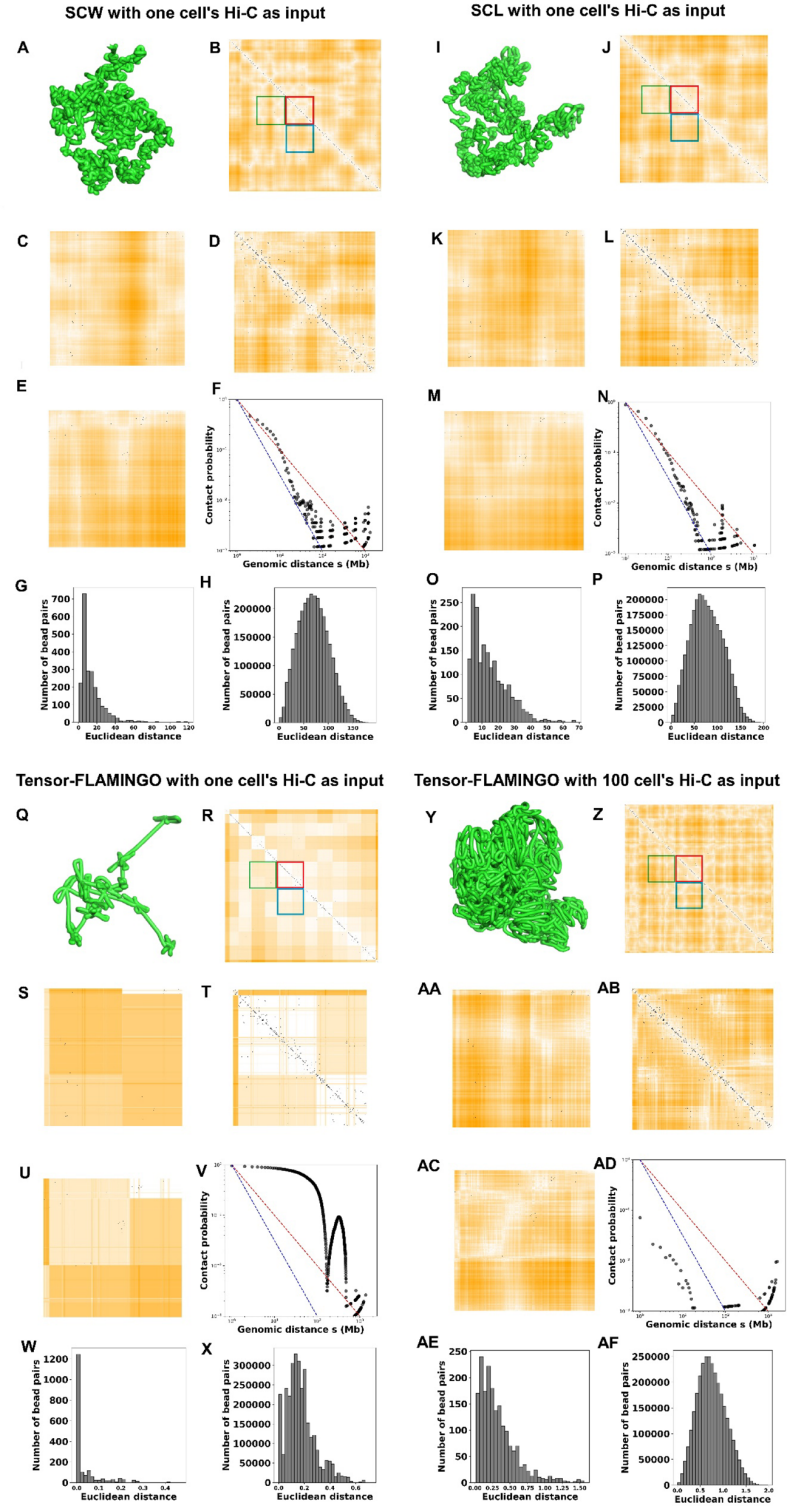
Single-cell 3D structure evaluation of chromosome 19 in the NXT896 cell line at 20 Kbp resolution. **A**–**H** Results from the SCW tool. **A** Inferred 3D structure. **B** Overlay heatmap of the Euclidean distances parsed from SCW-inferred structure and the single-cell Hi-C contact matrix. **C**–**E** Zoomed-in views of the green, red, and blue regions marked in (**B**), respectively. **F** The relationship between contact probability and genomic distance s derived from (**B**). **G**, **H** Distributions of Euclidean distances for bead pairs with and without single-cell Hi-C contacts, respectively. The same panel order (**A**–**H**) applies to the evaluations of: (**I**–**P**) the SCL tool, (**Q**–**X**) Tensor-FLAMINGO using one cell’s Hi-C as input, and (**Y**–**AF**) Tensor-FLAMINGO using 100 cells’ Hi-C contacts as input

The previous analysis for Tensor-FLAMINGO was based on a 500 Kbp resolution. Since Tensor-FLAMINGO is specifically designed to handle high-resolution, we also performed the analysis on the structures of chromosome 19 for NXT896 cell at 20 Kbp resolution. Because Tensor-FLAMINGO infers the structures for individual chromosomes rather than the whole genome, we further included a comparison with our SCL software, which also focuses on single-chromosome modeling. This can also show the differences between the structures of the same chromosome inferred by our previous software SCL and the software SCW, with the latter one being able to optimize the structures based on both intra- and inter-chromosomal contacts from a whole-genome perspective. The evaluation results are shown in Fig. 8. We also performed structural comparisons on chromosome 2 of the NXT896 cell at 20 Kbp resolution. However, due to the computationally prohibitive cost (out of memory) of using 100 cells’ Hi-C data as input, we only executed Tensor-FLAMINGO using one cell’s Hi-C data as input. These results are provided in Supplementary Figs. S125.

Based on the 20 Kbp resolution analysis, we reached conclusions consistent with those from the 500 Kbp resolution data. Overall, the models generated by SCW showed the closest agreement with the original Hi-C data, with inferred structures falling between a fractal globule and an equilibrium globule conformation.

**Fig. 9:**
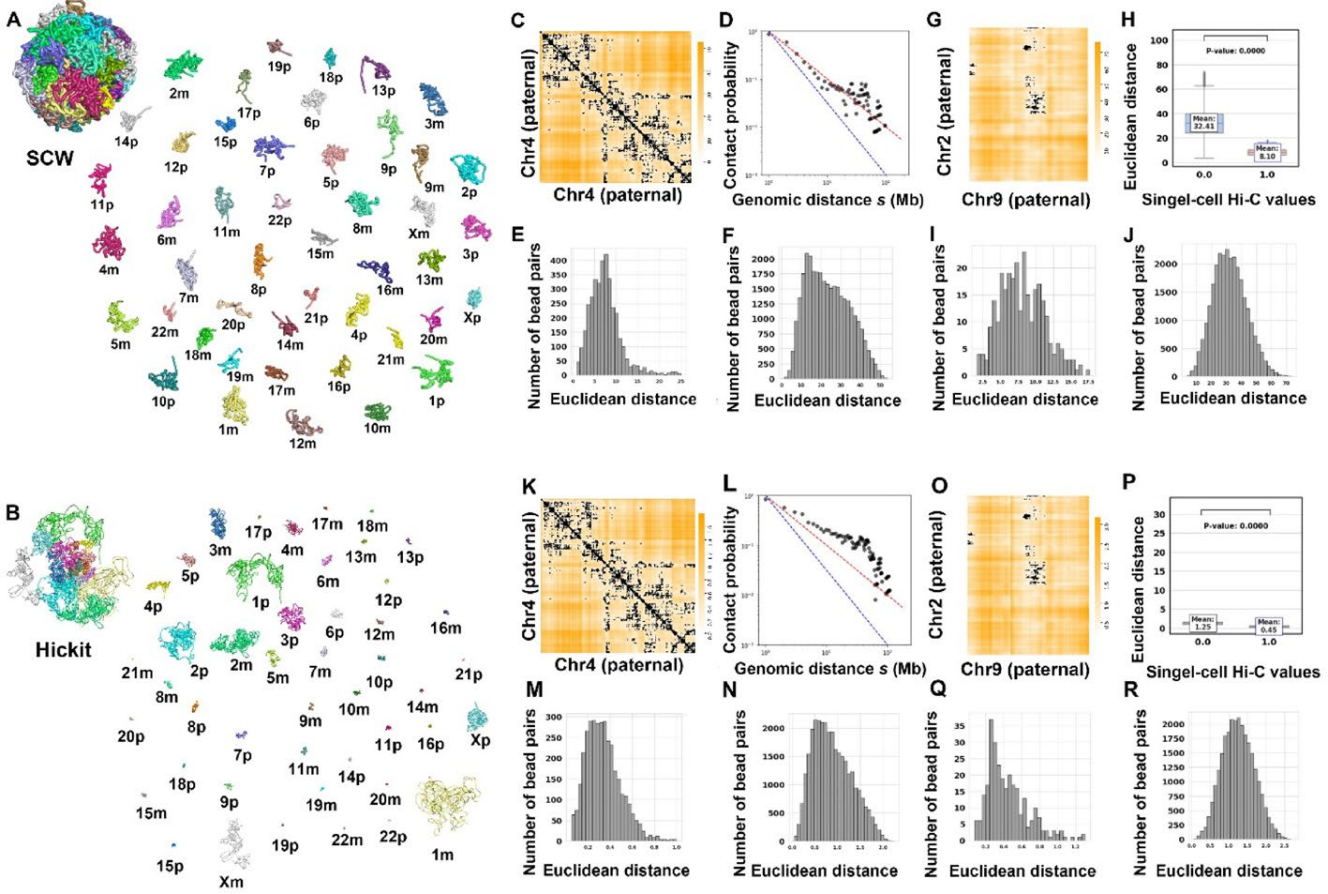
Structural analysis of the whole-genome diploid cell and comparisons with Hickit. **A** The SCW-inferred 3D structures of the whole genome for GSM12878 cell 6 at 1 Mbp resolution. **B** The Hickit-inferred 3D structures of the whole genome for GSM12878 cell 6 at 1 Mbp resolution. **C** The overlay of Euclidean distances parsed from the SCW-inferred structures and the single-cell Hi-C contact matrix for paternal chromosome 4 at 1 Mbp resolution. **D** The relationship between contact probability and genomic distance s in (**C**). **E**–**F** The distributions of distances of bead pairs with and without single-cell Hi-C contacts in (**C**). **G** The overlay of the Euclidean distances parsed from the SCW-inferred structures and the single-cell Hi-C contacts between paternal chromosome 2 and paternal chromosome 9 in human GSM12878 cell 6 at 1 Mbp resolution. **H**–**J** The integrated distribution (**H**) and separate distributions (**I**) and (**j**) of Euclidean distances of bead pairs with and without single-cell Hi-C contacts for paternal chromosome 2 and paternal chromosome 9 at 1 Mbp resolution. **K**–**R**. Similar to (**C**)–(**E**), but for Hickit-inferred structures

We applied SCW to a human diploid GM12878 cell (cell 6 in [13]) to generate whole-genome structures and then compared the 3D structures with the ones generated by Hickit [13]. Figure 9A and B show the SCW-generated (two-stage: 10 Mbp first, and then 1 Mbp) and Hickit-generated structures at 1 Mbp resolution. Figure 9C–F present the intra-chromosomal analyses for SCW-inferred structures for chromosome 4 (paternal), while Fig. 9K–N show the same intra-chromosomal analyses for Hickit-inferred structures for chromosome 4 (paternal).

In our contact probability calculations as shown in Fig. 9D and L, bead pairs were classified as interacting if their spatial distance fell below the peak of the genomic distance distribution. For SCW, this threshold was parameterized as the target distance, which was 8 in SCW, reflecting optimal distance, whereas for Nuc_dynamics and Hickit, the peak of the actual distances distribution (in Figs. 6M and 9M) defined the cutoff.

Figure 9G–J demonstrate the inter-chromosomal analyses for SCW-inferred structures based on chromosome 2 (paternal) and chromosome 9 (paternal), whereas Fig. 9O–R present the same inter-chromosomal analyses for Hickit-inferred structures on the same two chromosomes. It can be found that both tools can model the structures by assigning significantly smaller Euclidean distances for the bead pairs that have single-cell Hi-C. The 3D structures generated by our SCW better fit the fractal globule or equilibrium globule than the Hickit-inferred 3D structures.

### Benchmark on simulated single-cell Hi-C data and comparisons to the structures built on bulk Hi-C data

We first downloaded a bulk human whole-genome Hi-C data at 1 Mbp resolution [19] and generated 3D structures using a bulk Hi-C-based modeling tool, which was published in our previous paper [21]. Next, we down-sampled the bulk Hi-C data to 5% of the original contacts to simulate sparse single-cell Hi-C data and reconstructed 3D structures using SCW (SCW still used binary Hi-C contact matrices, that is, only 1s or 0s are included) and Nuc_dynamics (used all down-sampled Hi-C values). Specifically, we built 20 whole-genome 3D genome structures based on the bulk Hi-C data and 20 structures based on the simulated single-cell Hi-C data and conducted pairwise comparisons between the bulk-Hi-C-based structures and simulated-single-cell-Hi-C-based structures.

**Fig. 10:**
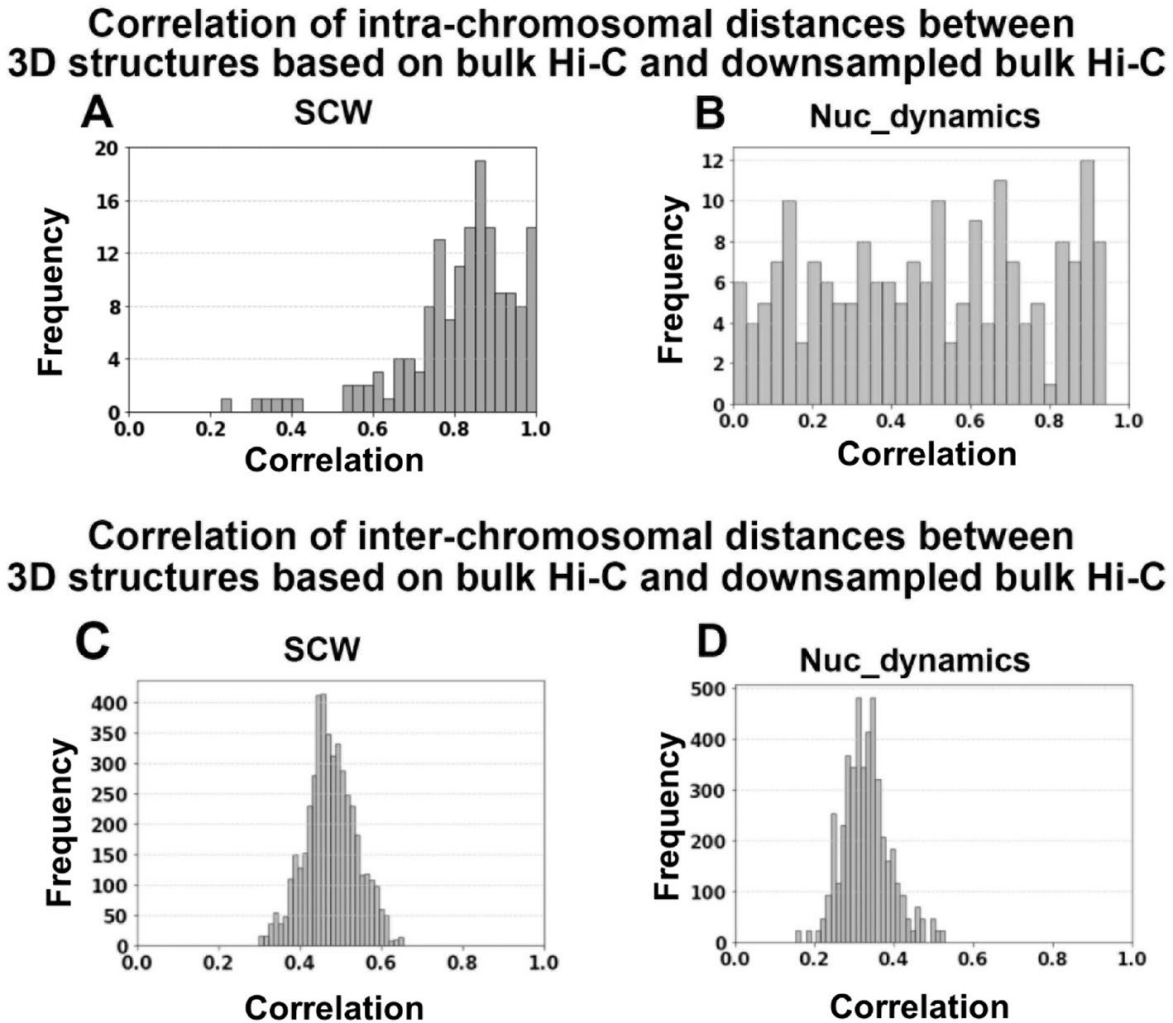
Comparison of 3D genome structures reconstructed based on bulk Hi-C and down-sampled bulk Hi-C data. **A** Histogram shows the correlation of intra-chromosomal Euclidean distance matrices between the 3D structures generated from bulk Hi-C and the SCW-inferred 3D structures based on the down-sampled Hi-C (5% of original contacts). **B** Similar to (**A**), but for Nuc_dynamics-inferred structures. **C** Histogram of the correlation between the inter-chromosomal distances derived from 3D structures based on bulk Hi-C and the structures generated by SCW based on down-sampled Hi-C data. **D** Similar to (**C**), but for Nuc_dynamics-inferred structures

In Fig. 10A, the histogram displays the distribution of the correlations of intra-chromosomal Euclidean distance matrices (distances of all bead pairs within the same chromosome) between 3D structures reconstructed from bulk Hi-C and those inferred by SCW from down-sampled bulk Hi-C (5% of original contacts or simulated single-cell Hi-C). Figure 10B shows a similar histogram comparing bulk Hi-C structures with the Nuc_dynamics-inferred structures based on simulated single-cell Hi-C data. These figures demonstrate that SCW-generated single-cell 3D structures align more closely with bulk Hi-C structures at the intra-chromosomal level compared to Nuc_dynamics.

In Fig. 10C, the histogram illustrates the distribution of the correlation for inter-chromosomal Euclidean distances (distances between all chromosome pairs) derived from the bulk and SCW-inferred structures. Figure 10D provides analogous comparisons between bulk Hi-C and Nuc_dynamics-inferred single-cell structures. Both panels confirm that SCW maintains stronger concordance with bulk Hi-C-based structures also at the inter-chromosomal scale.

### A/B compartment analysis and allelic-specific gene expression spatial mapping

**Fig. 11:**
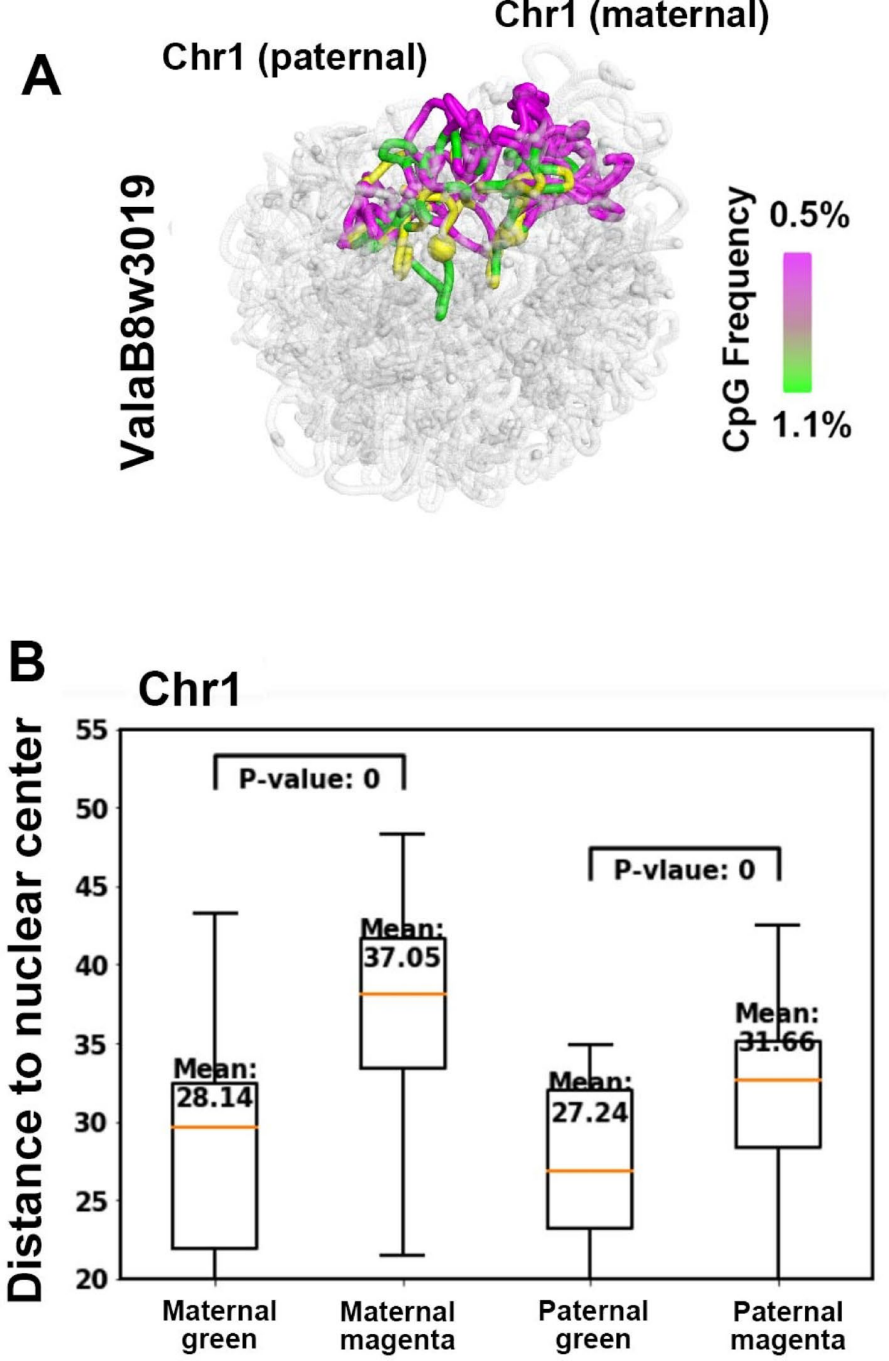
Mapping allelic-specific gene expression onto reconstructed 3D genome structures. **A** 1 Mbp 3D SCW-inferred structures of paternal and maternal chromosome 1 for cell ValaB8w3019, with allele-specific gene expression mapped as yellow spheres. The size of each sphere is proportional to the expression level detected by HiRES. CpG frequency is color-coded on the chromosome structures, with green indicating high CpG content and magenta indicating low CpG content. **B** Boxplots showing the average distances from the nuclear center for 20 SCW-reconstructed 3D structures. Regions with higher CpG content (green) are closer to the nuclear center, while regions with lower CpG content (magenta) are closer to the nuclear periphery. Active gene expression regions are observed to localize closer to the nuclear center, consistent with the spatial organization of chromatin and gene activity found in HiRES paper [13]

We combined the gene expression data with the 3D genome structure to further evaluate the reliability of the reconstructed genome conformation. In Fig. 11A, the 3D structures of the paternal and maternal chromosome 1 are shown with allele-specific gene expression (represented by yellow spheres) for cell ValaB8w3019, as reported in the HiRES paper [14]. The size of each yellow sphere is proportional to the expression level detected by HiRES. CpG frequency is color-coded on the chromosome structure, with green indicating higher CpG content and magenta indicating lower CpG content. The same as in the HiRES paper [14], the CpG frequency was used as a proxy of euchromatin and heterochromatin or A/B compartments.

Using SCW (two-stage, 10 Mbp first, and then 1 Mbp), we generated 20 independent 3D structural reconstructions for cell ValaB8w3019, and the average values of these 20 structures are displayed in Fig. 11B. In Fig. 11B, the boxplots summarize the distances of the A/B compartment regions to the nuclear center. It can be found that regions with higher CpG content (green or A compartment) are generally located closer to the nuclear center compared to regions with lower CpG content (magenta or B compartment), with statistically significant differences (*P* value = 0). This finding was also observed in HiRES [14], which further validated the SCW-generated 3D genome structures.

### Benchmark of reproducibility

**Fig. 12:**
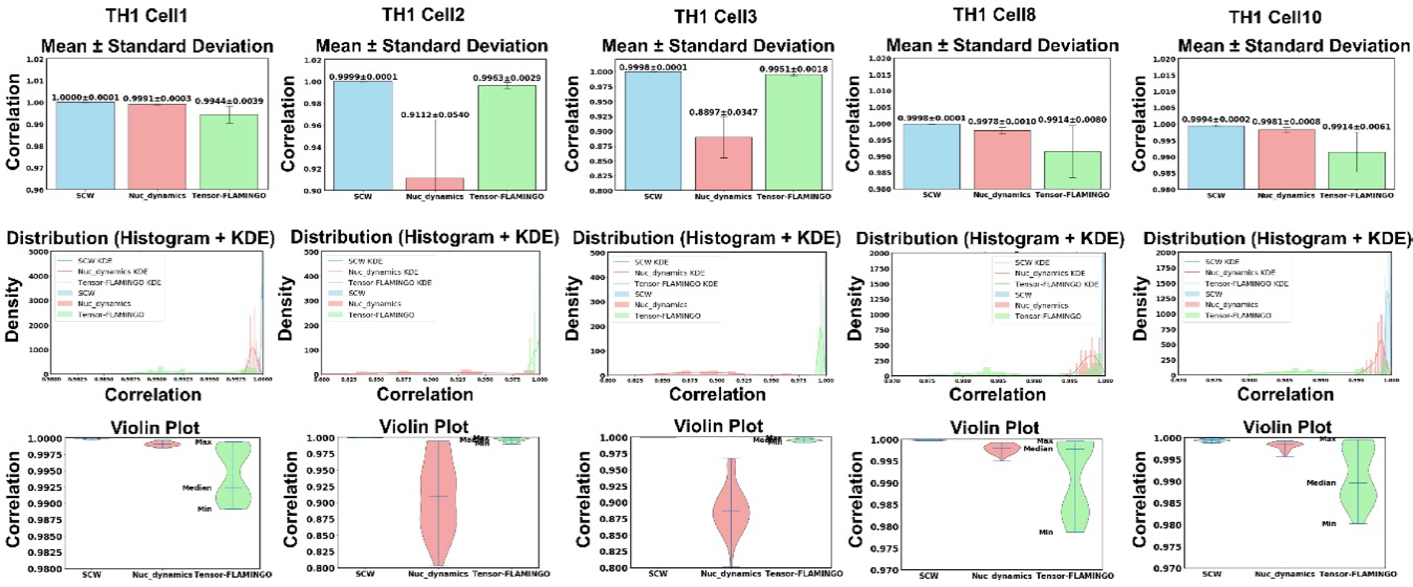
3D coordinates correlation across 20 structural models for 5 TH1 cells at 500 Kbp resolution, structures generated by SCW, Nuc_dynamics, and Tensor-FLAMINGO

We reconstructed 20 haploid 3D chromatin structures at 500 Kbp resolution for five different and randomly chosen TH1 cells using SCW (two-stage, 10 Mbp first, and then 500 Kbp), Nuc_dynamics, and single-input Tensor-FLAMINGO. For SCW and Nuc_dynamics, we obtained whole-genome 3D structures, whereas for Tensor-FLAMINGO, which can only reconstruct structures for individual chromosomes, we specifically modeled chromosome 4 (randomly chosen) in all cells. Figure 12 shows the number of times or frequencies of observing a certain Pearson’s correlation value when pairwise comparing two structures. Although the performance of Tensor-FLAMINGO and Nuc_dynamics was comparable and varied from cell to cell, SCW was notably superior and more stable. It exhibited the highest mean and median correlation values, as well as the lowest standard deviation.

To further assess its robustness, we applied SCW to five randomly chosen NXT cells and consolidated representative results in Supplementary Fig. S126, which confirm its consistent performance. Supplementary Fig. S126 shows that SCW was the top-performing and most robust tool overall. This conclusion holds despite its mean correlation being marginally lower (by < 0.0006) in one of the five cells and slightly larger standard deviations in three cells (by 0.0009, 0.0001, and 0.0003) when compared to Nuc_dynamics. In its leading cases, SCW achieved substantially greater advantages, with superior mean correlations (by up to 0.1) and lower standard deviations (by up to 0.003).

We extended the same analysis to five randomly selected individual chromosomes from the TH1 cell1 and NXT896 cells, as detailed in Supplementary Figs. S127 and S128. On these ten chromosomes from two different cells, SCW constantly have higher mean values and lower standard deviations than Nuc_dynamics and Tensor-FLAMINGO, except for a higher standard deviation in one out of ten cells compared to Nuc_dynamics (by 0.0011). The results consistently confirmed that SCW provides the best overall reproducibility among the three tools, validating our primary findings.

### Computational time

SCW utilizes multithreading to generate 3D structures. For example, generating 500 Kbp resolution NXT896 structures using SCW with a single thread took approximately 12 h. However, using 40 threads reduces the computation time to around 4 h. The CPUs used in this study are 2.20 GHz Intel Xeon E5-2650 v4.

## Discussion

In this study, we developed a new method named SCW that can generate whole-genome 3D structures based on extremely sparse single-cell Hi-C data. We parallelized the computationally intensive parts of the tool, making it possible to infer higher-resolution whole-genome structures. Using SCW, we generated the whole-genome 3D structures for different types of human and mouse, diploid and haploid cells, with only single-cell Hi-C contact data as input. Our inferred 3D structures were evaluated by comparing the parsed Euclidean distances with the corresponding Hi-C data and the fitness to fractal globule or equilibrium globule structures. Furthermore, the following evaluations were performed: comparison of radial positioning patterns between SCW-generated structures and published DNA FISH data, benchmarking against ground truth 3D structures derived from bulk Hi-C, and cross-validation of spatial organization with independently defined A/B compartments. Benchmarking against established modeling tools Nuc_dynamics, Tensor-FLAMINGO, and Hickit through these evaluation frameworks demonstrates that SCW provides a more accurate, user-friendly (only Hi-C data as input), reproducible, and broadly applicable platform for single-cell whole-genome structural modeling.

## Supplementary Information

Below is the link to the electronic supplementary material.

Supplementary Material 1

## Data Availability

The C+ + source code of SCW is freely available at http://dna.cs.miami.edu/SCW/ and [https://github.com/zwang-bioinformatics/SCW/] (https://github.com/zwang-bioinformatics/SCW). Mouse G1 haploid single-cell Hi-C data were downloaded from the Gene Expression Omnibus (GEO) under accession number GSE94489. GM12878 and PBMC diploid single-cell Hi-C data were downloaded from the GEO under accession number GSE117876. Whole-genome human bulk Hi-C data were downloaded from the GEO under accession number GSE63525. Simultaneous single-cell Hi-C and single-cell gene expression data were downloaded from the GEO under accession number GSE223917. Mouse 3D-FISH data were obtained from Supplementary Tables 4 and 5 of the paper [18]. Human FISH data were obtained from Fig. 2B of the paper [20].
