# Supplementary material for: SCW: building the whole-genome 3D structures based on extremely sparse single-cell Hi-C data
Source: BMC Bioinformatics. 2026 Mar 17;27:90. doi: 10.1186/s12859-026-06421-3 (PMC13107861; doi:10.1186/s12859-026-06421-3)
Supplement: Supplementary file 1 — Supplementary Material 1 [file 12859_2026_6421_MOESM1_ESM.pdf]

Supplementary documents for

SCW: building the whole-genome 3D structures based on extremely sparse single-cell Hi-C data

AUTHORS

Hao Zhu[1], Tong Liu[2], Bishal Shrestha[2], and Zheng Wang[2,*]

[1] Department of Computer Science, Florida Memorial University, 15800 NW 42nd Ave, Miami Gardens, FL, 33504, US

[2] Department of Computer Science, University of Miami, 330M Ungar Building, 1365 Memorial Drive, Coral Gables, FL, 33124-4245, US

* To whom correspondence should be addressed. Email: zheng.wang@miami.edu

Present Address: Zheng Wang, Department of Computer Science, University of Miami, 330M Ungar Building, 1365 Memorial Drive, Coral Gables, FL, 33124-4245, US

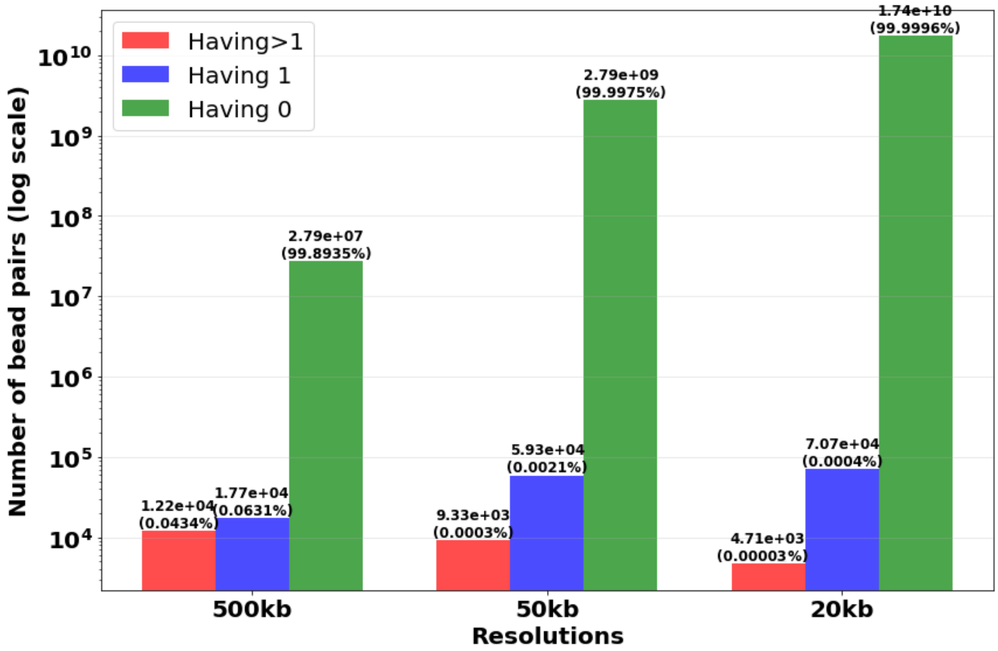

Figure S1 Number of bead pairs having 0, 1, and >1 Hi-C contacts with from the Hi-C contact matrix at three resolutions of NXT896 Hi-C data

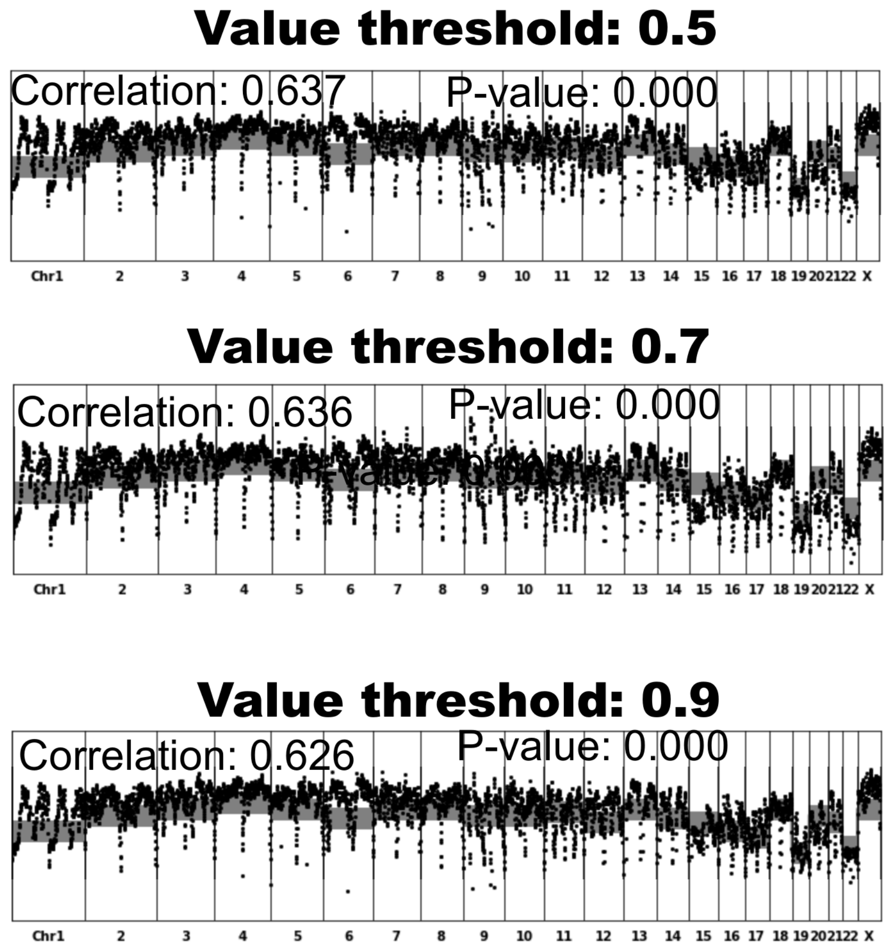

Figure S2 Average distance to the nuclear center of every bead in the 1 Mbp whole-genome structures (black dots) generated by SCW with published DNA FISH data (gray lines) on whole chromosomes for different thresholds.

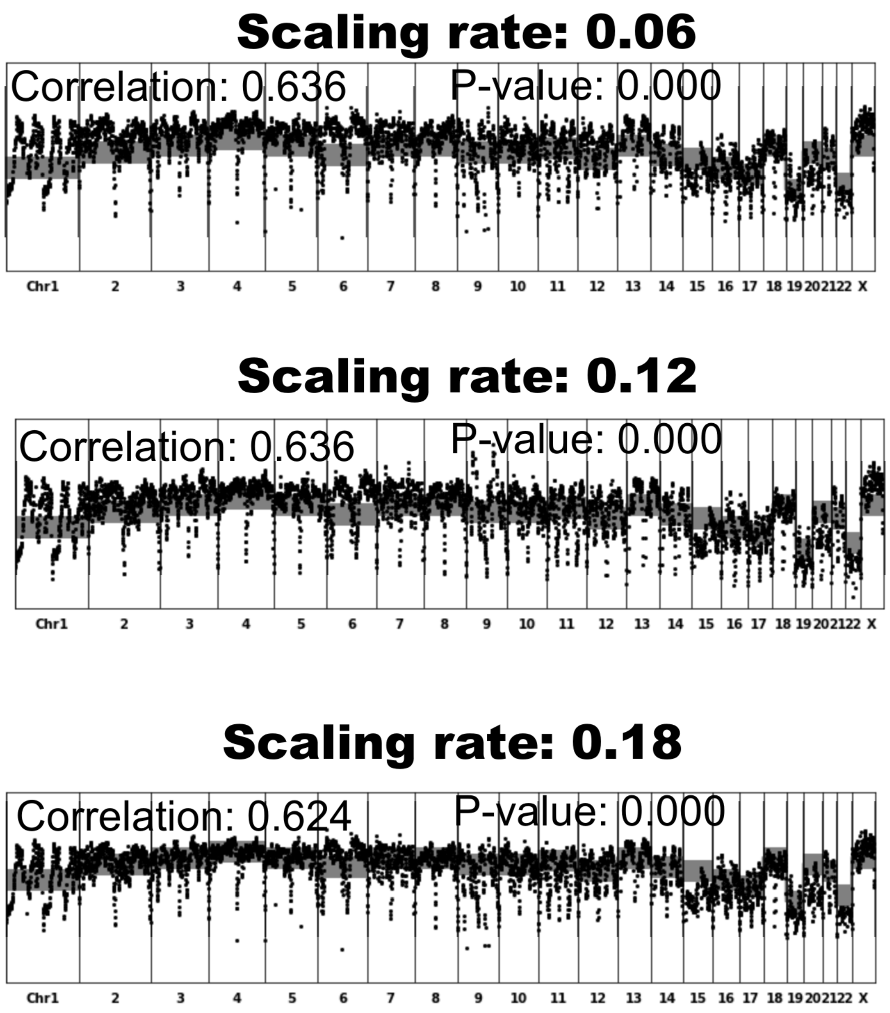

Figure S3 Average distance to the nuclear center of every bead in the 1 Mbp whole-genome structures (black dots) generated by SCW with published DNA FISH data (gray lines) on whole chromosomes for different scaling rates.

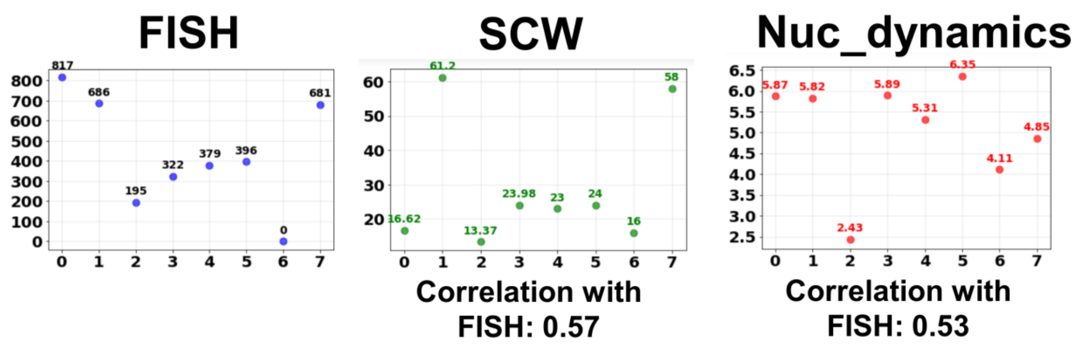

Figure S4 Scatter plots for the original FISH data, distances of the same bead pairs from SCW-inferred structure and Nuc_dynamics-inferred structure

The following figures (S5-S34) are the SCW-inferred 3D structures at 1 Mbp resolution based on the Hi-C dataset in the experiment [1]

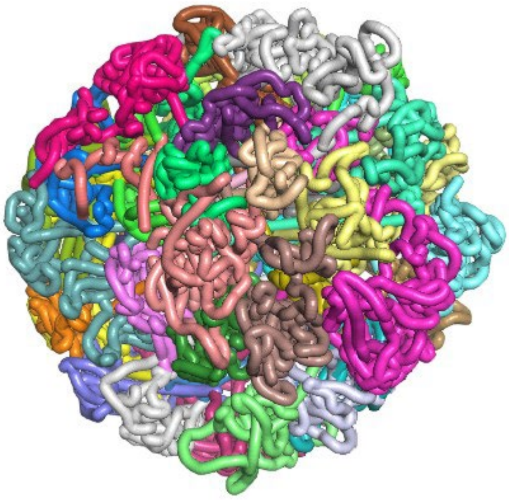

Figure S5 GM12878 Cell 2

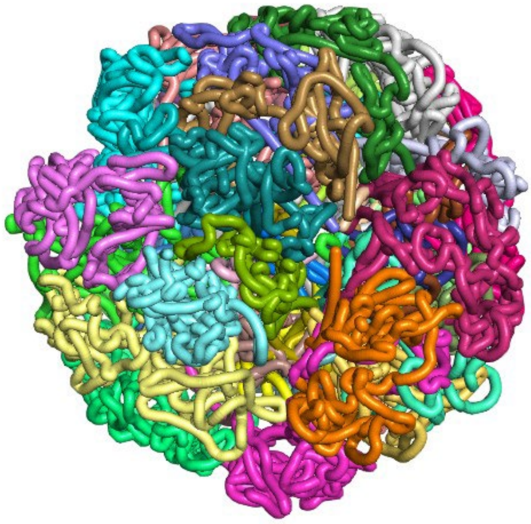

Figure S6 GM12878 Cell 3

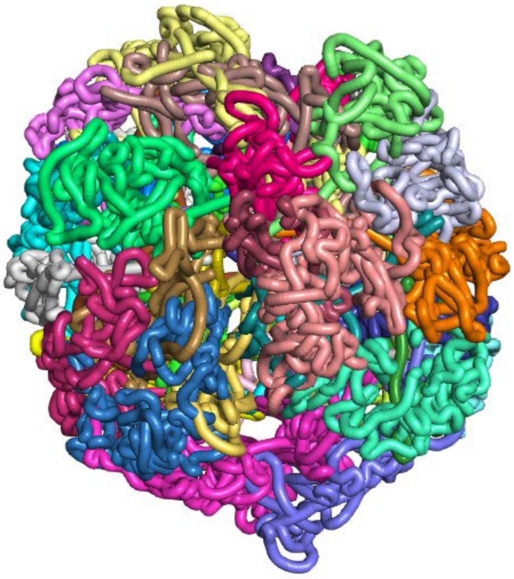

Figure S7 GM12878 Cell 5

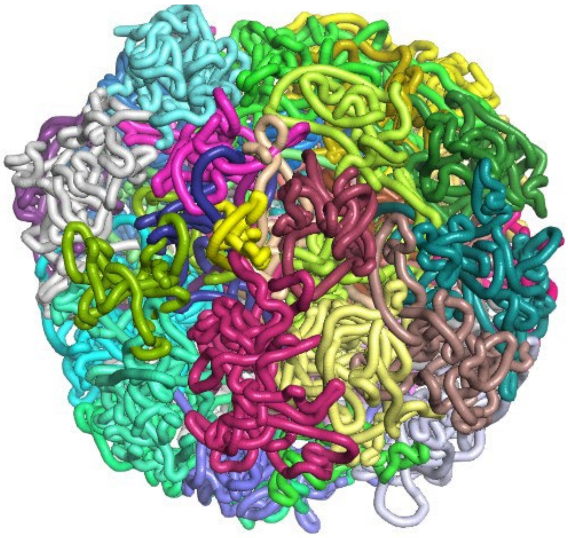

Figure S8 GM12878 Cell 6

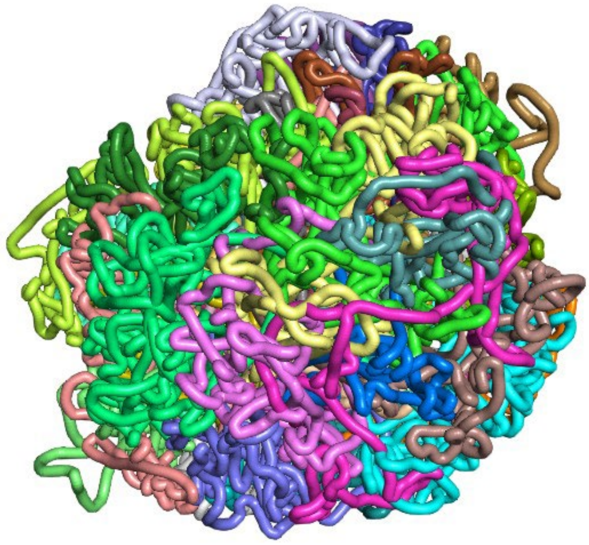

Figure S9 GM12878 Cell 7

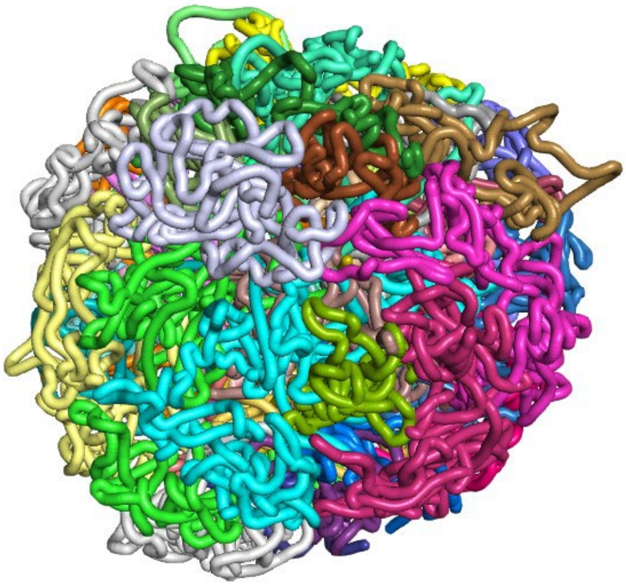

Figure S10 GM12878 Cell 9

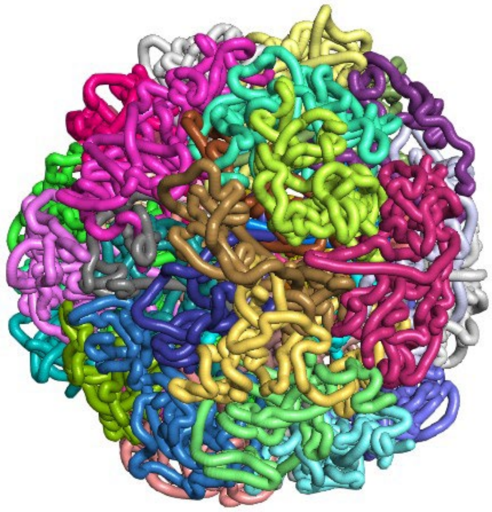

Figure S11 GM12878 Cell 12

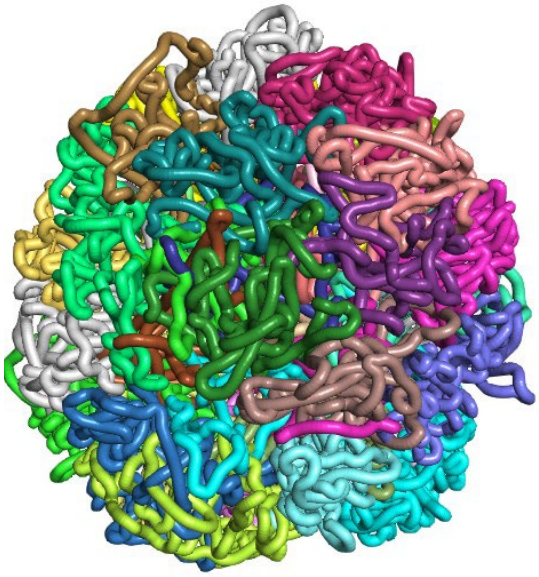

Figure S12 GM12878 Cell 13

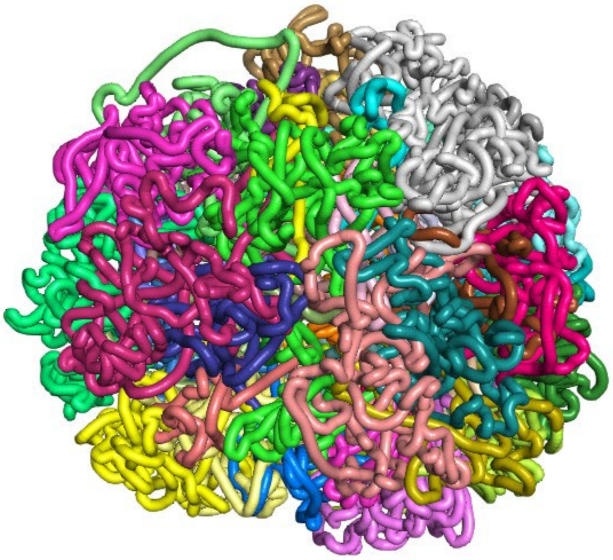

Figure S13 GM12878 Cell 14

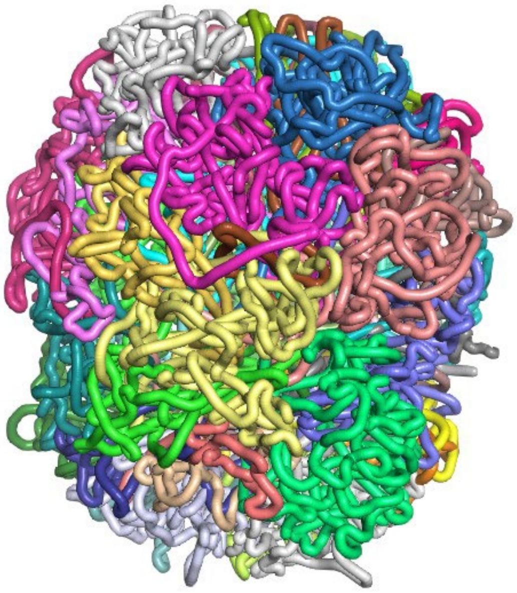

Figure S14 GM12878 Cell 15

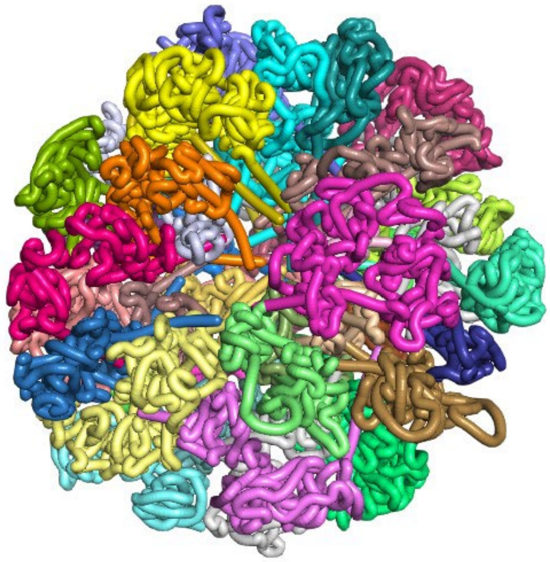

Figure S15 GM12878 Cell 16

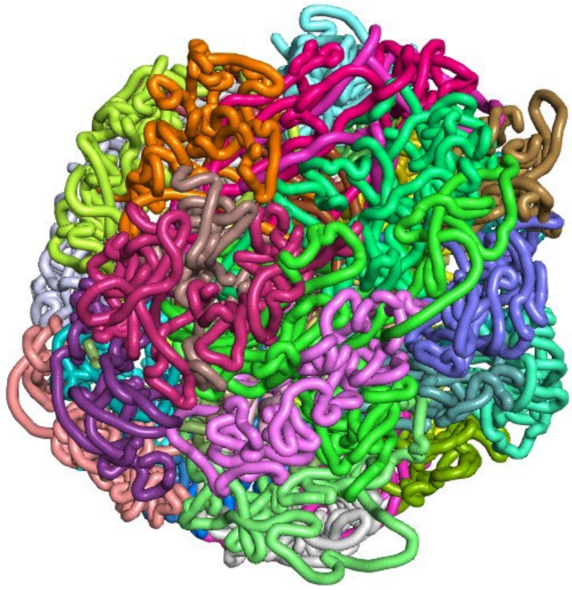

Figure S16 GM12878 Cell 17

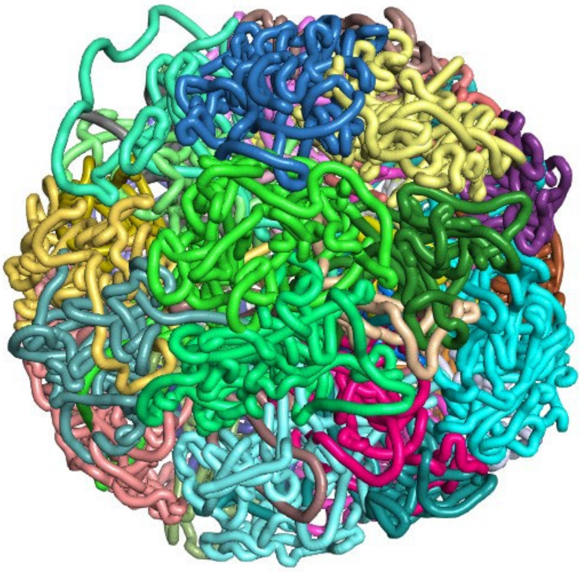

Figure S17 PBMC Cell 1

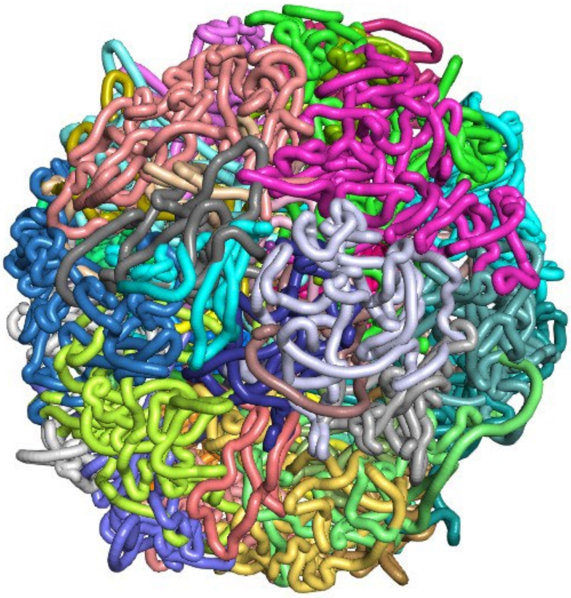

Figure S18 PBMC Cell 1

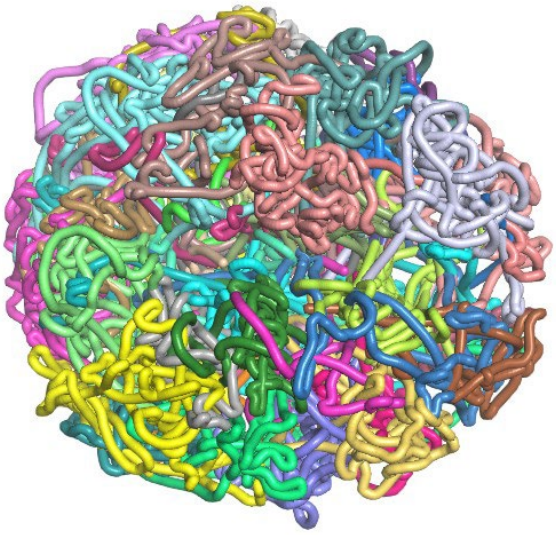

Figure S19 PBMC Cell 3

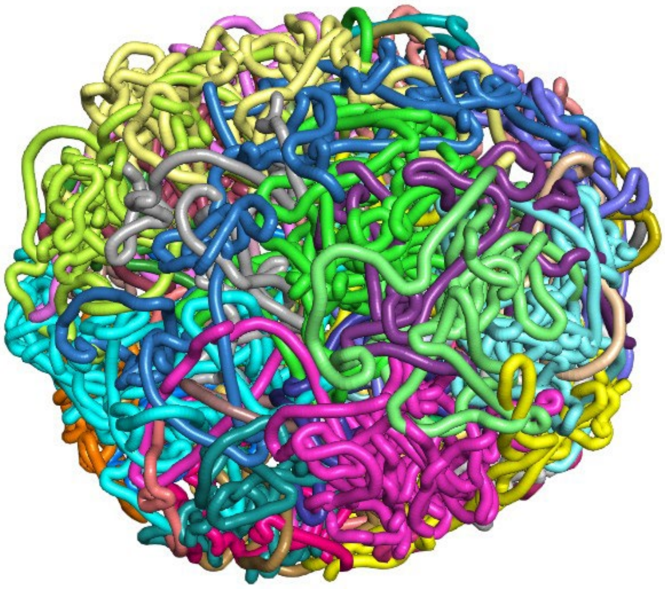

Figure S20 PBMC Cell 4

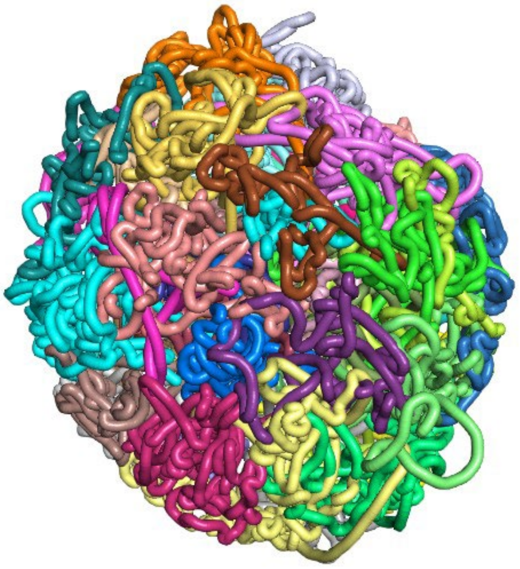

Figure S21 PBMC Cell 5

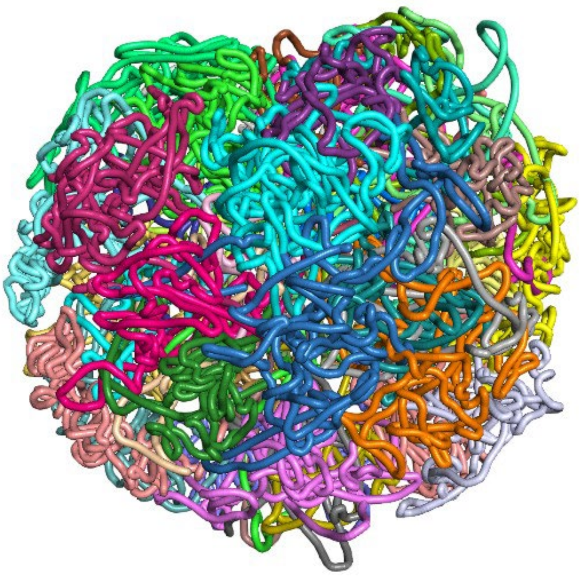

Figure S22 PBMC Cell 6

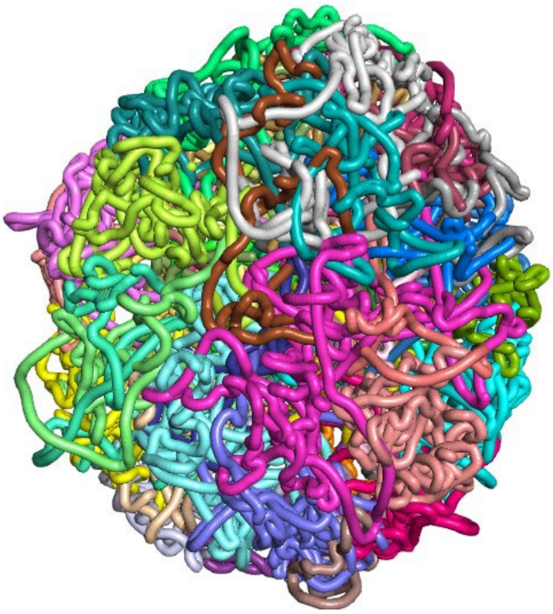

Figure S23 PBMC Cell 7

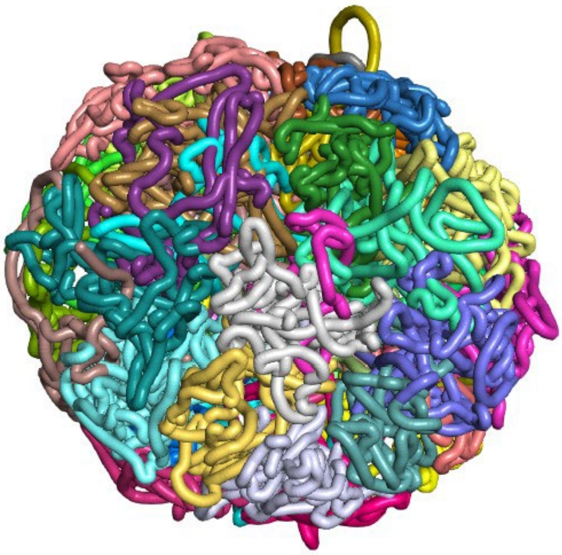

Figure S24 PBMC Cell 8

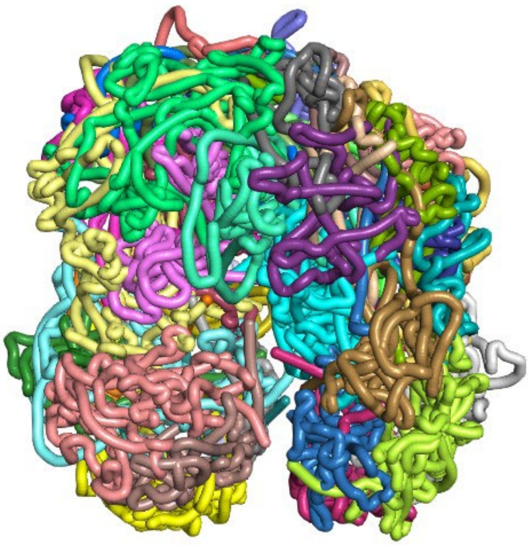

Figure S25 PBMC Cell 9

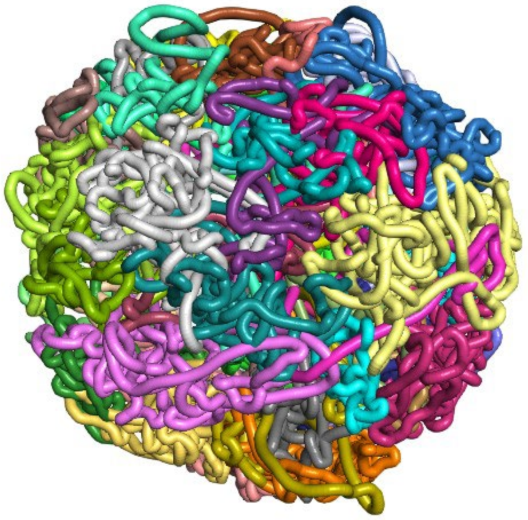

Figure S26 PBMC Cell 10

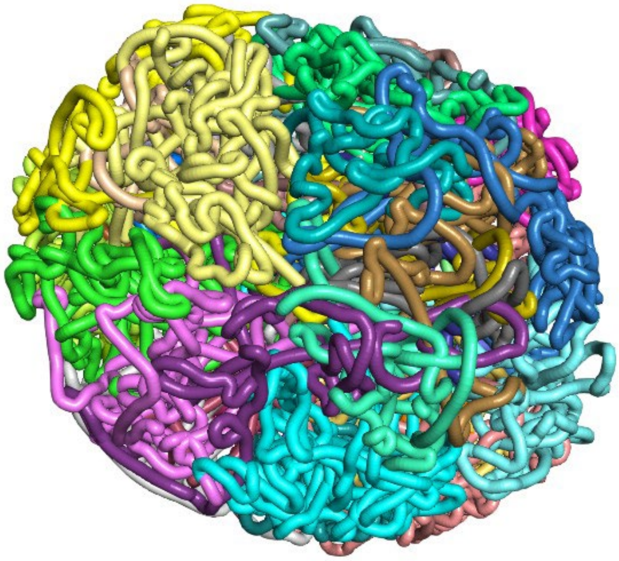

Figure S27 PBMC Cell 11

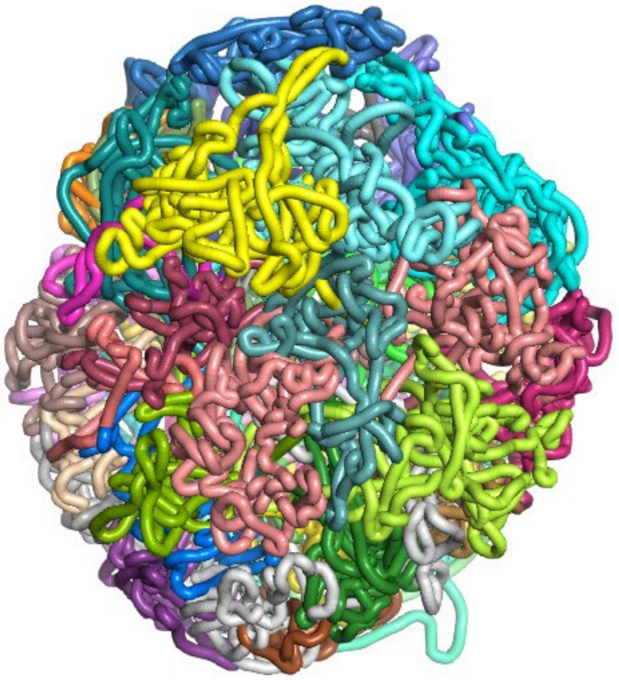

Figure S28 PBMC Cell 12

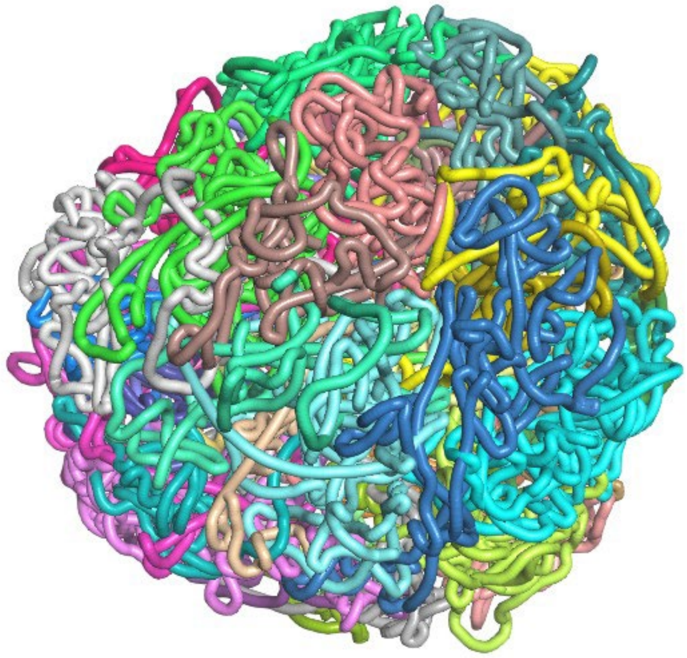

Figure S29 PBMC Cell 13

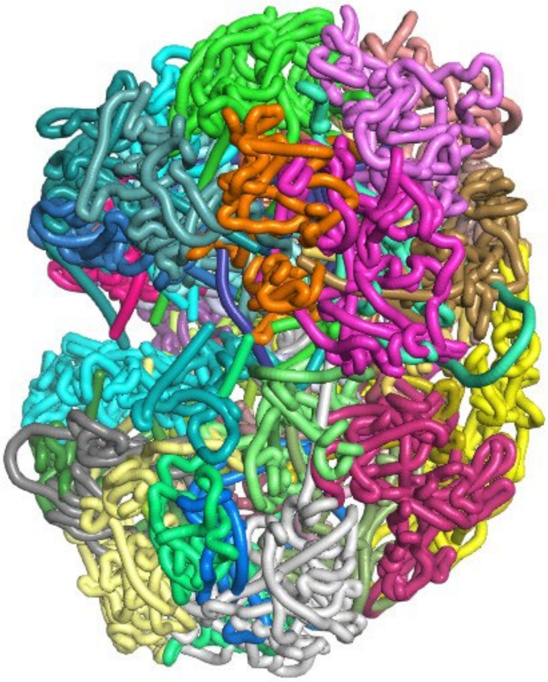

Figure S30 PBMC Cell 14

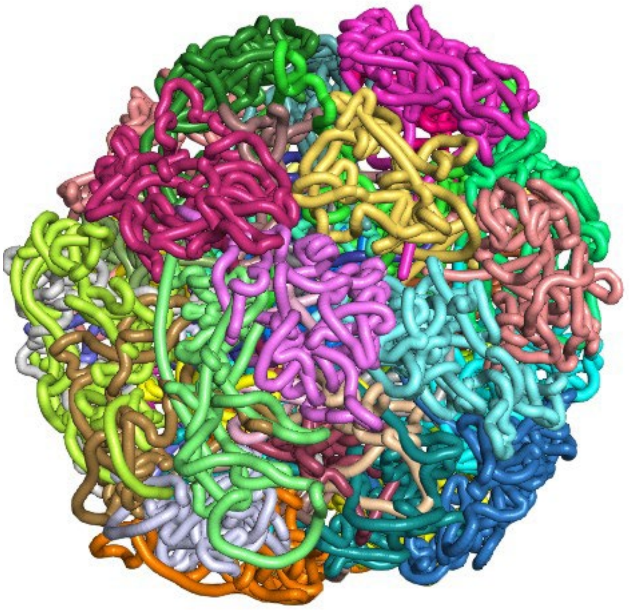

Figure S31 PBMC Cell 15

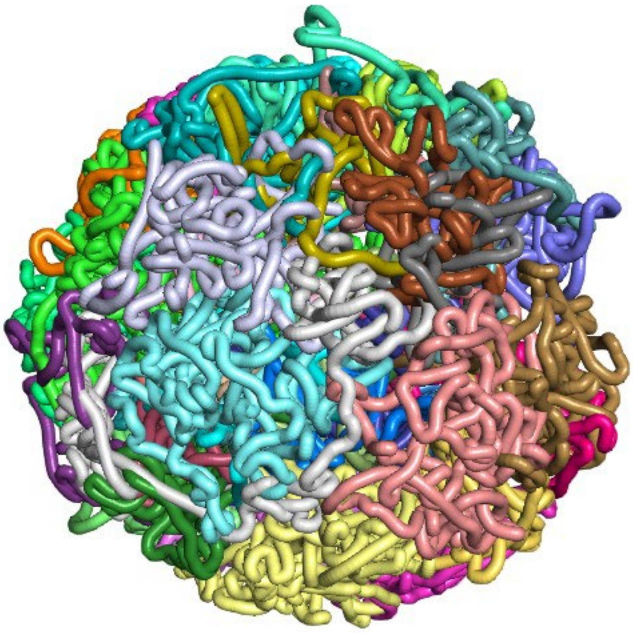

Figure S32 PBMC Cell 16

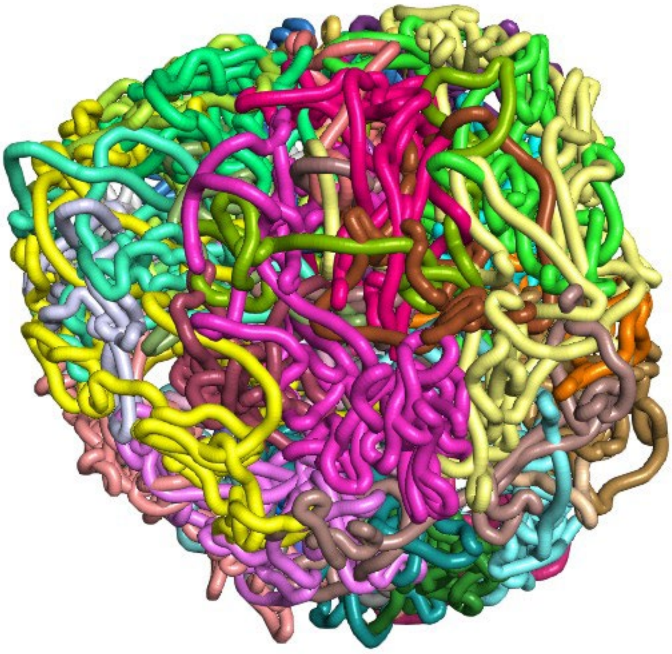

Figure S33 PBMC Cell 17

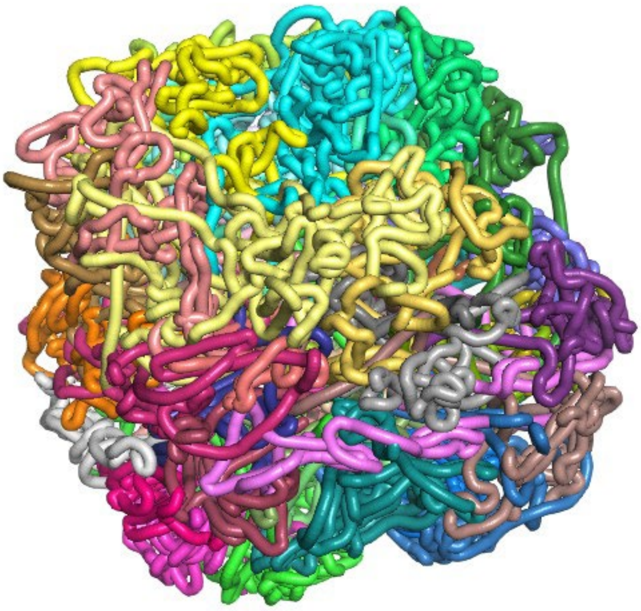

Figure S34 PBMC Cell 18

The following figures (S35-S64) are the superimposed heatmaps of intra-chromosomal Hi-C and distance matrix derived from SCW-inferred structures

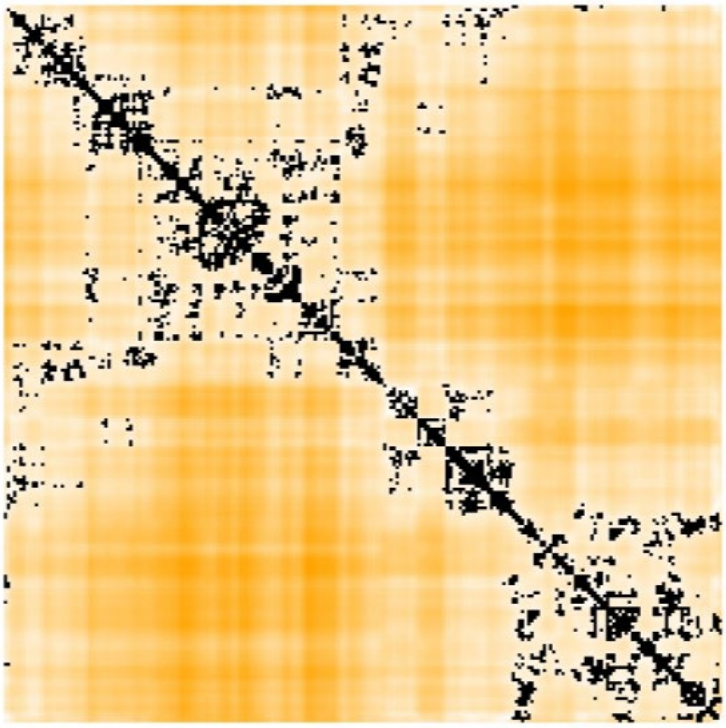

Figure S35 GM12878 Cell 2 Maternal Chr1

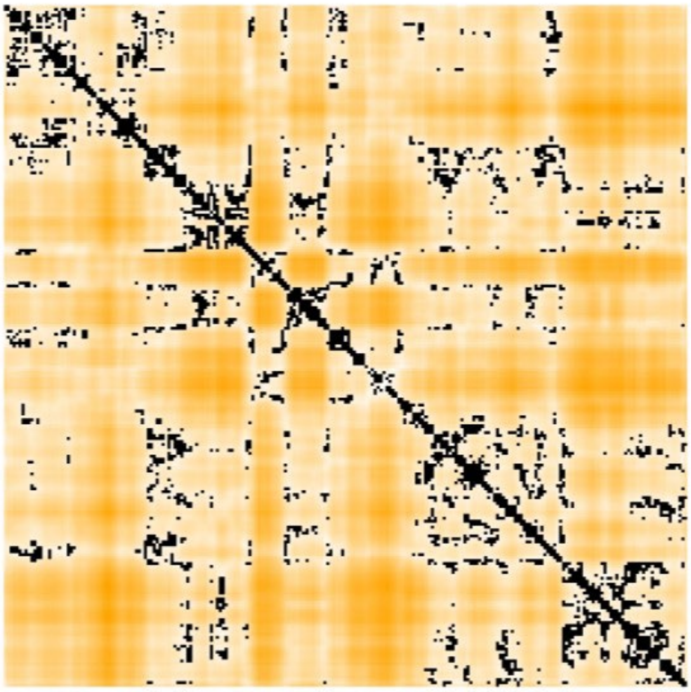

Figure S36 GM12878 Cell 3 Paternal Chr1

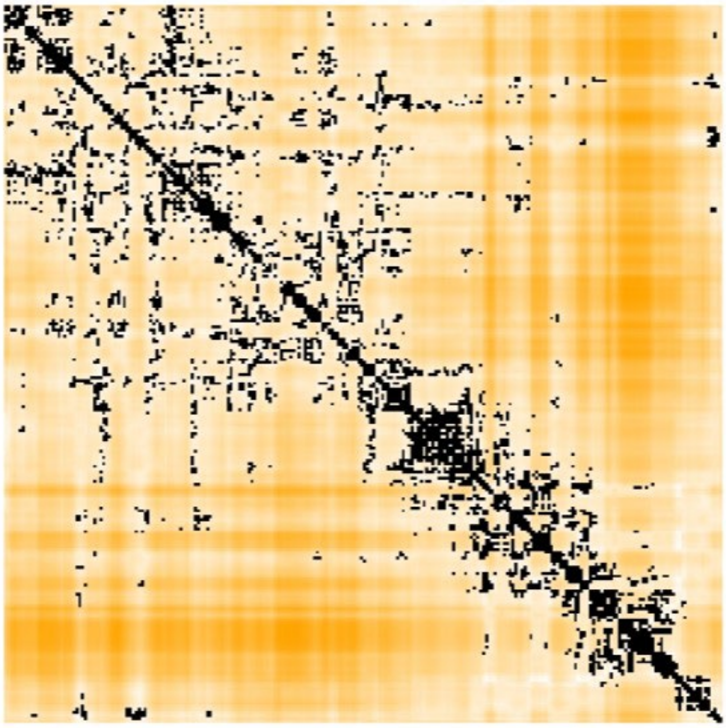

Figure S37 GM12878 Cell 5 Maternal Chr2

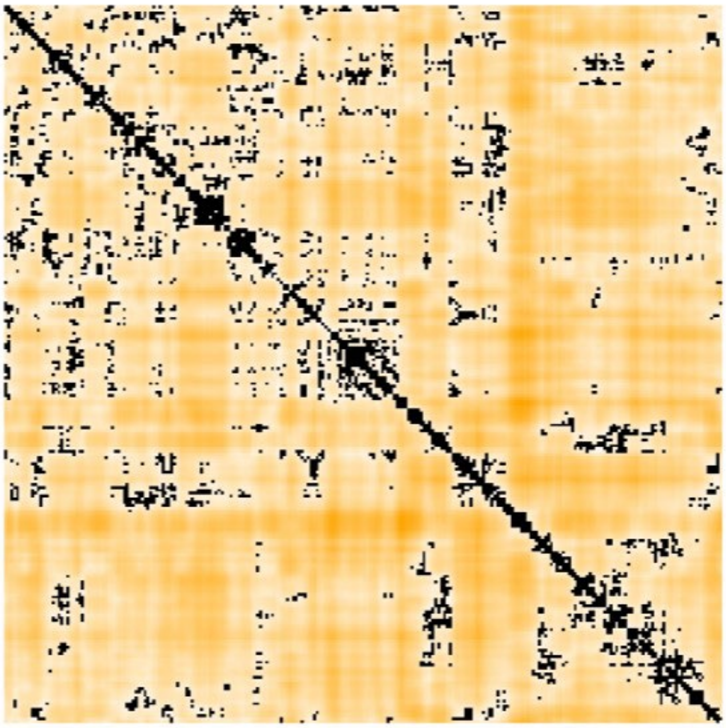

Figure S38 GM12878 Cell 6 Paternal Chr2

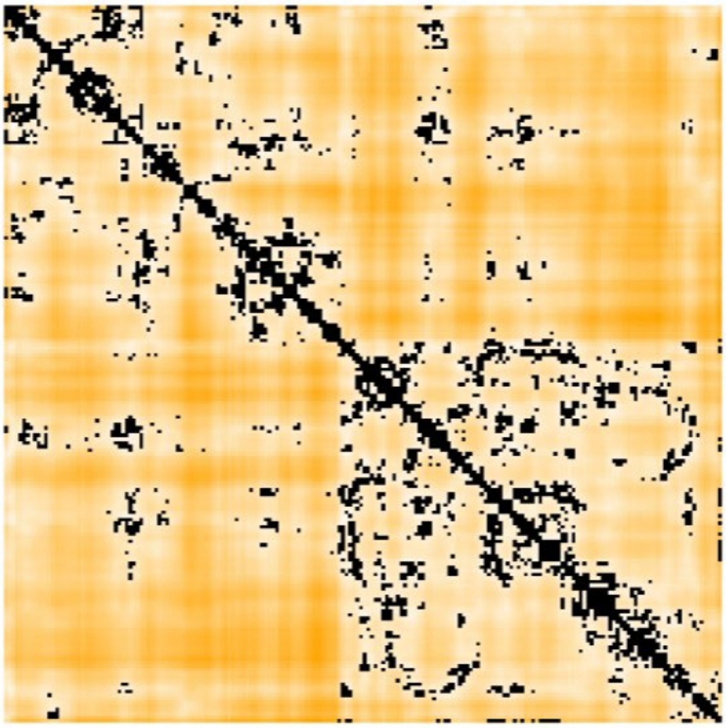

Figure S39 GM12878 Cell 7 Maternal Chr3

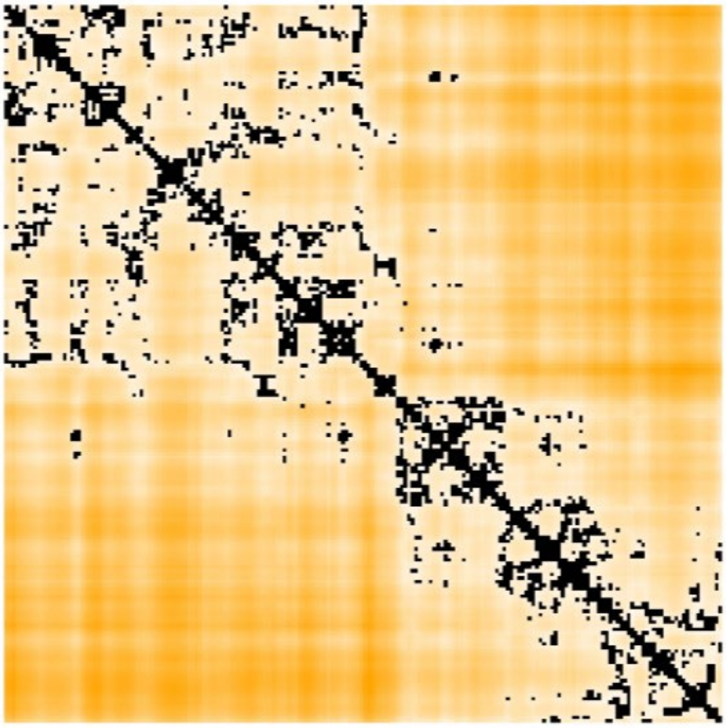

Figure S40 GM12878 Cell 9 Paternal Chr3

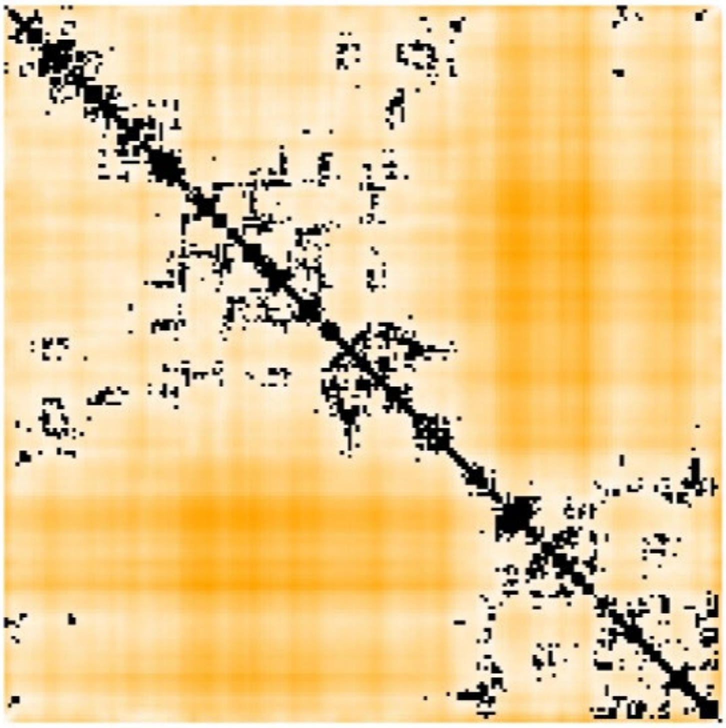

Figure S41 GM12878 Cell 12 Paternal Chr4

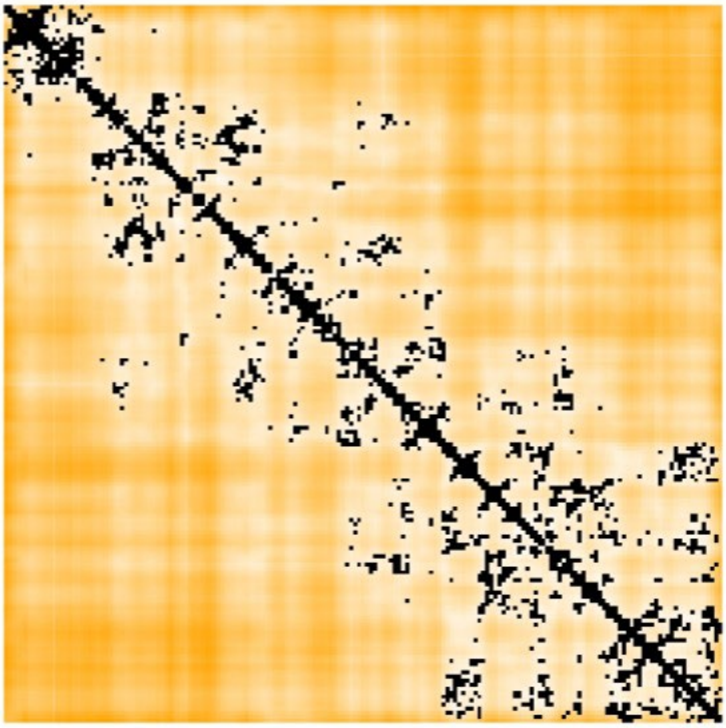

Figure S42 GM12878 Cell 13 Maternal Chr5

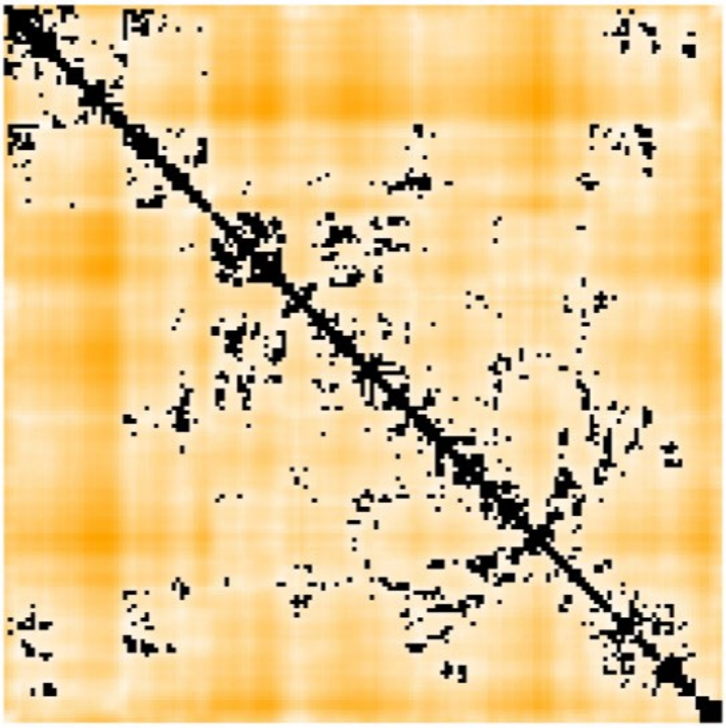

Figure S43 GM12878 Cell 14 Paternal Chr5

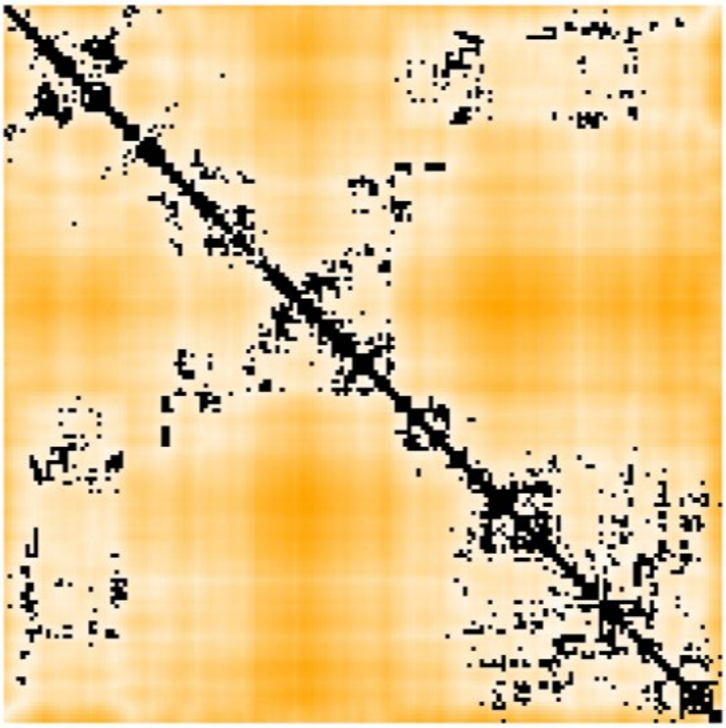

Figure S44 GM12878 Cell 15 Maternal Chr6

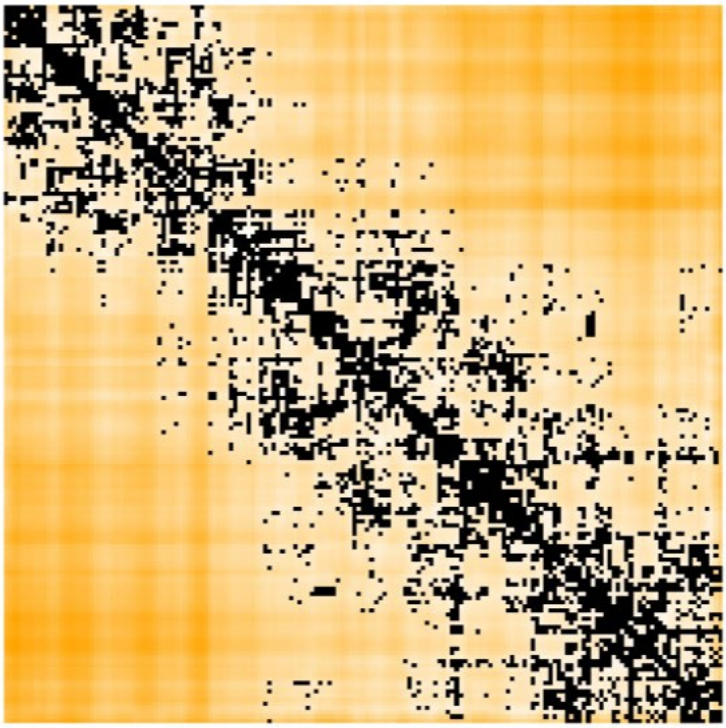

Figure S45 GM12878 Cell 16 Paternal Chr6

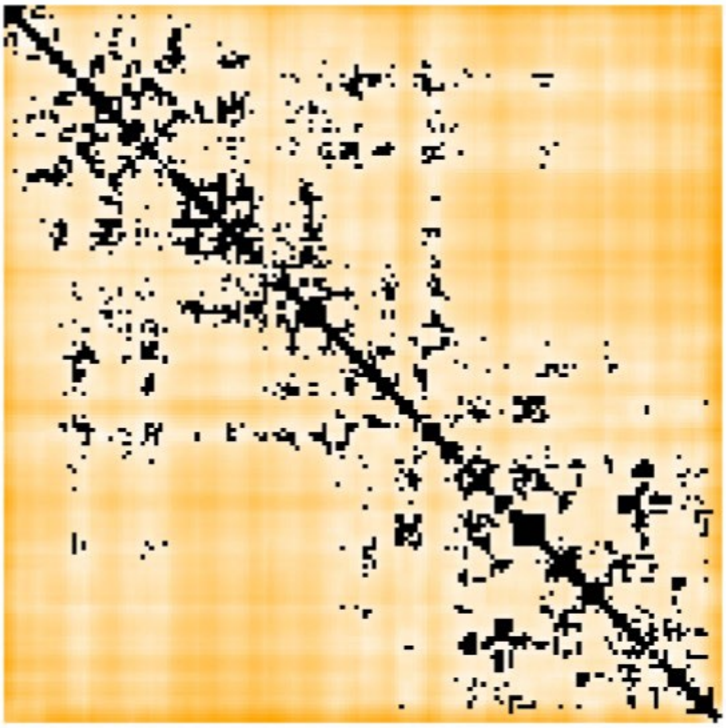

Figure S46 GM12878 Cell 17 Maternal Chr7

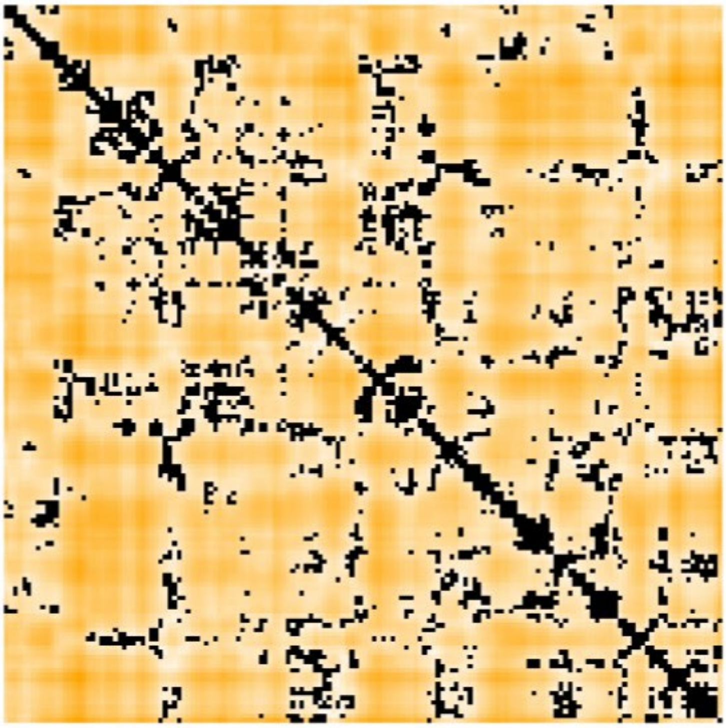

Figure S47 PBMC Cell 1 Paternal Chr7

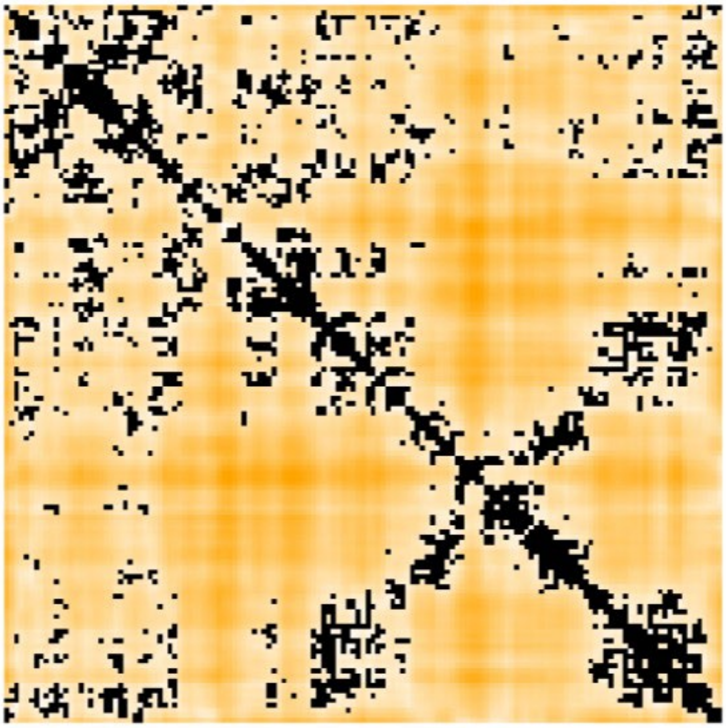

Figure S48 PBMC Cell 2 Paternal Chr8

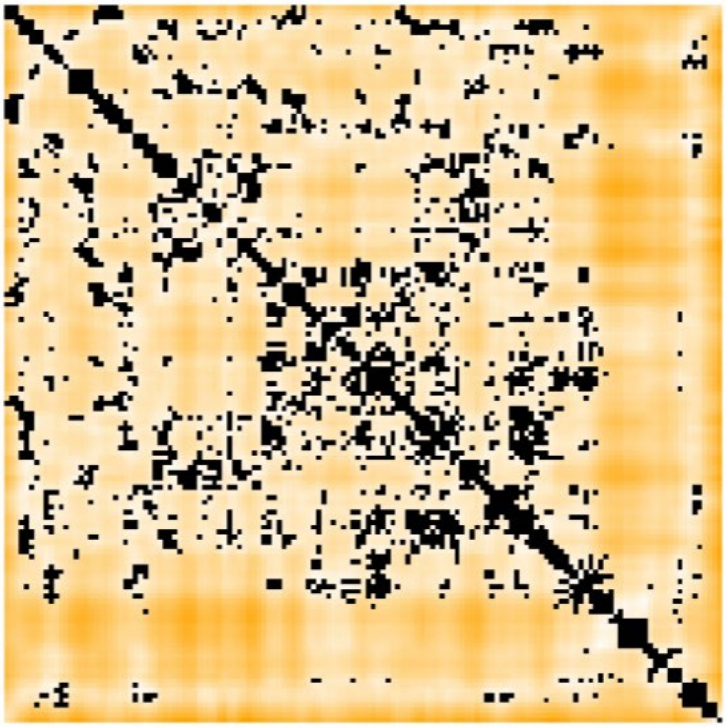

Figure S49 PBMC Cell 3 Maternal Chr8

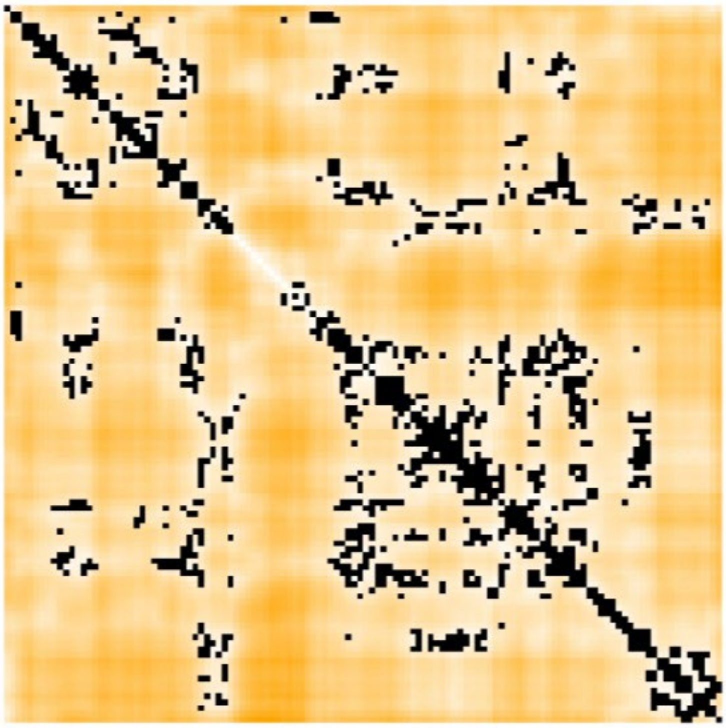

Figure S50 PBMC Cell 4 Paternal Chr9

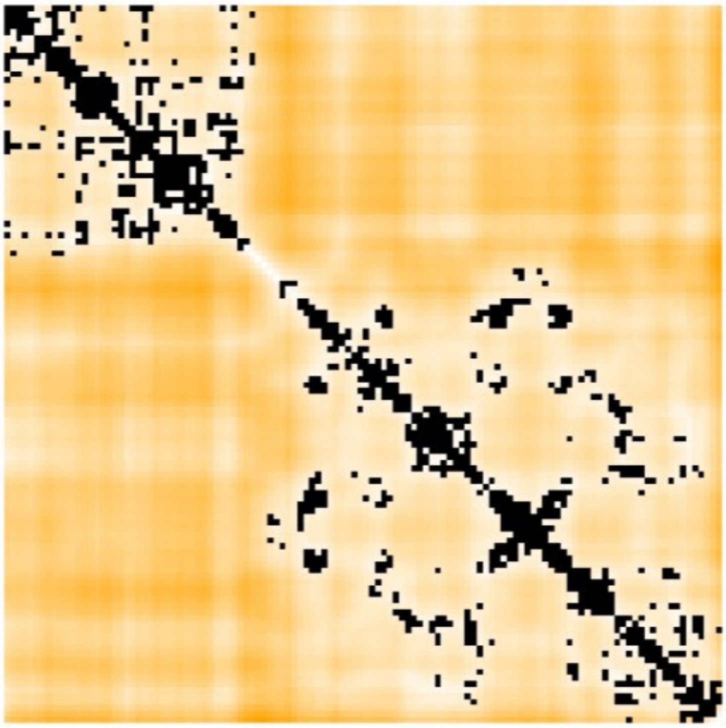

Figure S51 PBMC Cell 5 Maternal Chr9

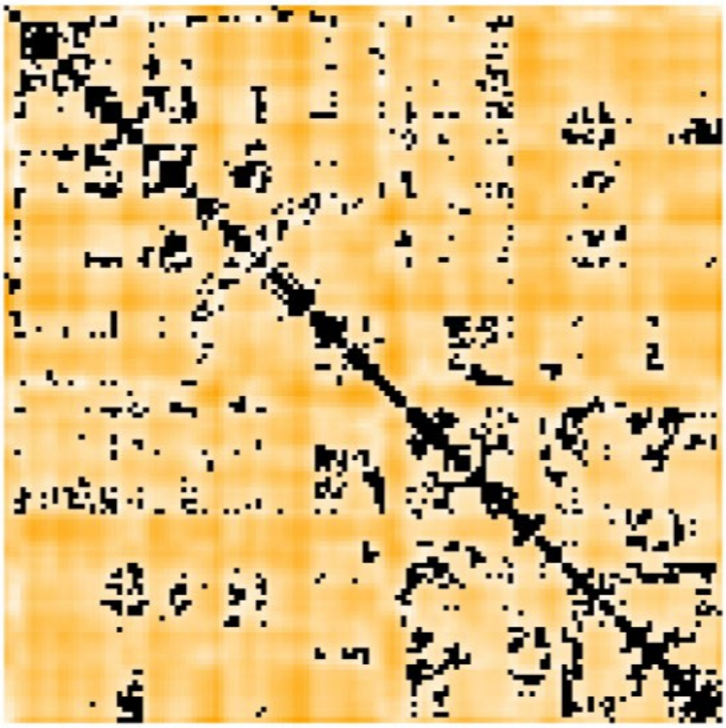

Figure S52 PBMC Cell 6 Paternal Chr10

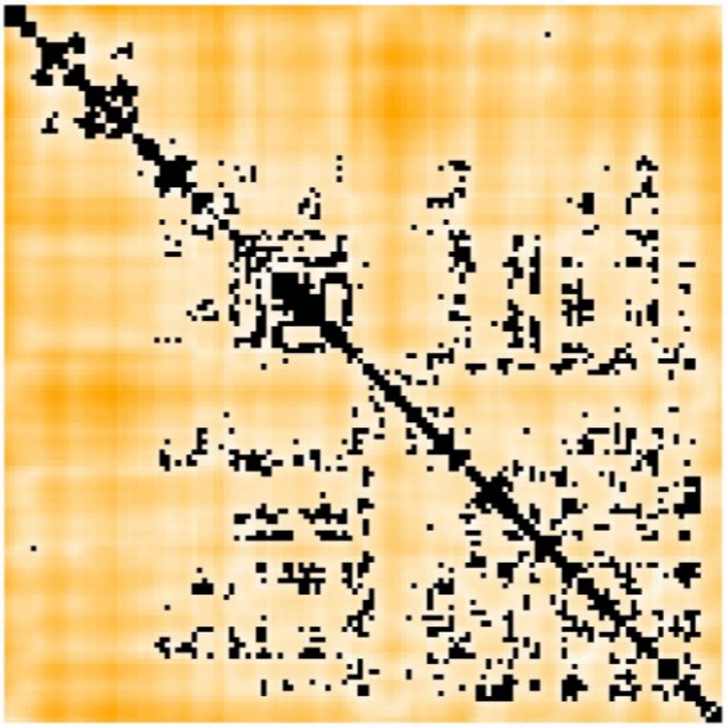

Figure S53 PBMC Cell 7 Maternal Chr10

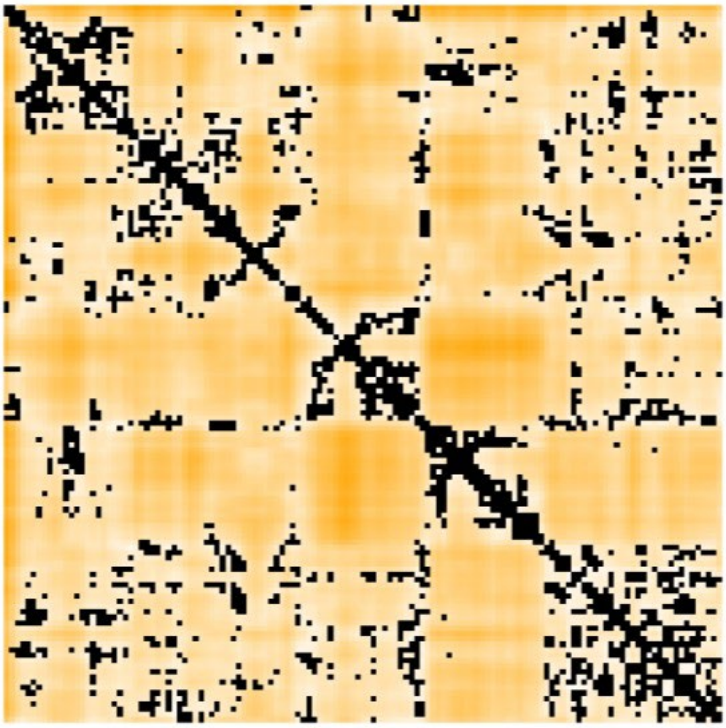

Figure S54 PBMC Cell 8 Paternal Chr11

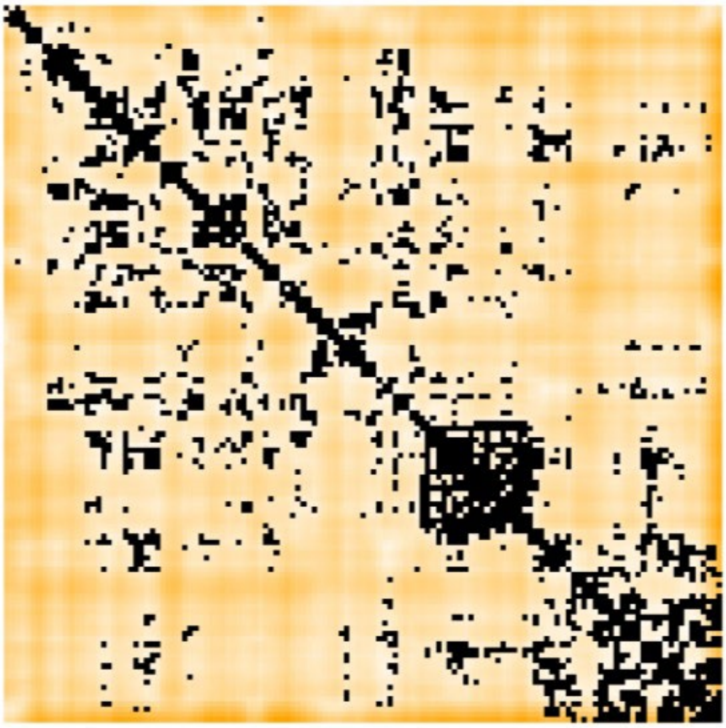

Figure S55 PBMC Cell 9 Maternal Chr11

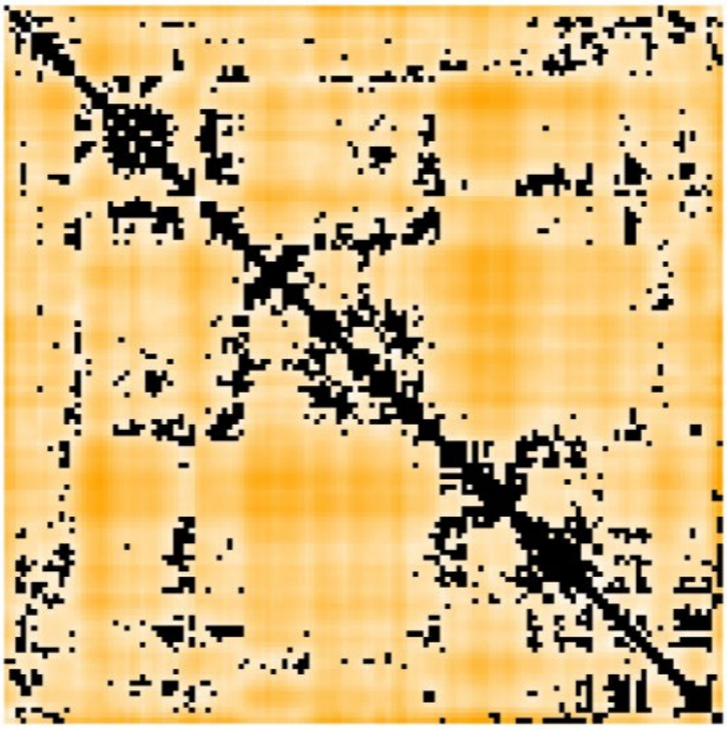

Figure S56 PBMC Cell 10 Paternal Chr12

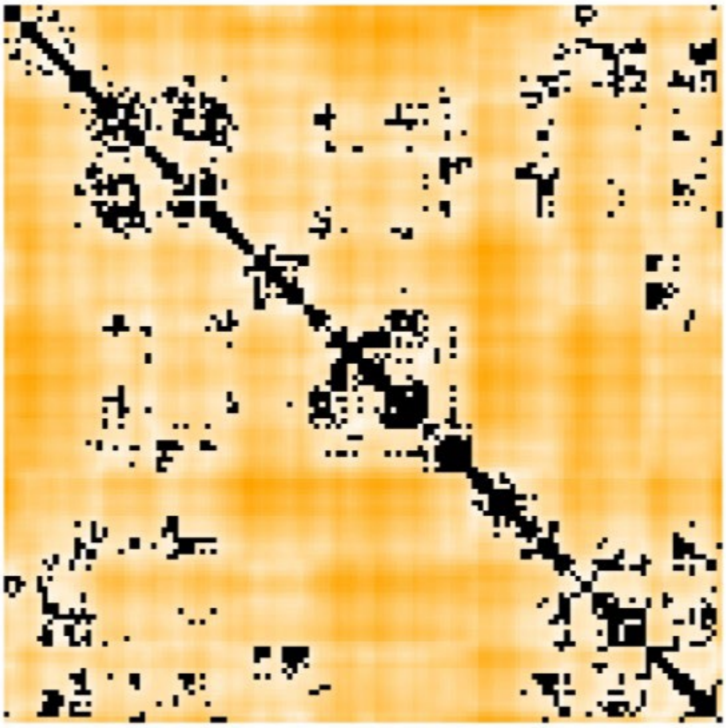

Figure S57 PBMC Cell 11 Maternal Chr12

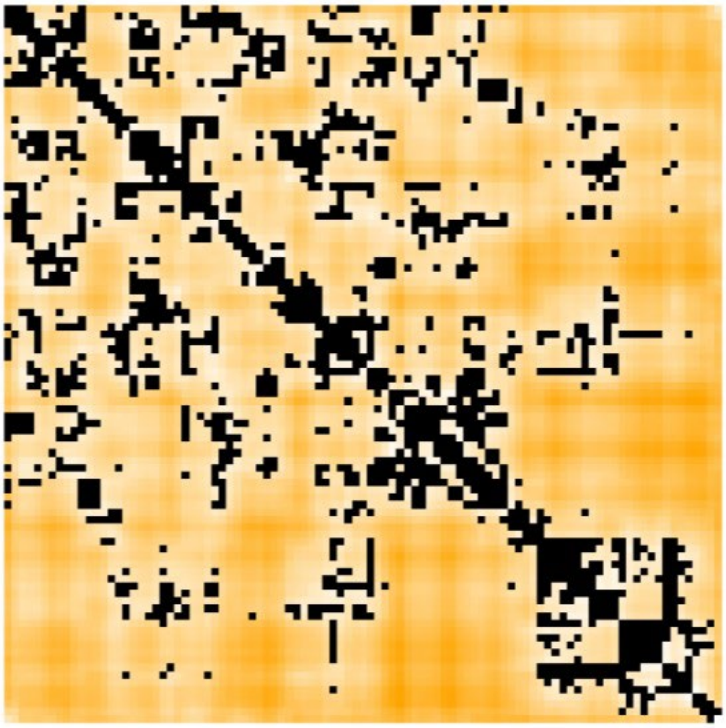

Figure S58 PBMC Cell 12 Paternal Chr13

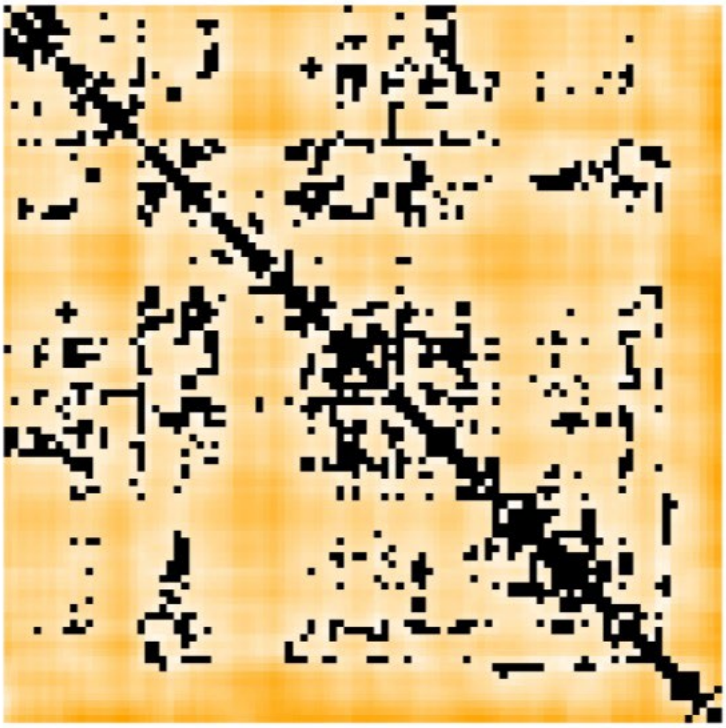

Figure S59 PBMC Cell 13 Maternal Chr13

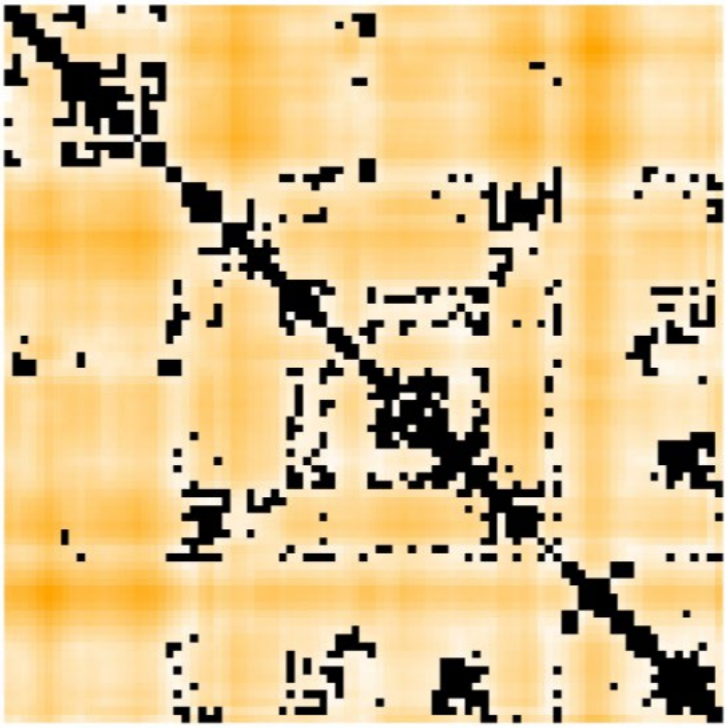

Figure S60 PBMC Cell 14 Paternal Chr14

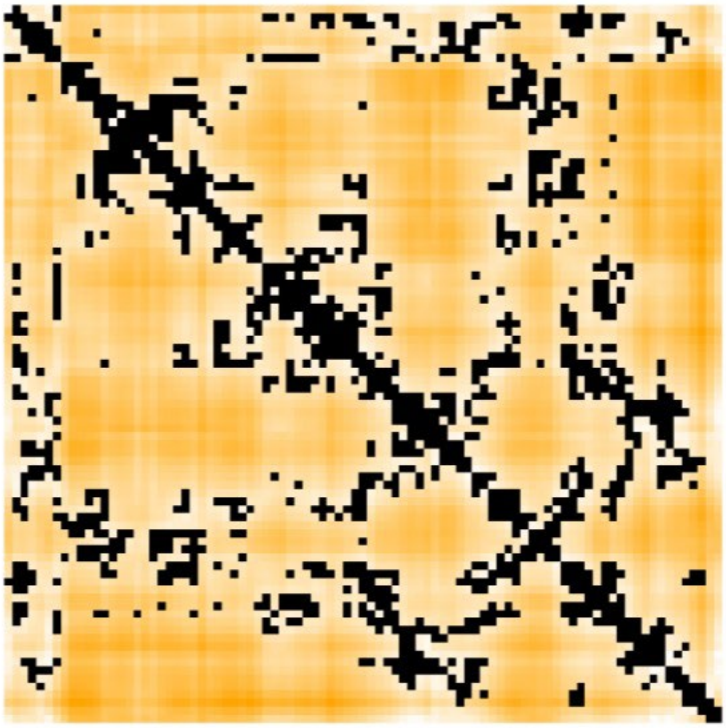

Figure S61 PBMC Cell 15 Maternal Chr14

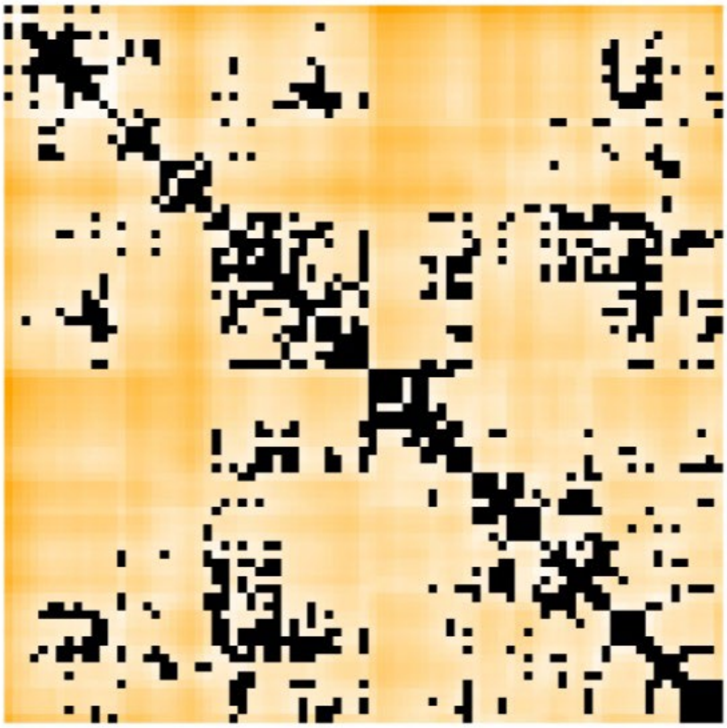

Figure S62 PBMC Cell 16 Paternal Chr15

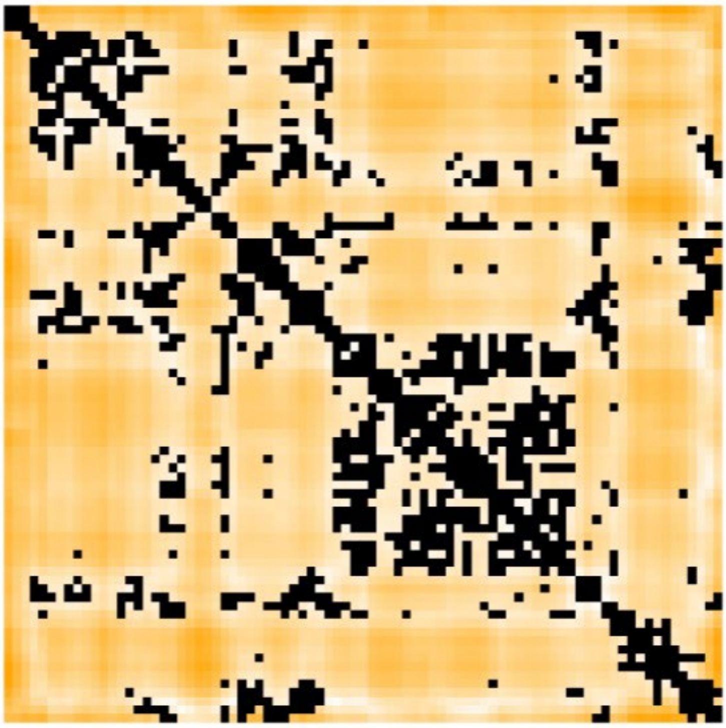

Figure S63 PBMC Cell 17 Maternal Chr15

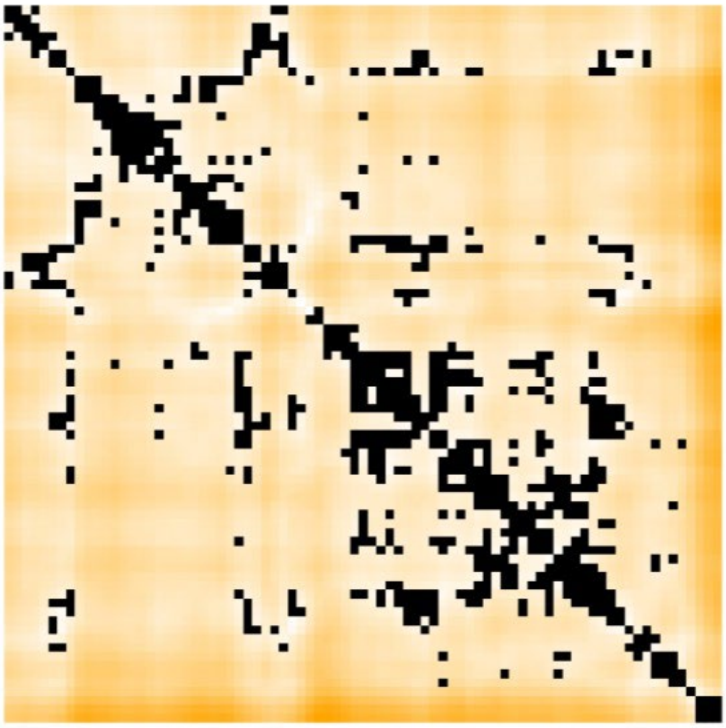

Figure S64 PBMC Cell 18 Paternal Chr16

The following figures (S65-S94) are the superimposed heatmaps of inter-chromosomal Hi-C and distance matrix derived from SCW-inferred structures

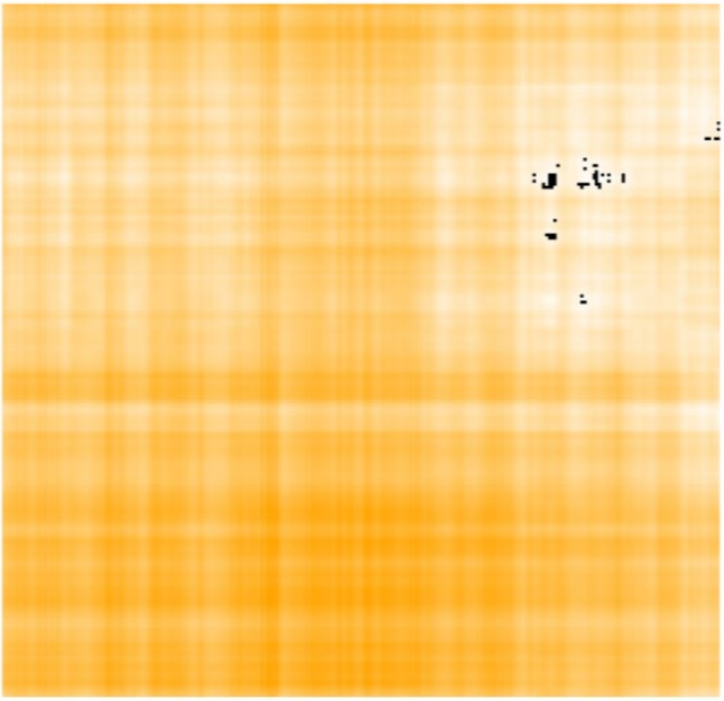

Figure S65 GM12878 Cell 2 Maternal Chr1 and Paternal Chr2

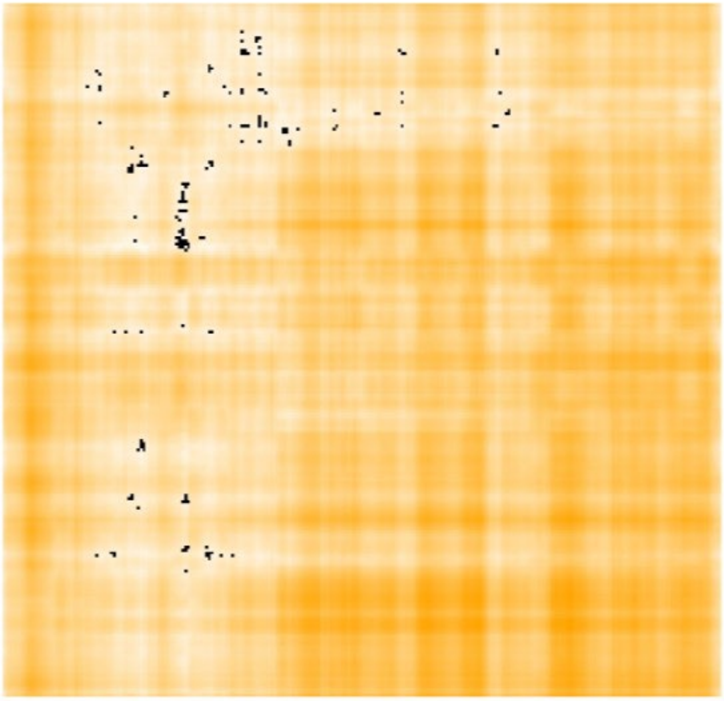

Figure S66 GM12878 Cell 3 Paternal Chr1 and Paternal Chr2

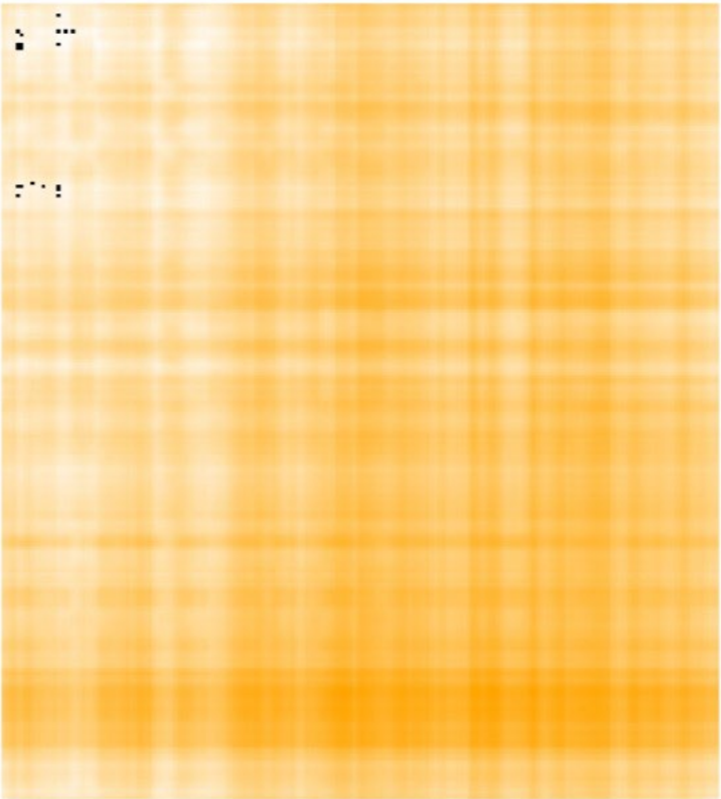

Figure S67 GM12878 Cell 5 Maternal Chr2 and Maternal Chr3

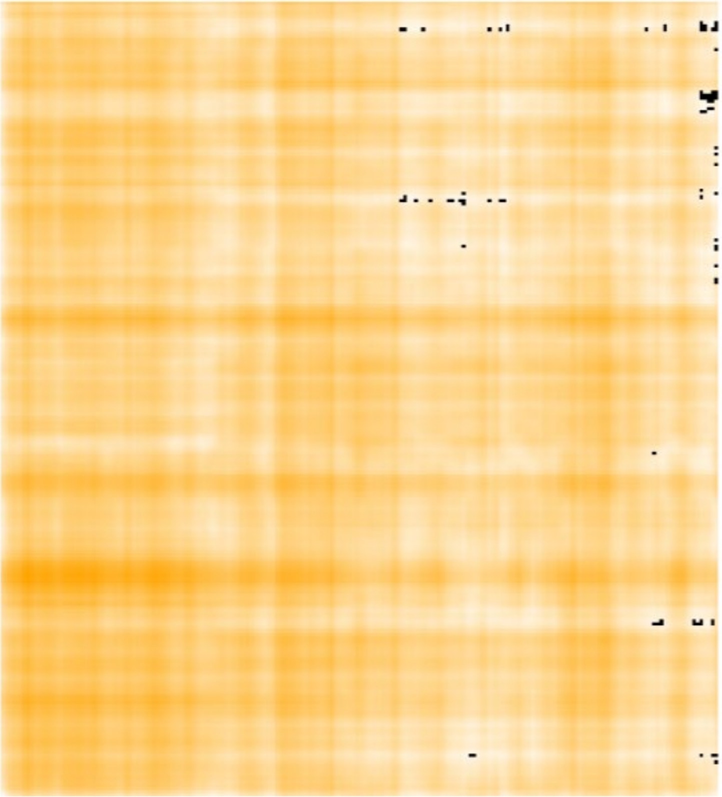

Figure S68 GM12878 Cell 6 Paternal Chr2 and Maternal Chr3

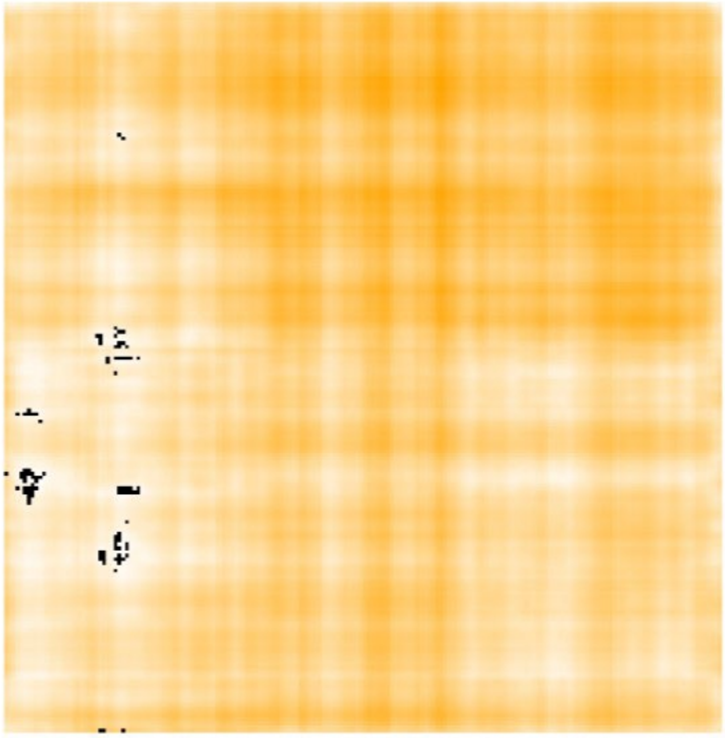

Figure S69 GM12878 Cell 7 Maternal Chr3 and Maternal Chr4

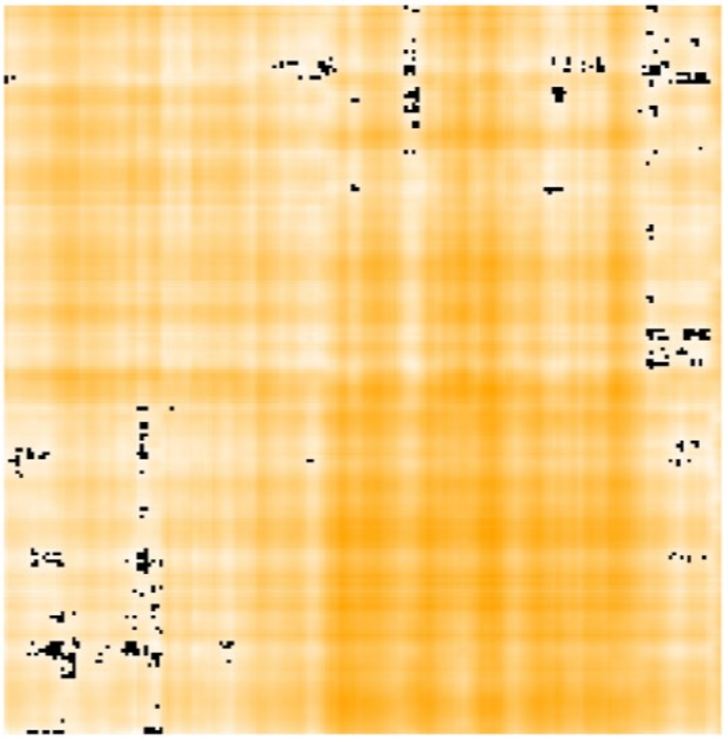

Figure S70 GM12878 Cell 9 Paternal Chr3 and Maternal Chr4

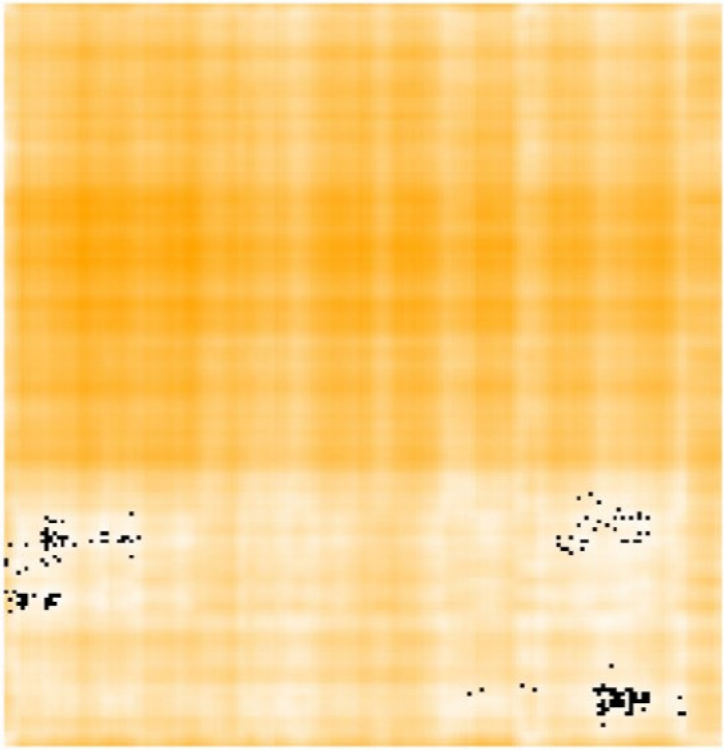

Figure S71 GM12878 Cell 12 Paternal Chr4 and Paternal Chr5

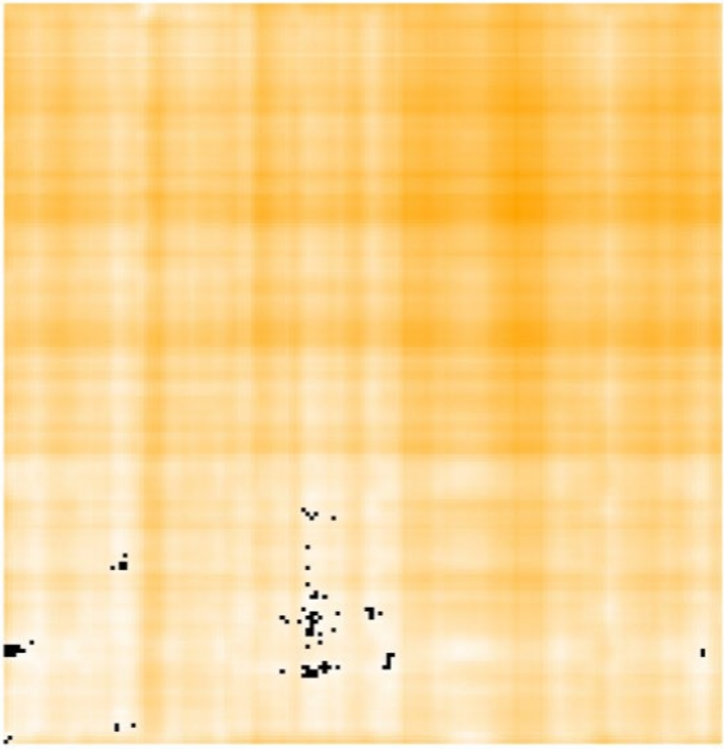

Figure S72 GM12878 Cell 13 Maternal Chr5 and Maternal Chr6

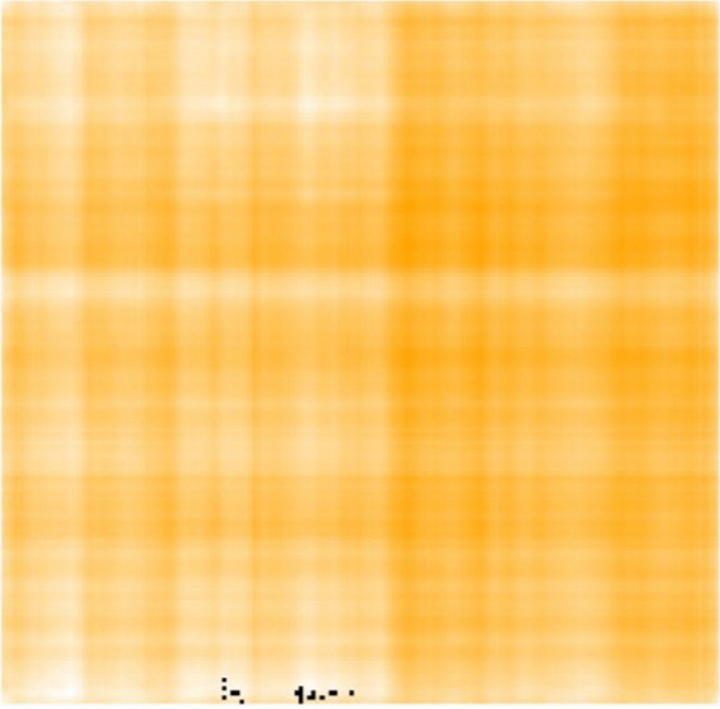

Figure S73 GM12878 Cell 14 Paternal Chr5 and Paternal Chr6

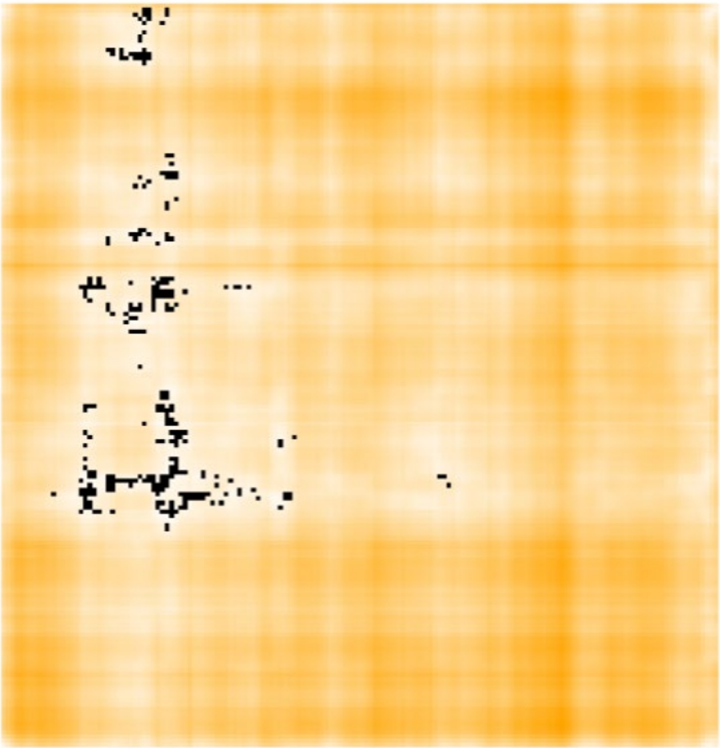

Figure S74 GM12878 Cell 15 Paternal Chr6 and Maternal Chr7

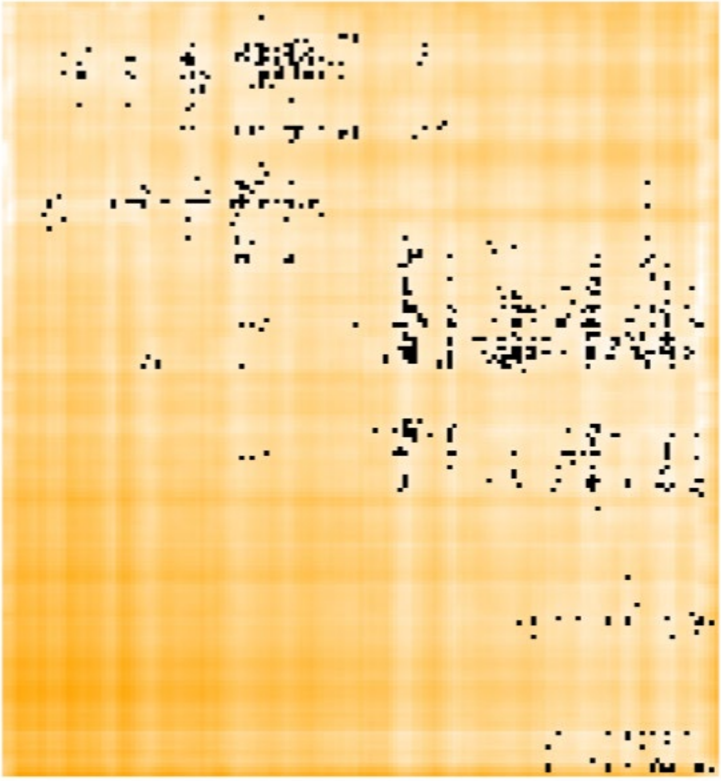

Figure S75 GM12878 Cell 16 Paternal Chr6 and Maternal Chr8

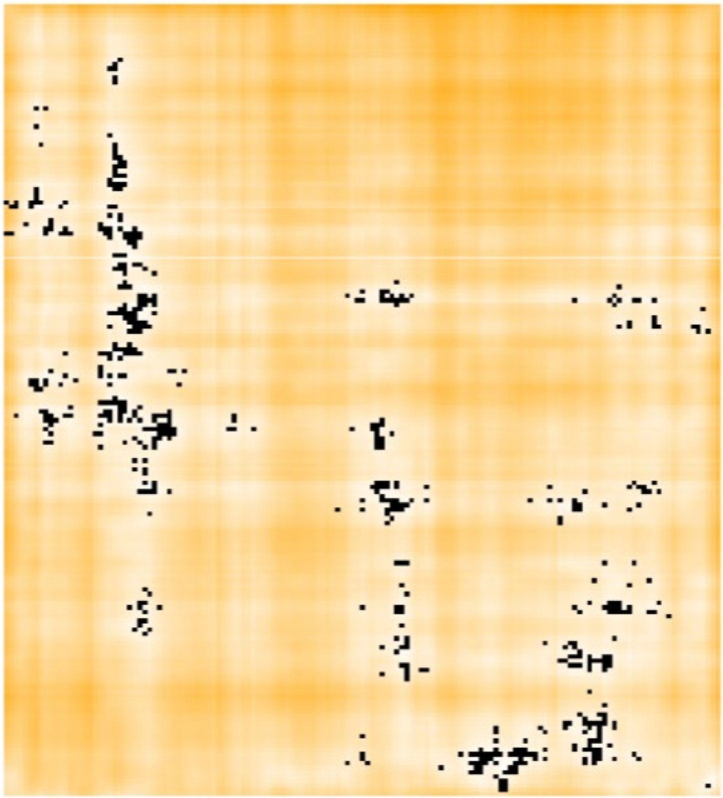

Figure S76 GM12878 Cell 17 Maternal Chr5 and Paternal Chr8

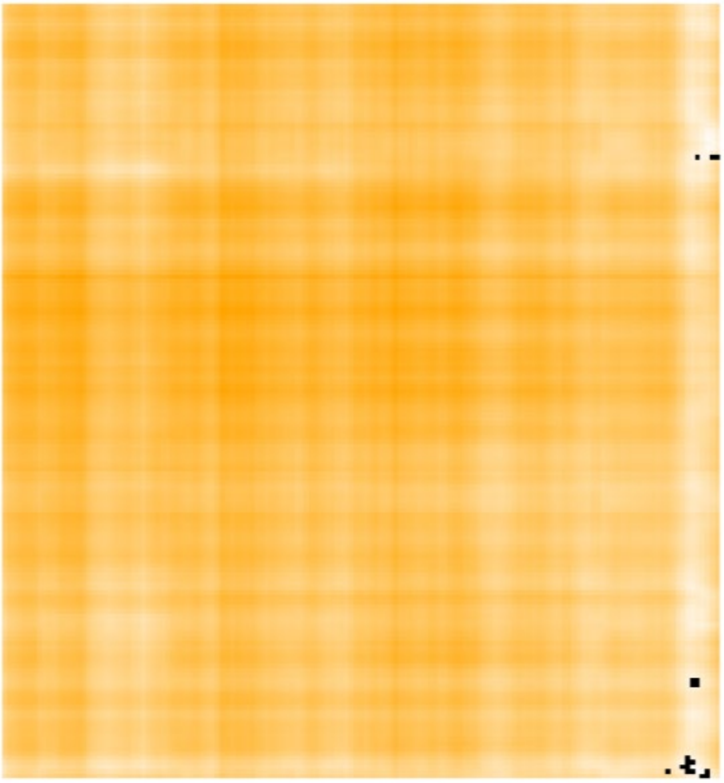

Figure S77 PBMC Cell 1 Paternal Chr6 and Paternal Chr8

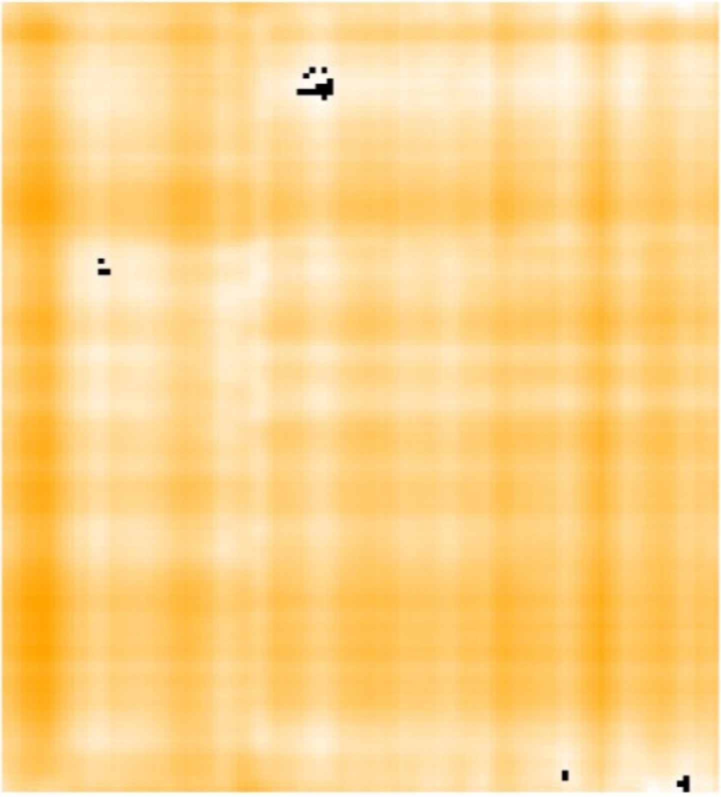

Figure S78 PBMC Cell 2 Maternal Chr8 and Paternal Chr9

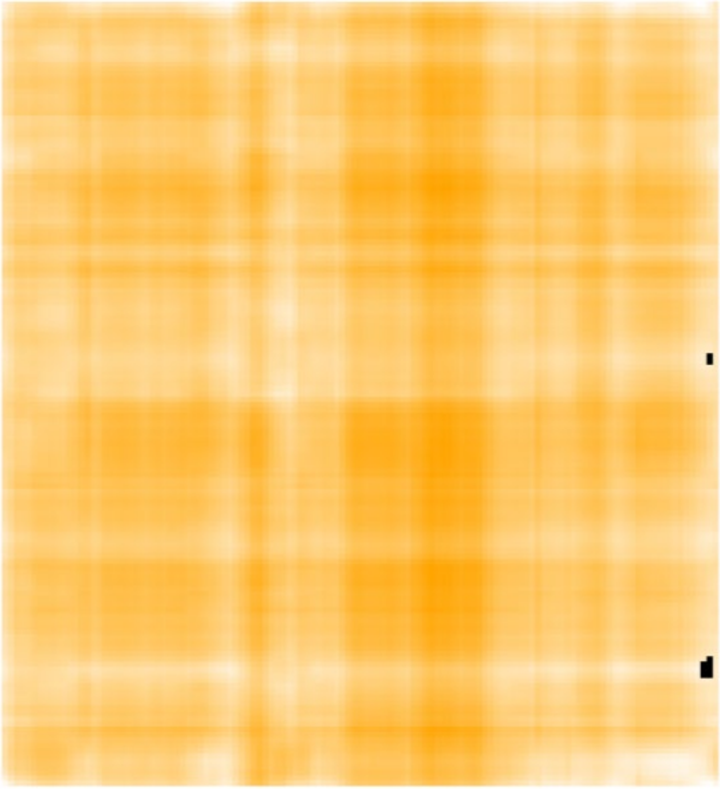

Figure S79 PBMC Cell 3 Paternal Chr8 and Maternal Chr9

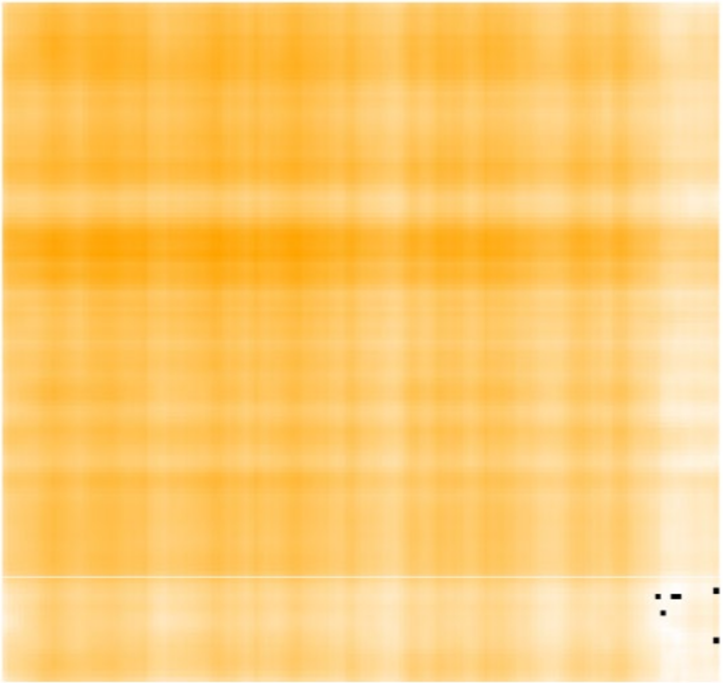

Figure S80 PBMC Cell 4 Maternal Chr9 and Paternal Chr10

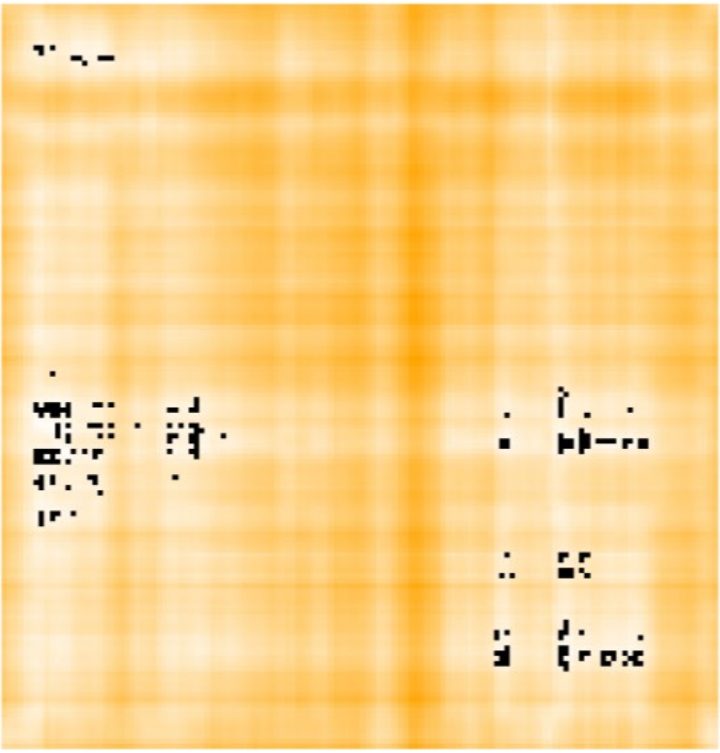

Figure S81 PBMC Cell 5 Paternal Chr8 and Paternal Chr10

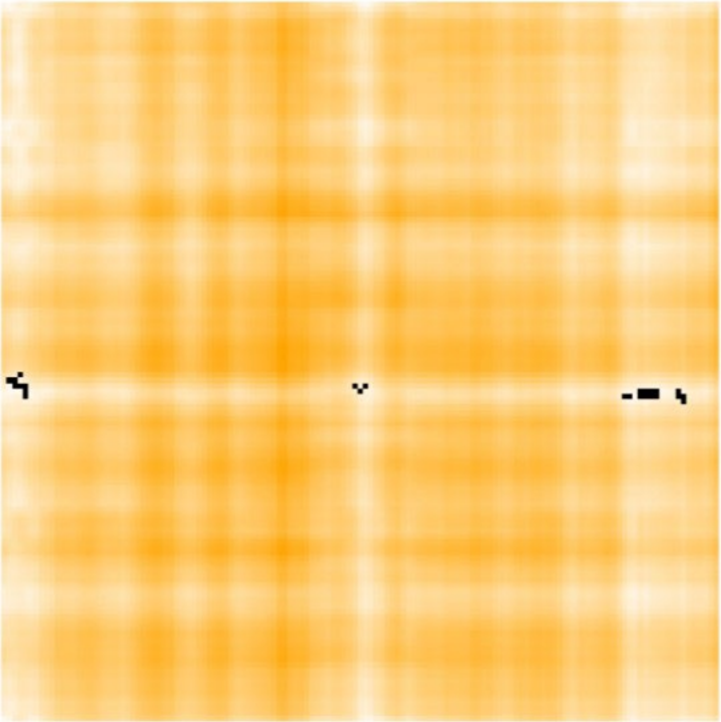

Figure S82 PBMC Cell 6 Paternal Chr10 and Paternal Chr11

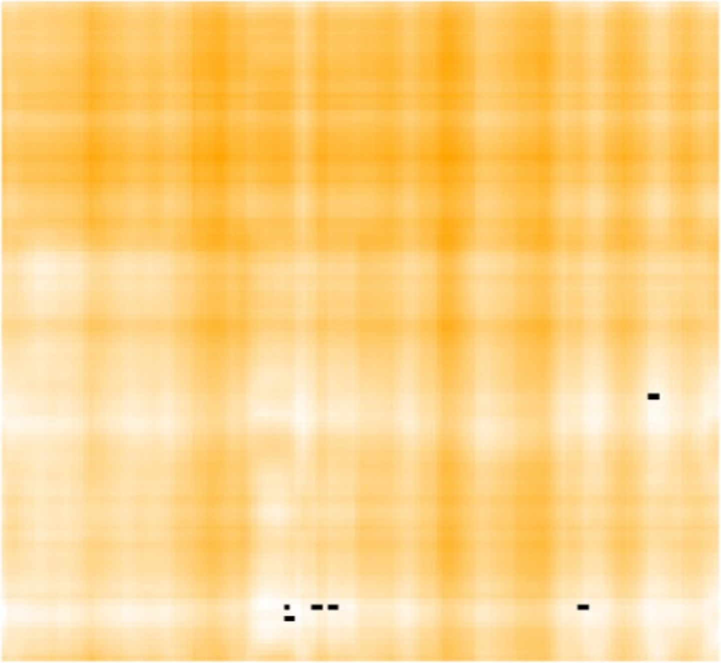

Figure S83 PBMC Cell 7 Maternal Chr9 and Paternal Chr12

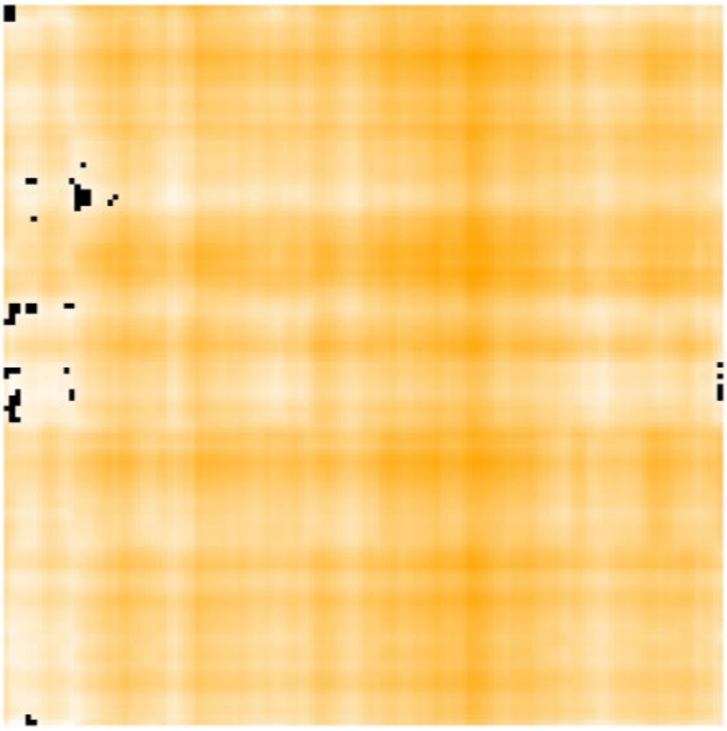

Figure S84 PBMC Cell 8 Paternal Chr11 and Paternal Chr12

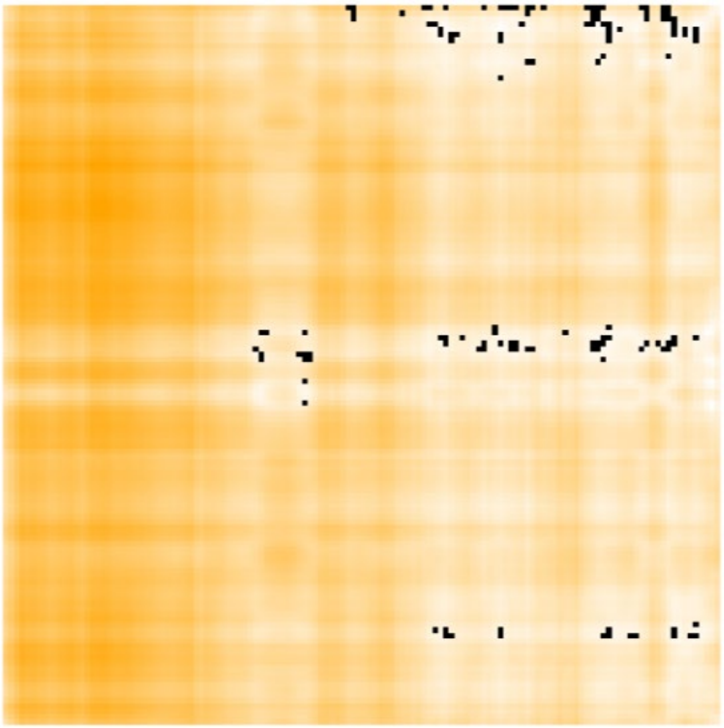

Figure S85 PBMC Cell 9 Paternal Chr11 and Paternal Chr12

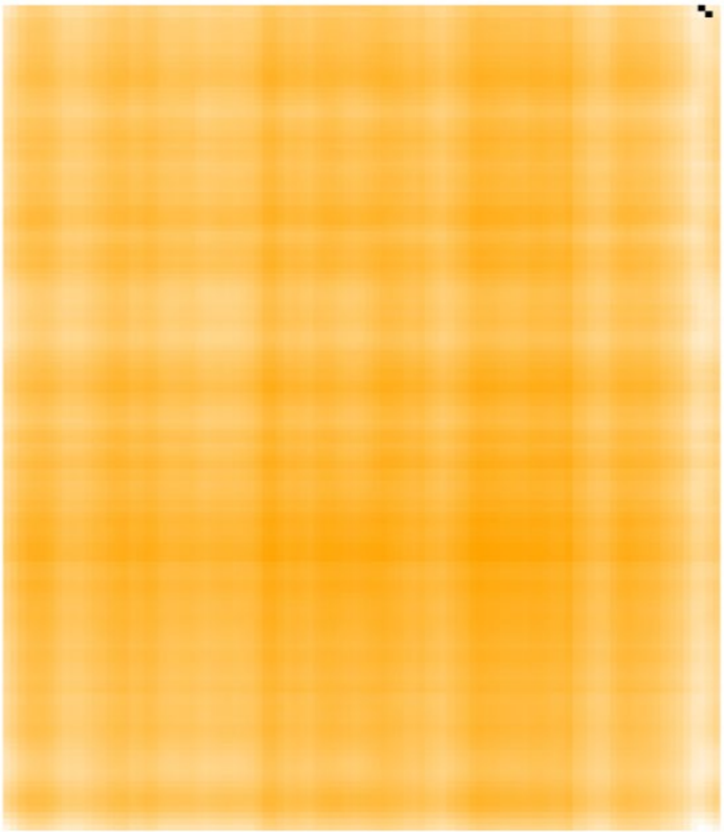

Figure S86 PBMC Cell 10 Paternal Chr12 and Maternal Chr13

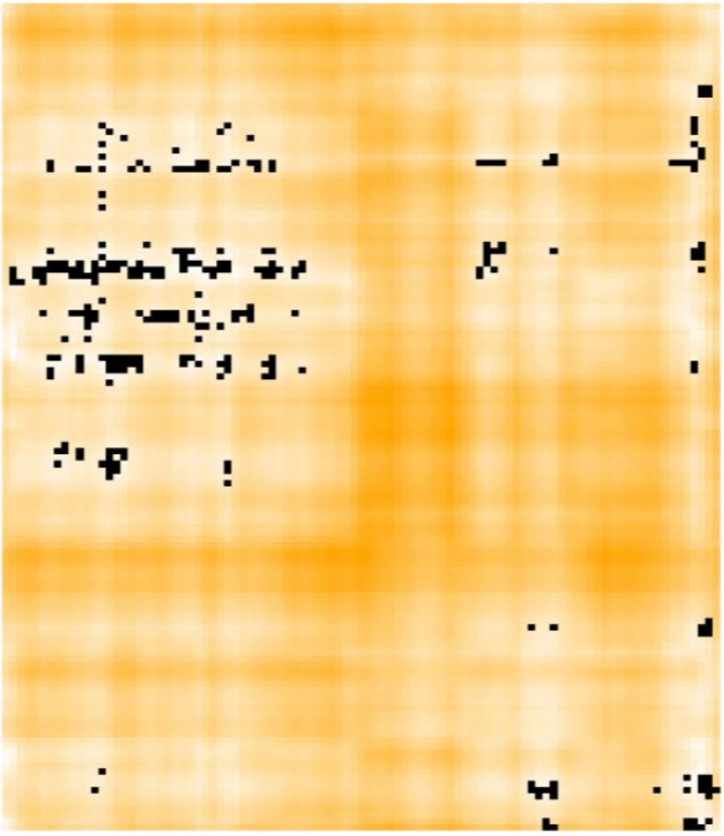

Figure S87 PBMC Cell 11 Maternal Chr12 and Maternal Chr13

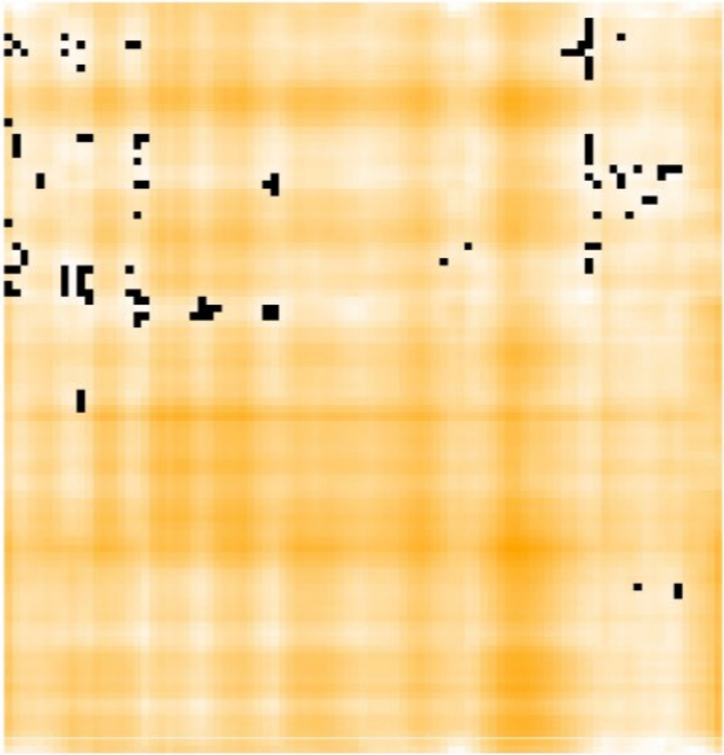

Figure S88 PBMC Cell 12 Paternal Chr13 and Paternal Chr14

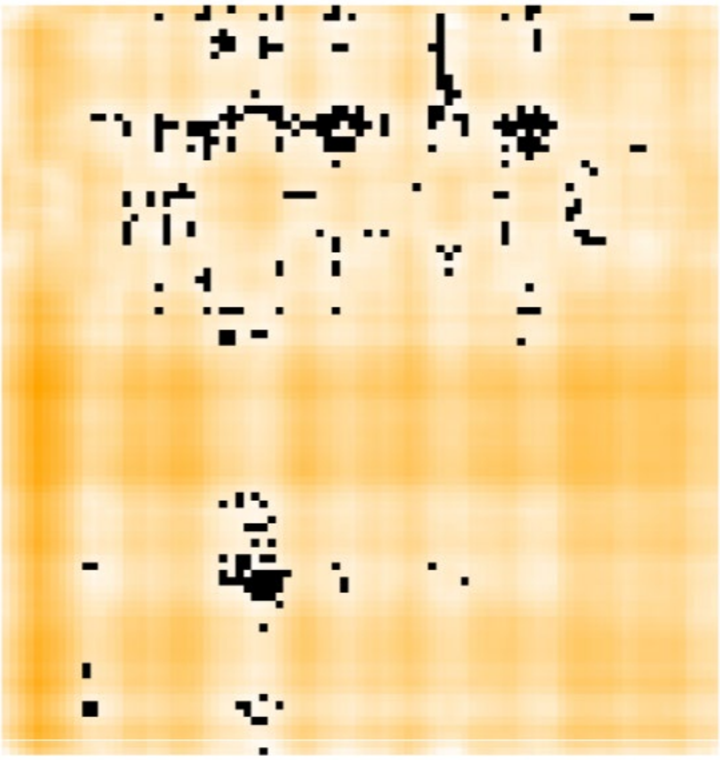

Figure S89 PBMC Cell 13 Paternal Chr13 and Maternal Chr14

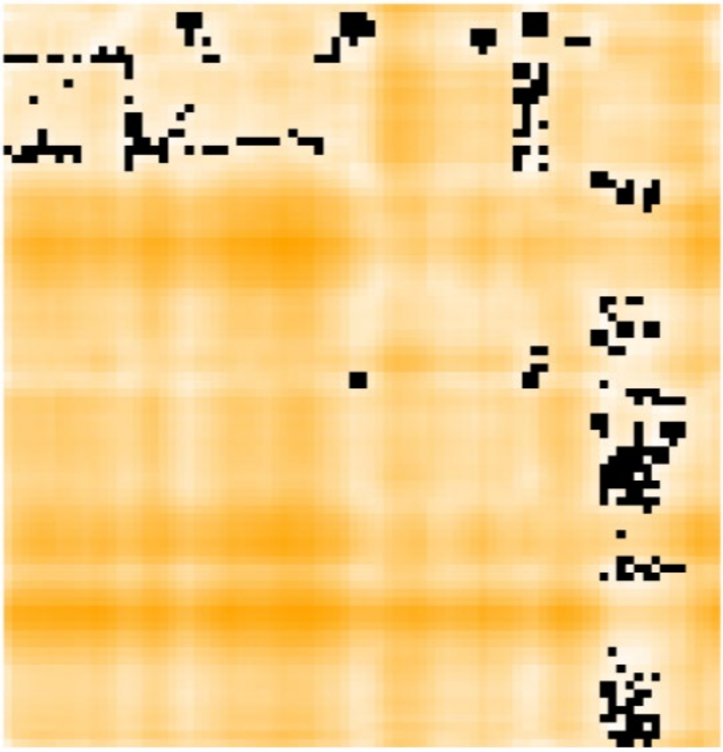

Figure S90 PBMC Cell 14 Paternal Chr14 and Maternal Chr15

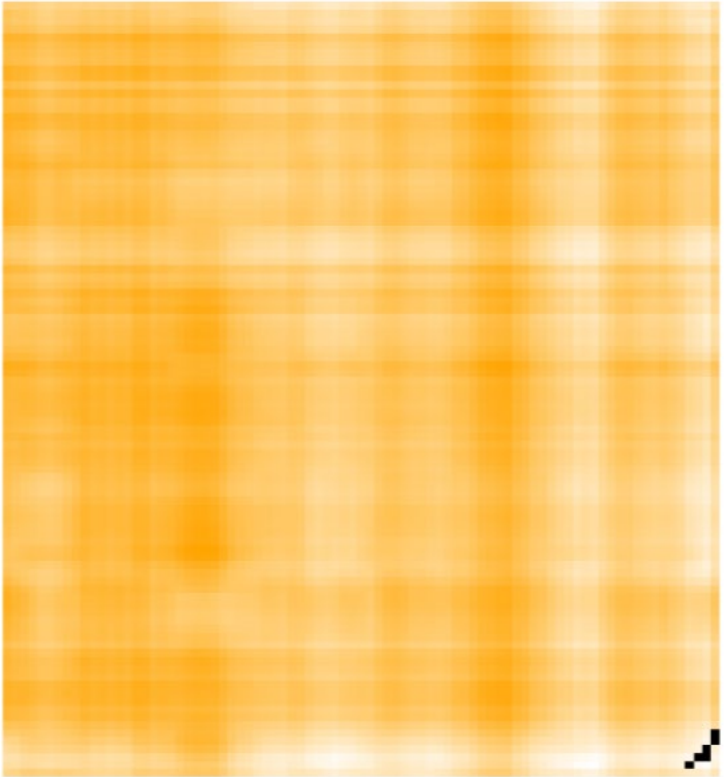

Figure S91 PBMC Cell 15 Paternal Chr13 and Paternal Chr15

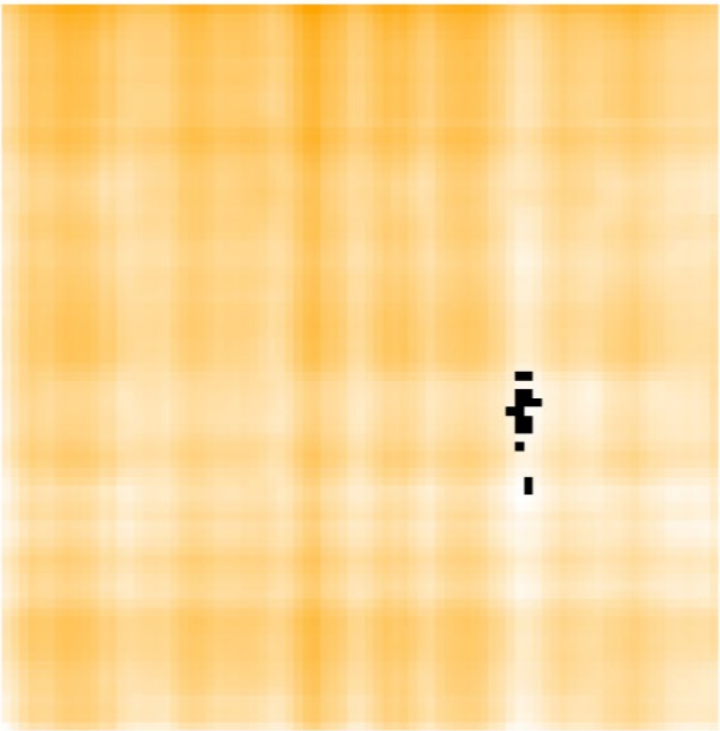

Figure S92 PBMC Cell 16 Paternal Chr15 and Maternal Chr16

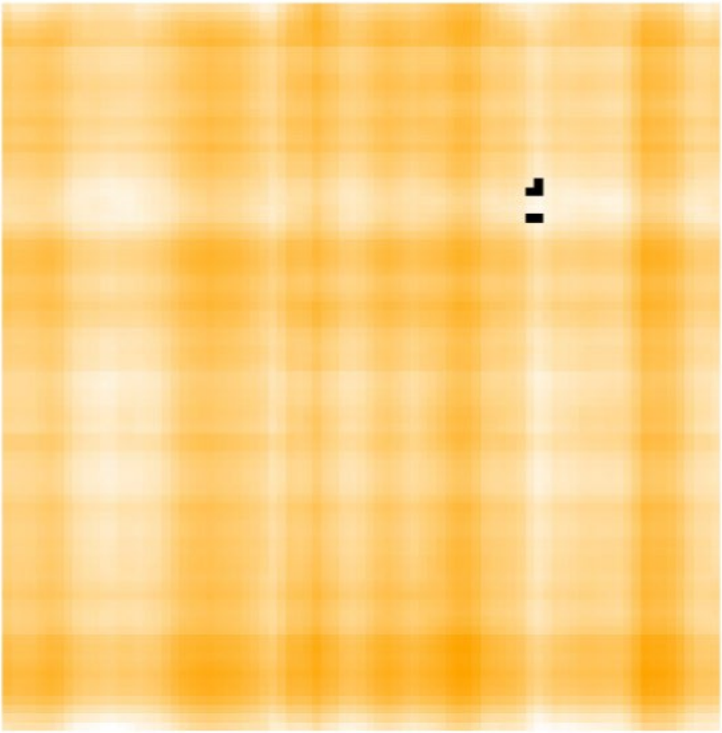

Figure S93 PBMC Cell 17 Maternal Chr15 and Maternal Chr16

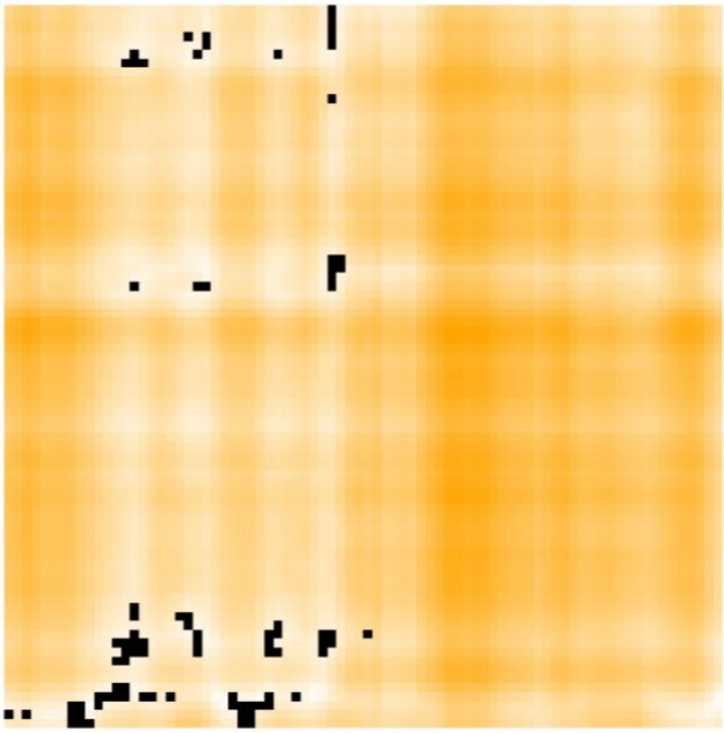

Figure S94 PBMC Cell 18 Paternal Chr16 and Paternal Chr17

Intra-chromosomal evaluations comparisons between SCW and Hickit, SCW and Nuc_dynamics, and SCW and Tensor-FLAMINGO.

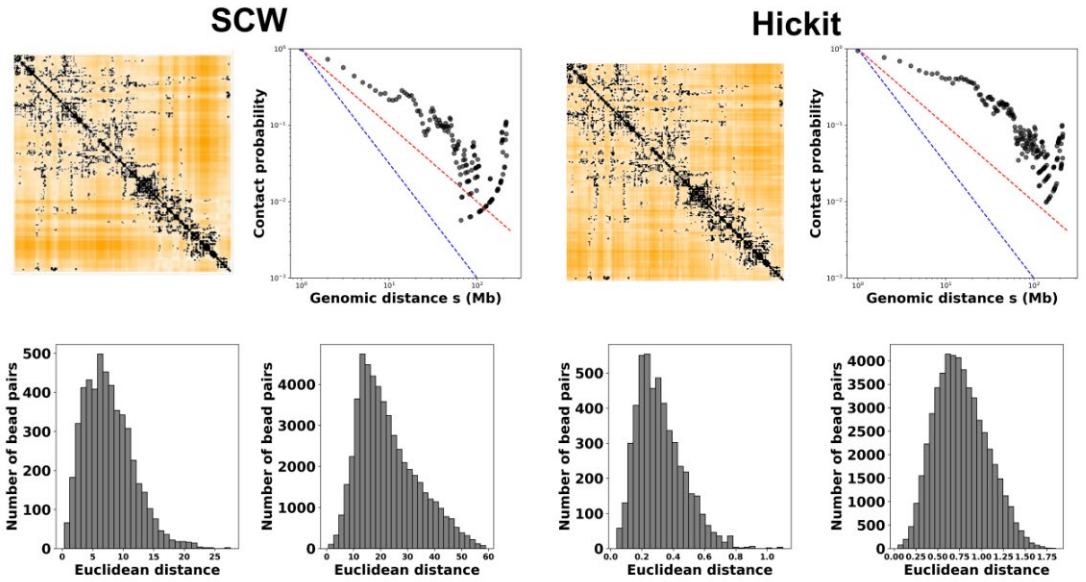

Figure S95 Intra-chromosomal evaluations for GM12878 Cell 5 Maternal Chr2 by using SCW and Hickit.

Notice: From Figures S95-S124, the left panel comprises four subfigures depicting the results from the SCW-based inference: (Top left) The overlay of Euclidean distances parsed from the SCW-inferred structures and the single-cell Hi-C contact matrix; (Top right) The relationship between contact probability and genomic distance in the corresponding Euclidean distance matrix; (Bottom, from left to right) The distributions of Euclidean distances for bead pairs with and without single-cell Hi-C contacts. The right panel arranges the same set of four analytical plots in an identical layout, but generated from the 3D structures inferred by the other tool.

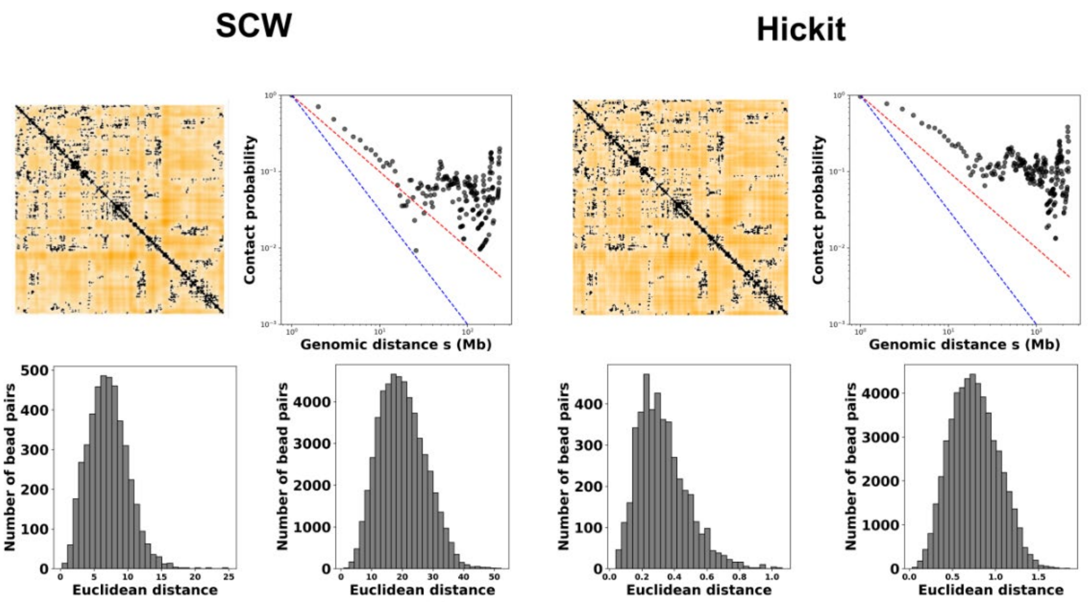

Figure S96 Intra-chromosomal evaluations for GM12878 Cell 6 Paternal Chr2 by using SCW and Hickit

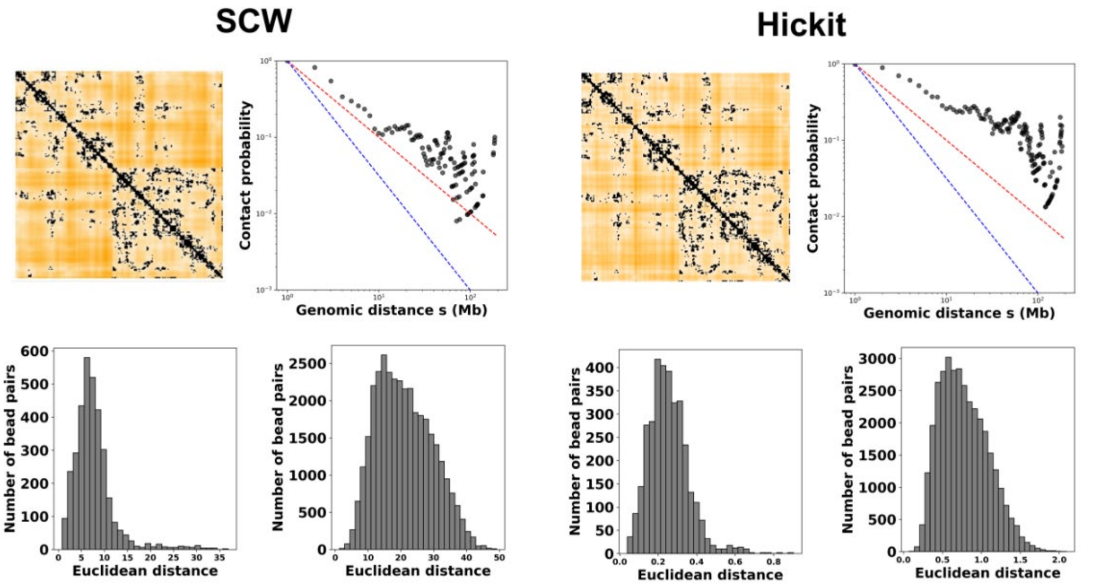

Figure S97 Intra-chromosomal evaluations for GM12878 Cell 7 Maternal Chr3 by using SCW and Hickit

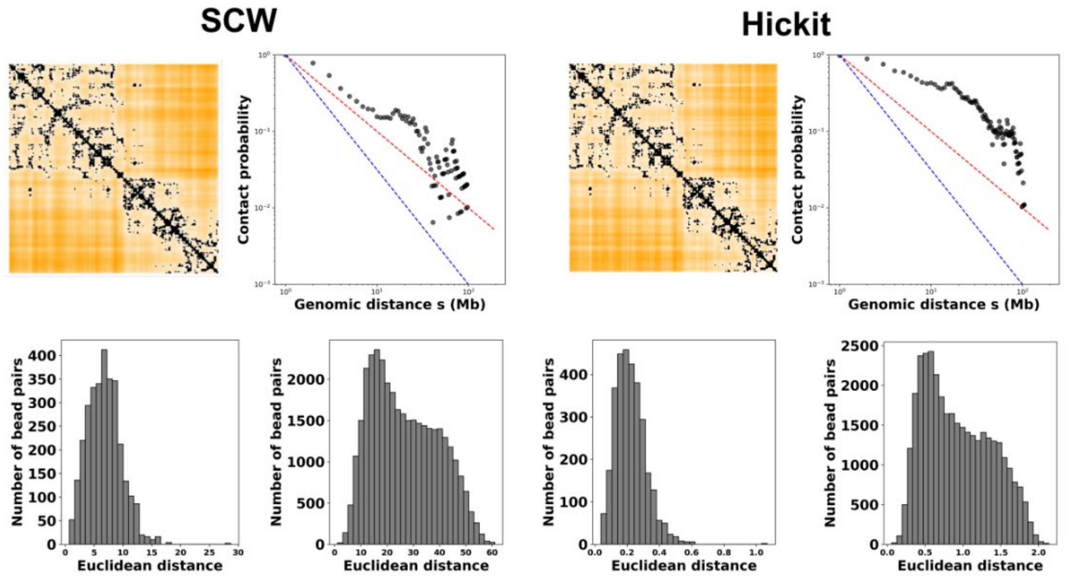

Figure S98 Intra-chromosomal evaluations for GM12878 Cell 9 Paternal Chr3 by using SCW and Hickit

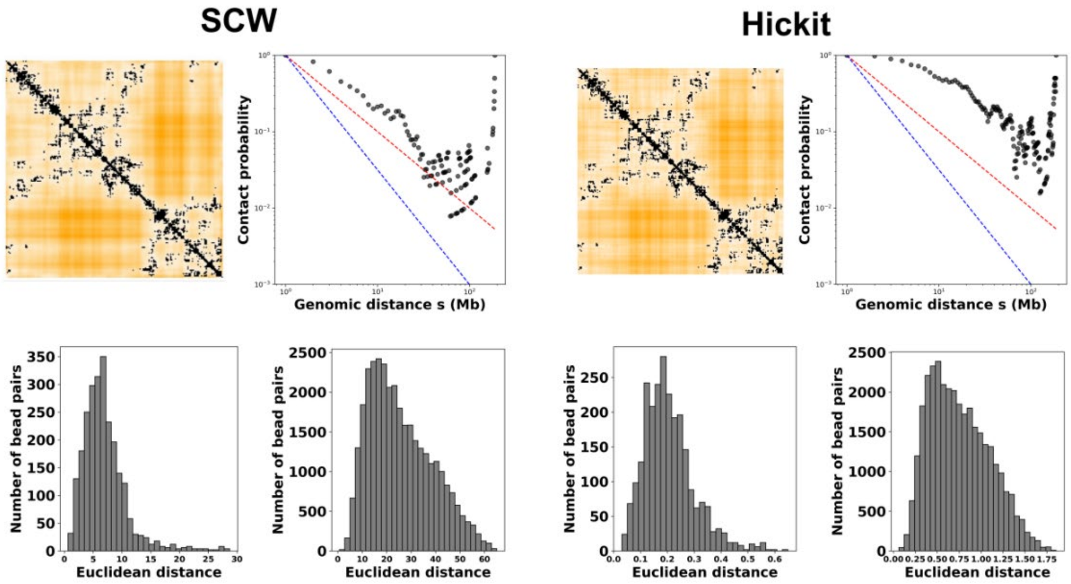

Figure S99 Intra-chromosomal evaluations for GM12878 Cell 12 Paternal Chr4 by using SCW and Hickit

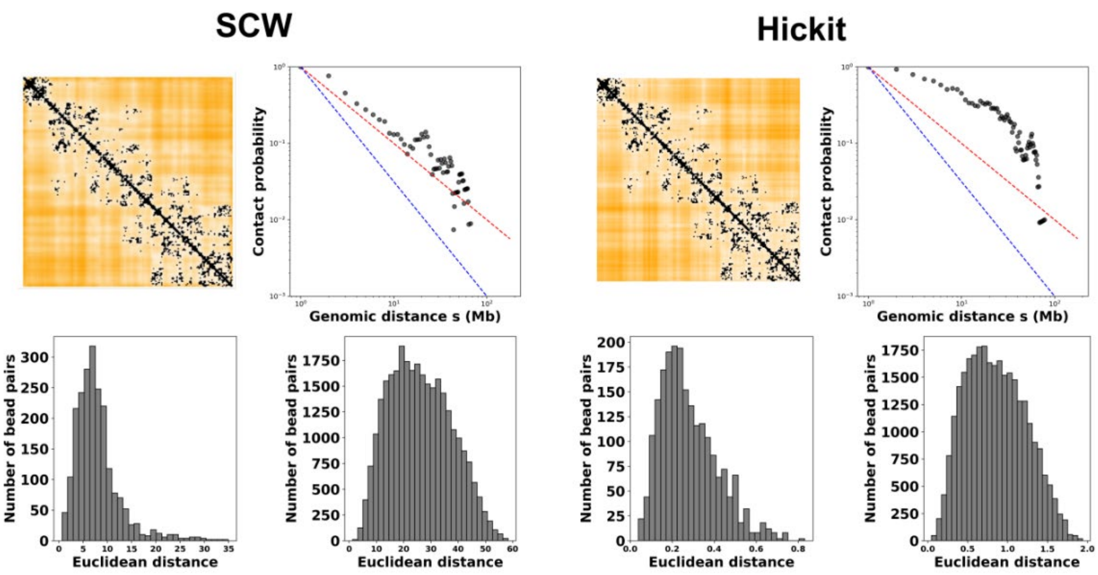

Figure S100 Intra-chromosomal evaluations for GM12878 Cell 13 Maternal Chr5 by using SCW and Hickit

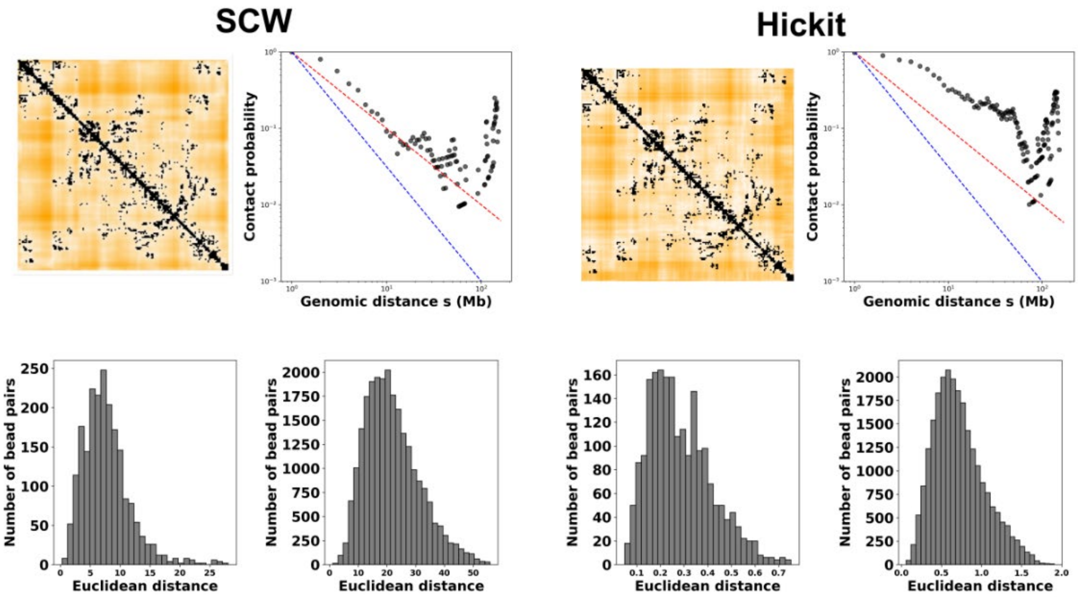

Figure S101 Intra-chromosomal evaluations for GM12878 Cell 14 Paternal Chr5 by using SCW and Hickit

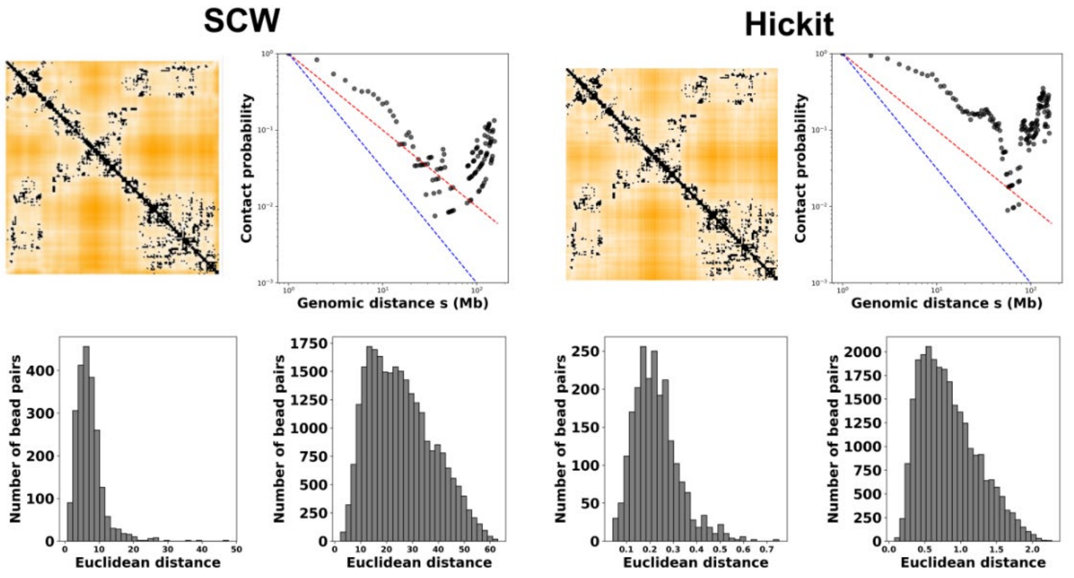

Figure S102 Intra-chromosomal evaluations for GM12878 Cell 15 Maternal Chr6 by using SCW and Hickit

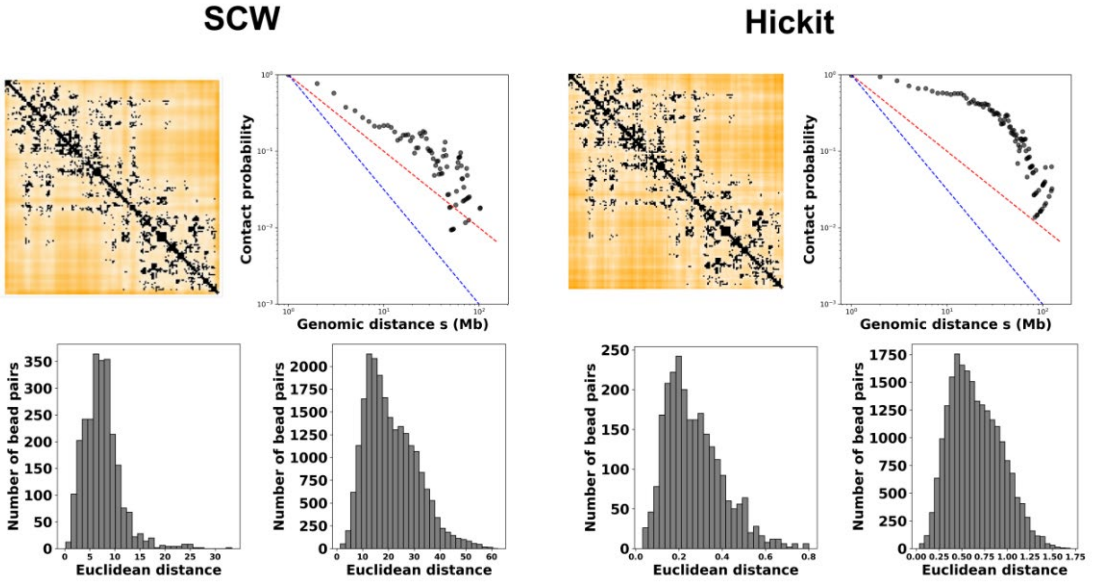

Figure S103 Intra-chromosomal evaluations for GM12878 Cell 17 Maternal Chr7 by using SCW and Hickit

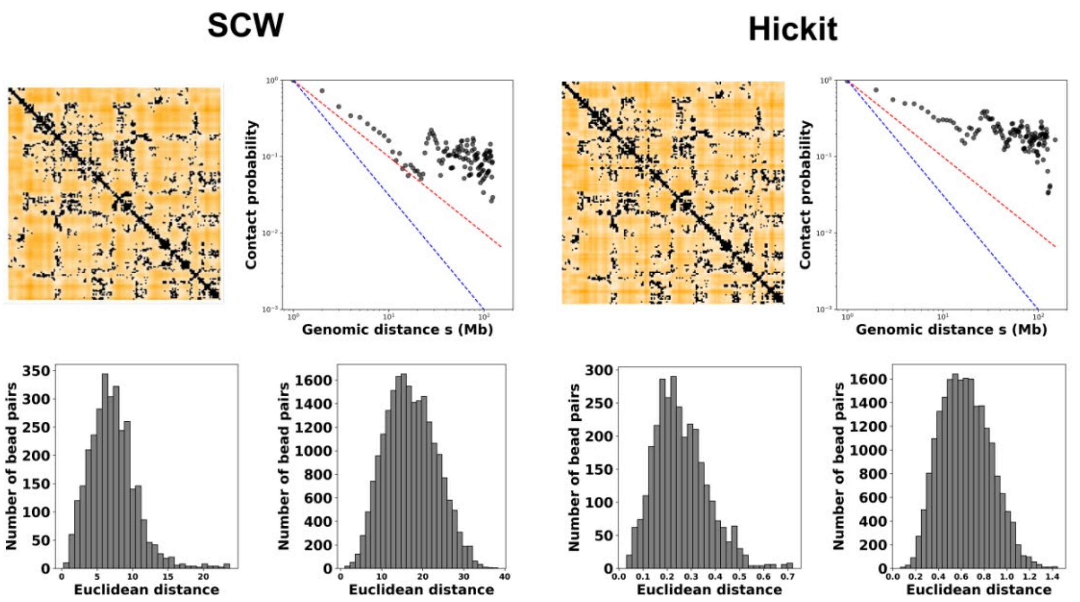

Figure S104 Intra-chromosomal evaluations for PBMC Cell 1 Paternal Chr7 by using SCW and Hickit

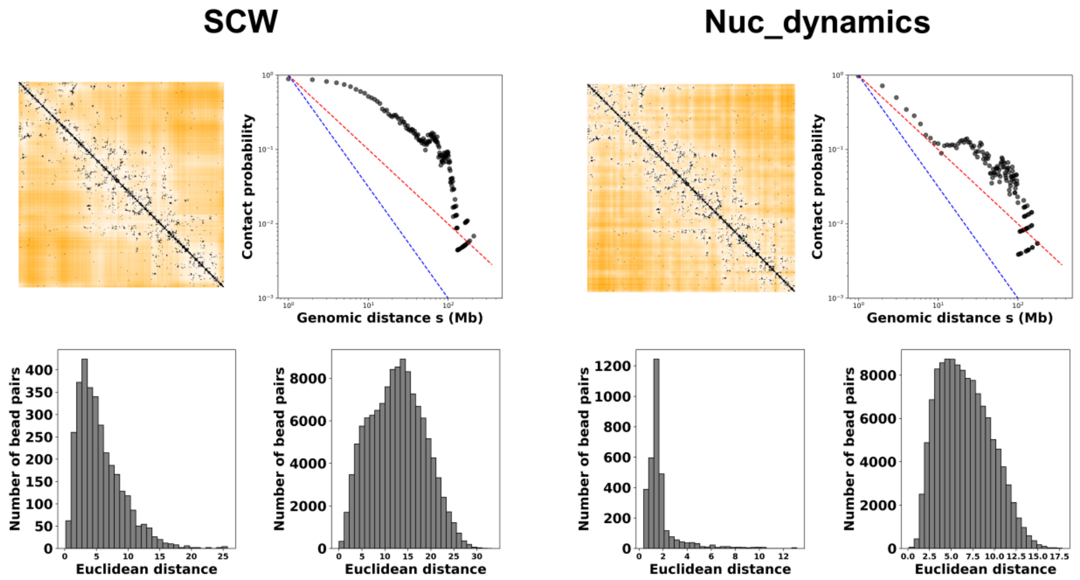

Figure S105 Intra-chromosomal evaluations for TH1 Cell1 Chr2 by using SCW and Nuc_dynamics

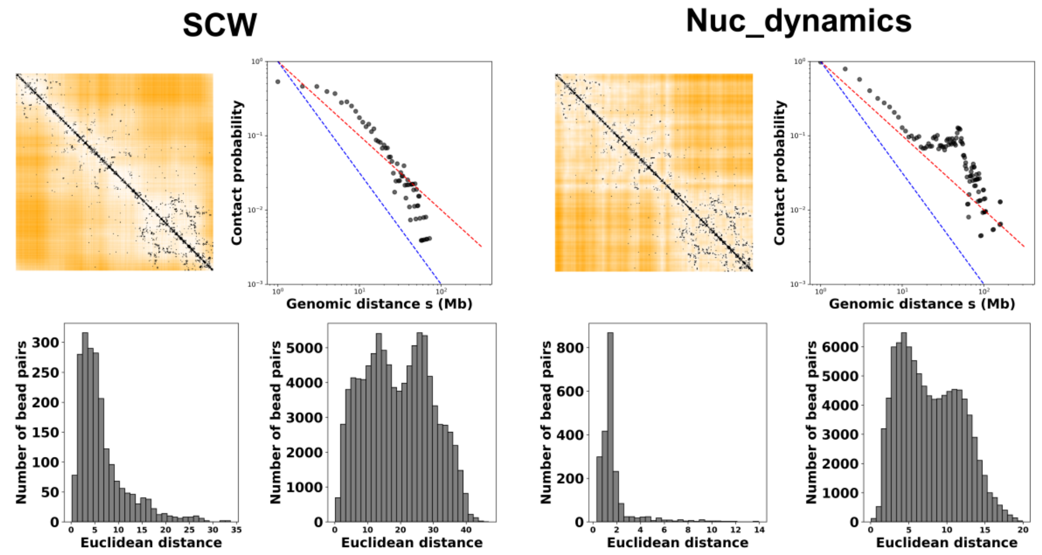

Figure S106 Intra-chromosomal evaluations for TH1 Cell1 Chr3 by using SCW and Nuc_dynamics

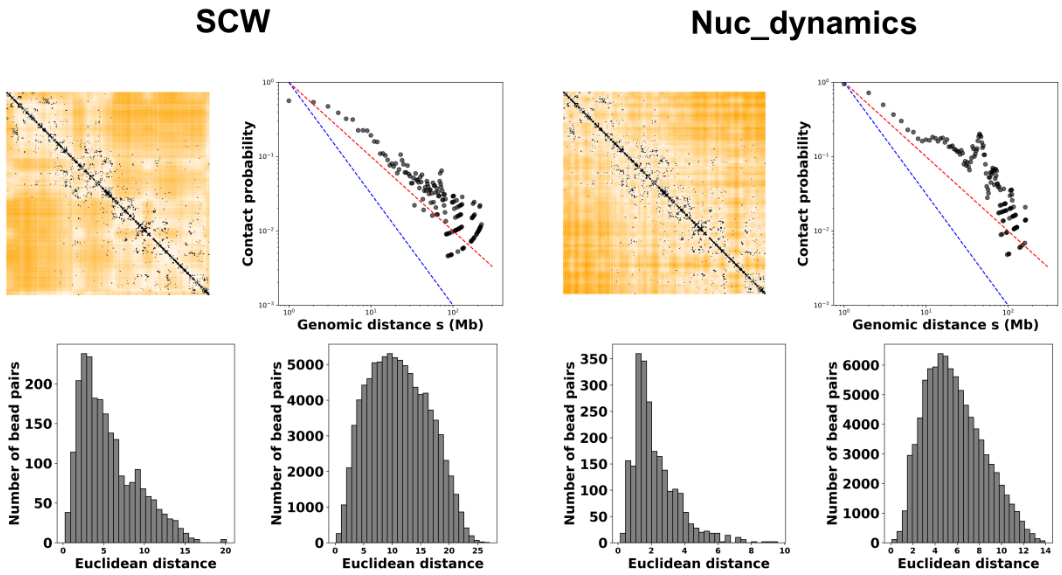

Figure S107 Intra-chromosomal evaluations for TH1 Cell1 Chr4 by using SCW and Nuc_dynamics

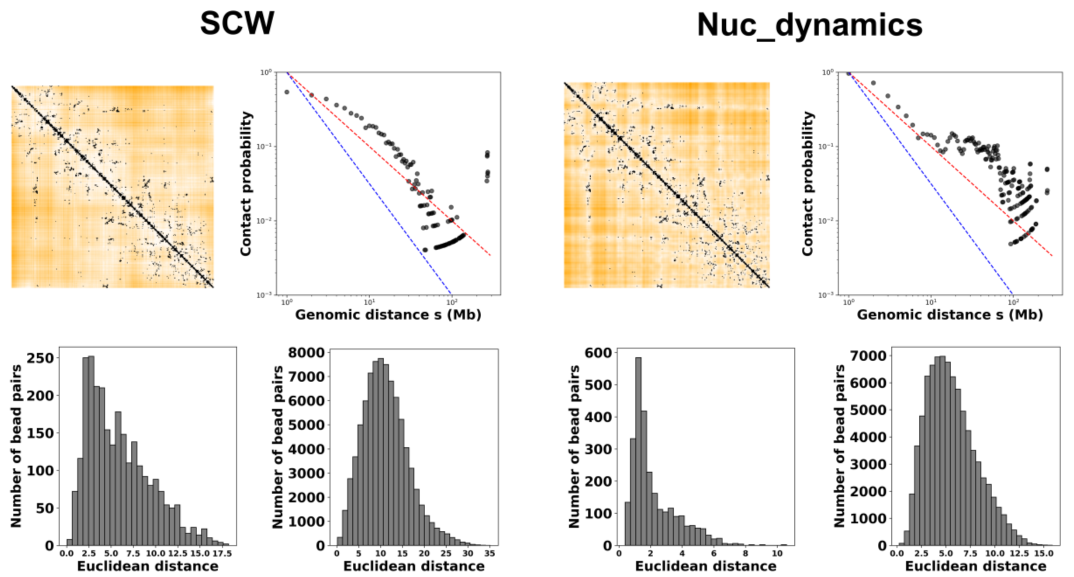

Figure S108 Intra-chromosomal evaluations for TH1 Cell1 Chr5 by using SCW and Nuc_dynamics

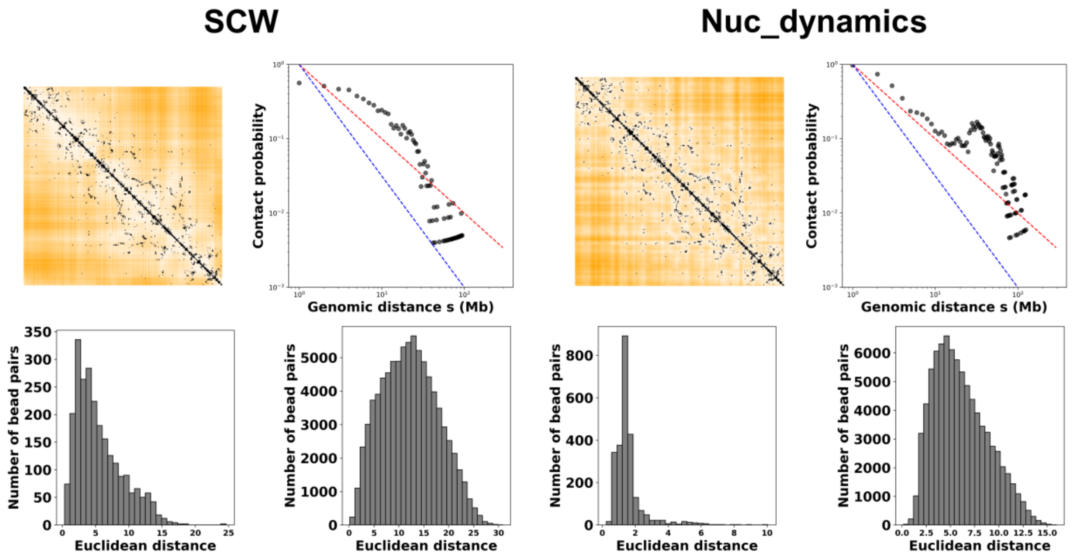

Figure S109 Intra-chromosomal evaluations for TH1 Cell1 Chr6 by using SCW and Nuc_dynamics

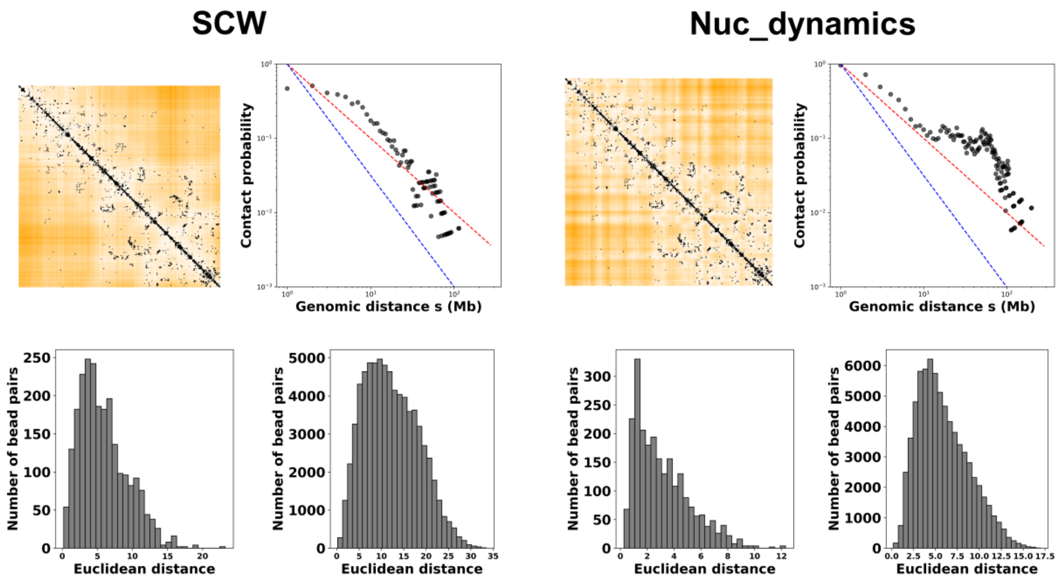

Figure S110 Intra-chromosomal evaluations for TH1 Cell1 Chr7 by using SCW and Nuc_dynamics

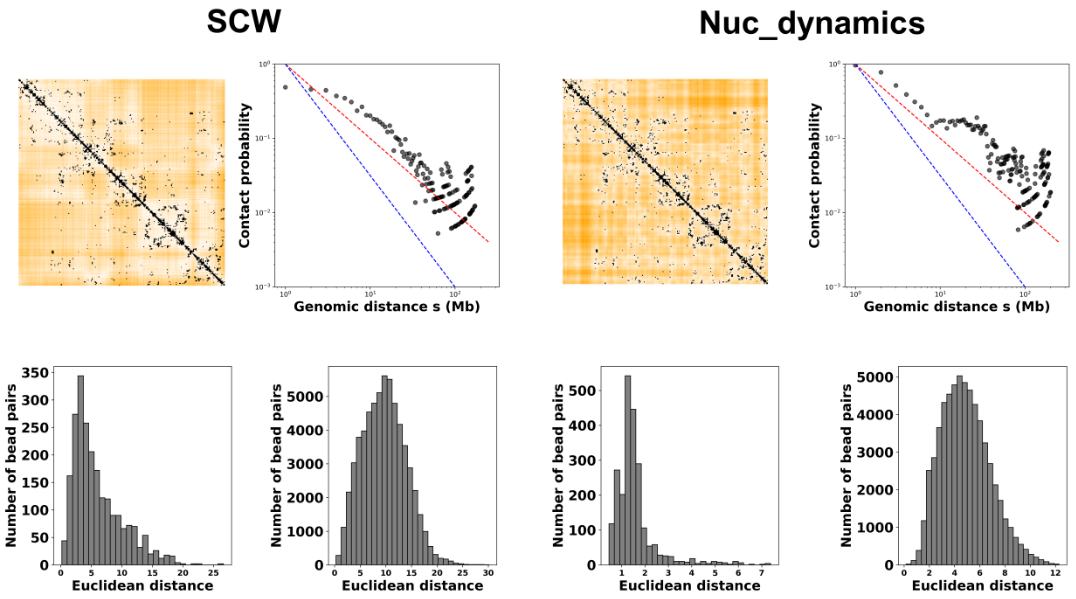

Figure S111 Intra-chromosomal evaluations for TH1 Cell1 Chr8 by using SCW and Nuc_dynamics

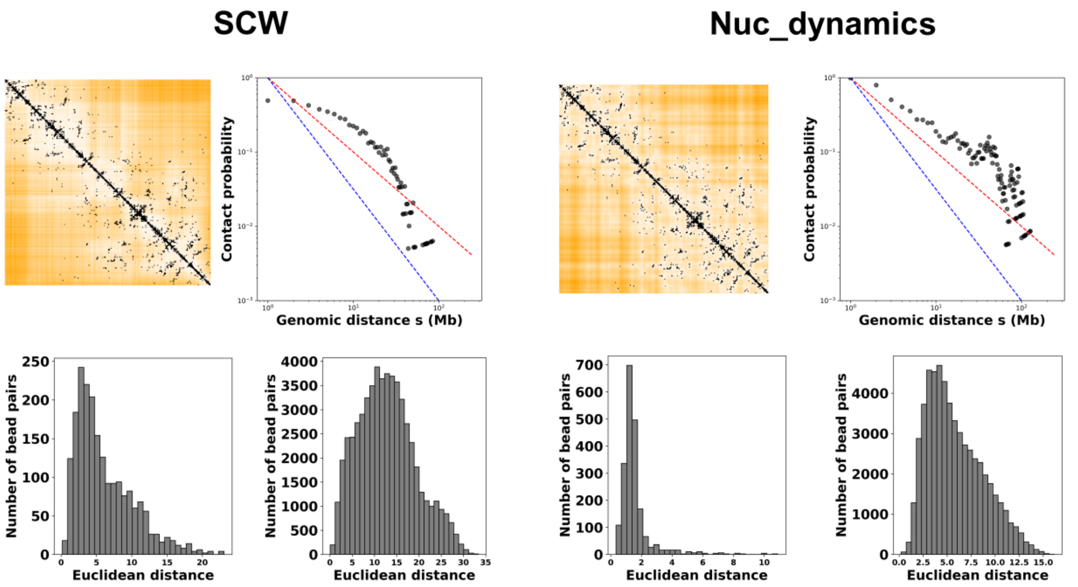

Figure S112 Intra-chromosomal evaluations for TH1 Cell1 Chr9 by using SCW and Nuc_dynamics

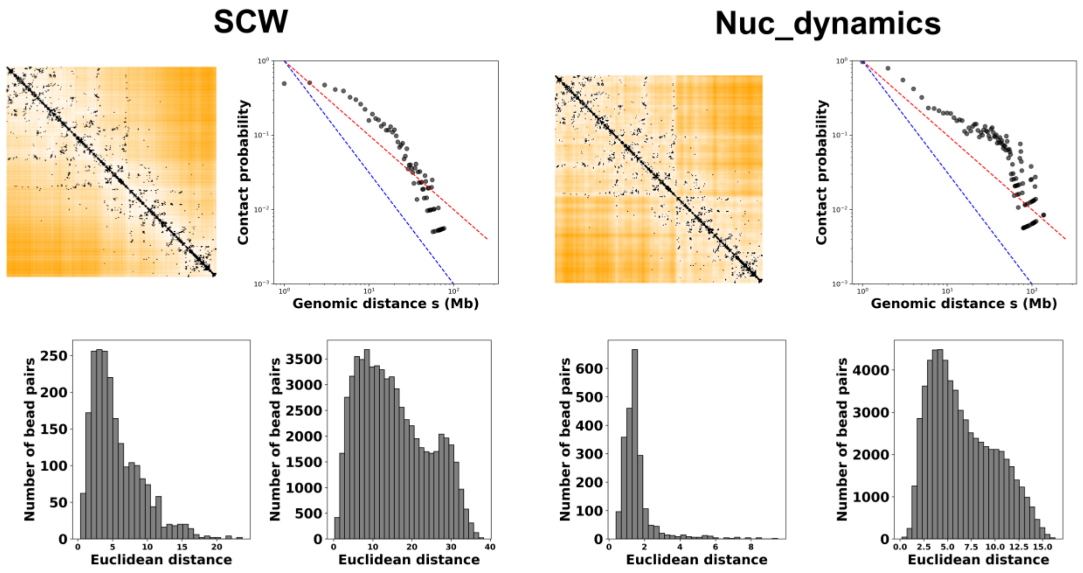

Figure S113 Intra-chromosomal evaluations for TH1 Cell1 Chr10 by using SCW and Nuc_dynamics

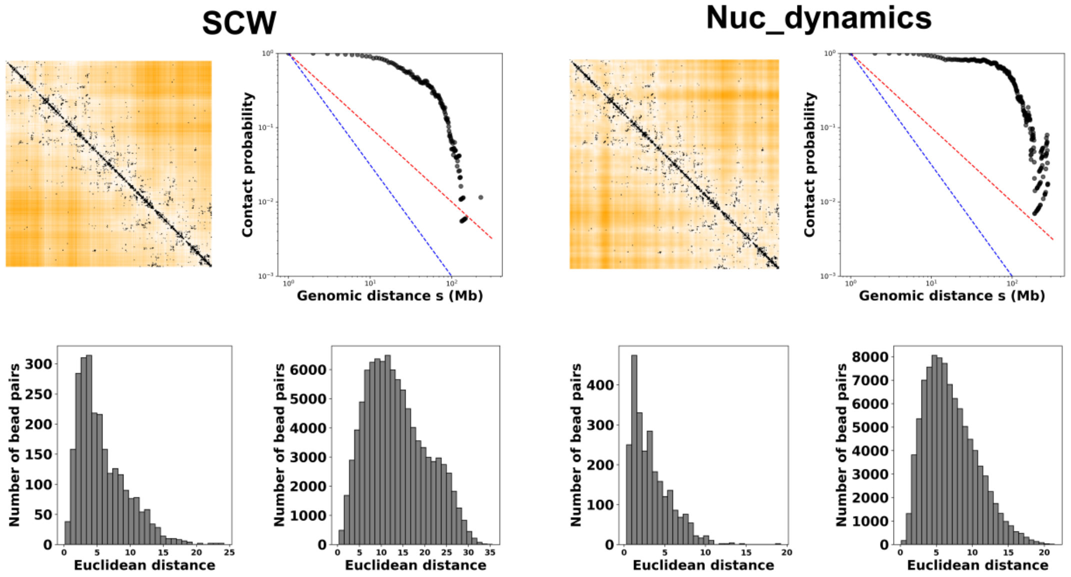

Figure S114 Intra-chromosomal evaluations for TH1 Cell1 ChrX by using SCW and Nuc_dynamics

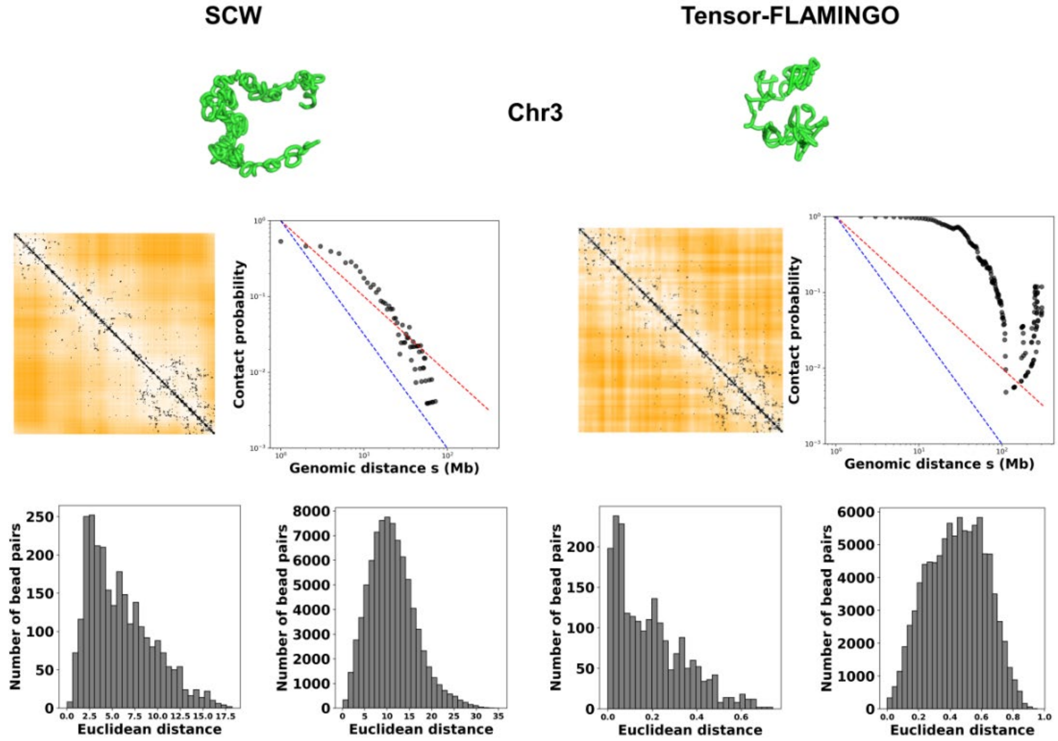

Figure S115 Intra-chromosomal structures and evaluations for TH1 Cell1 Chr3 by using SCW and Tensor-FLAMINGO

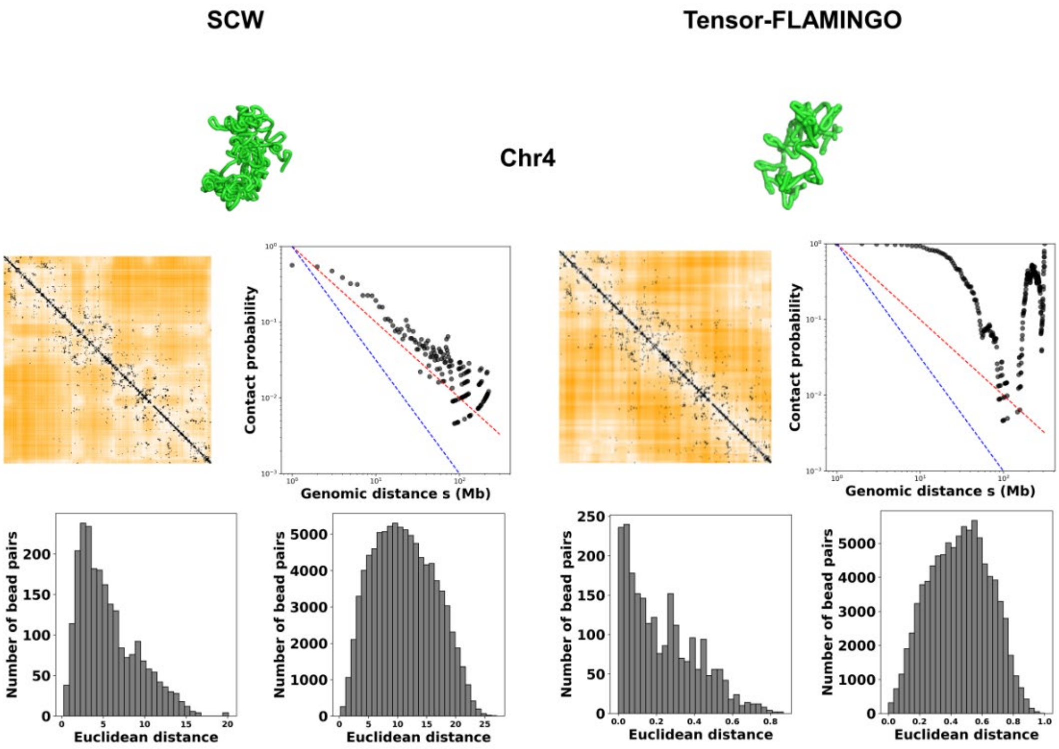

Figure S116 Intra-chromosomal structures and evaluations for TH1 Cell1 Chr4 by using SCW and Tensor-FLAMINGO

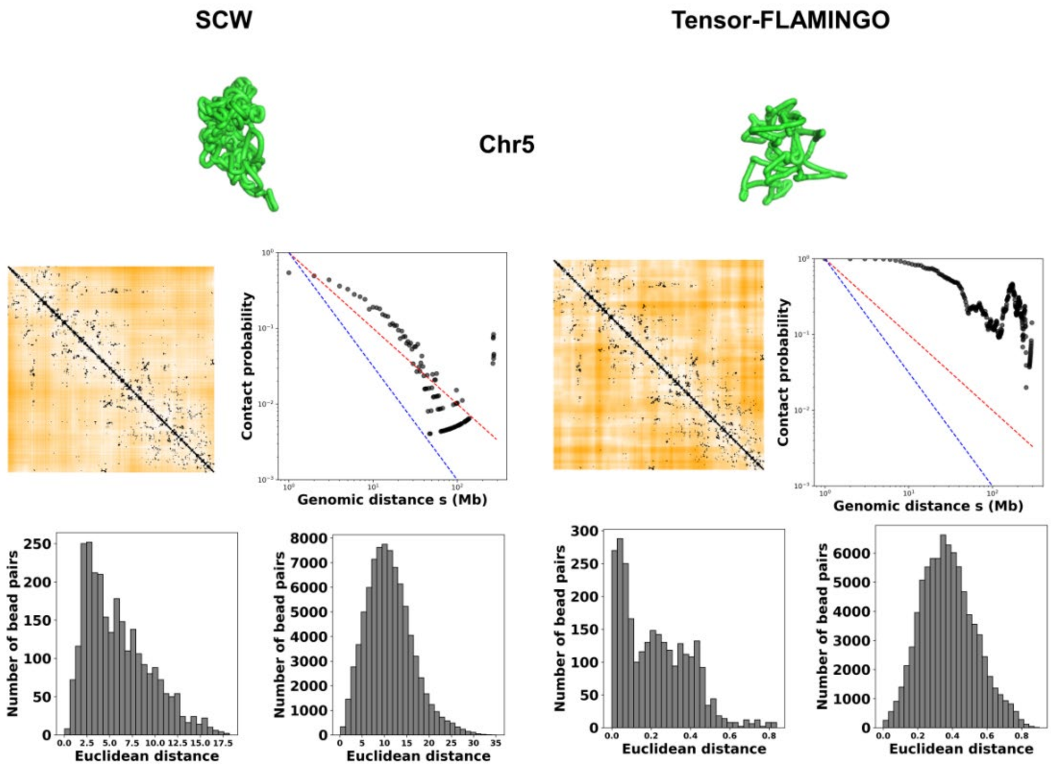

Figure S117 Intra-chromosomal structures and evaluations for TH1 Cell1 Chr5 by using SCW and Tensor-FLAMINGO

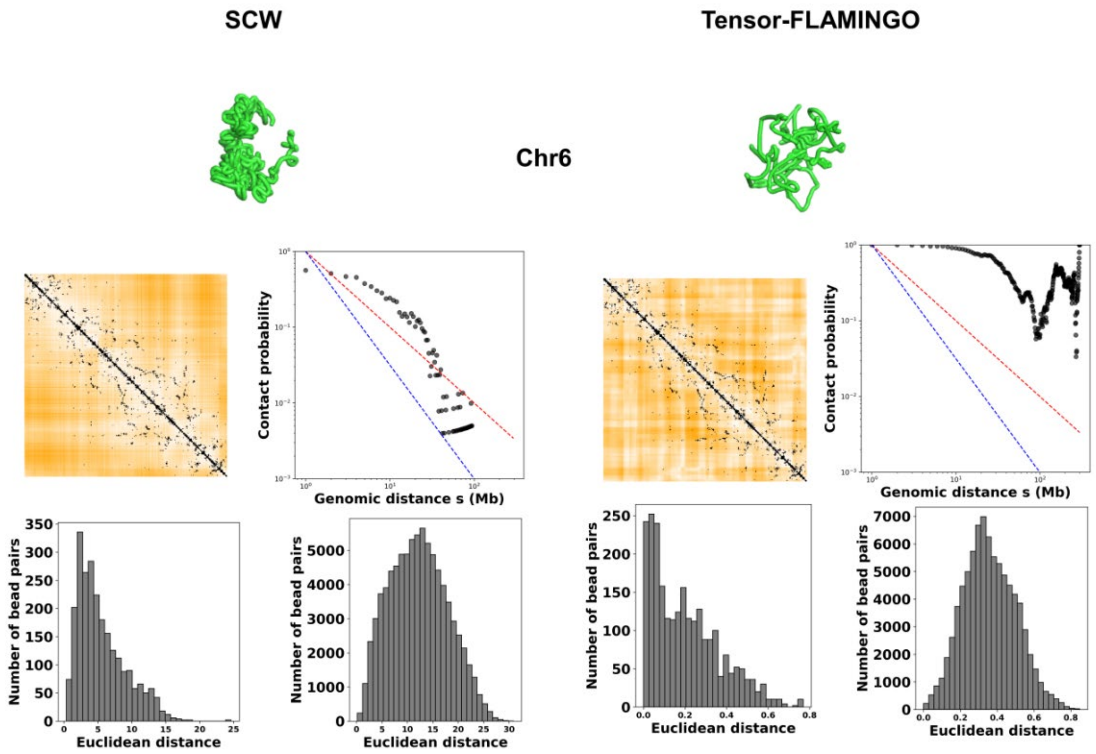

Figure S118 Intra-chromosomal structures and evaluations for TH1 Cell1 Chr6 by using SCW and Tensor-FLAMINGO

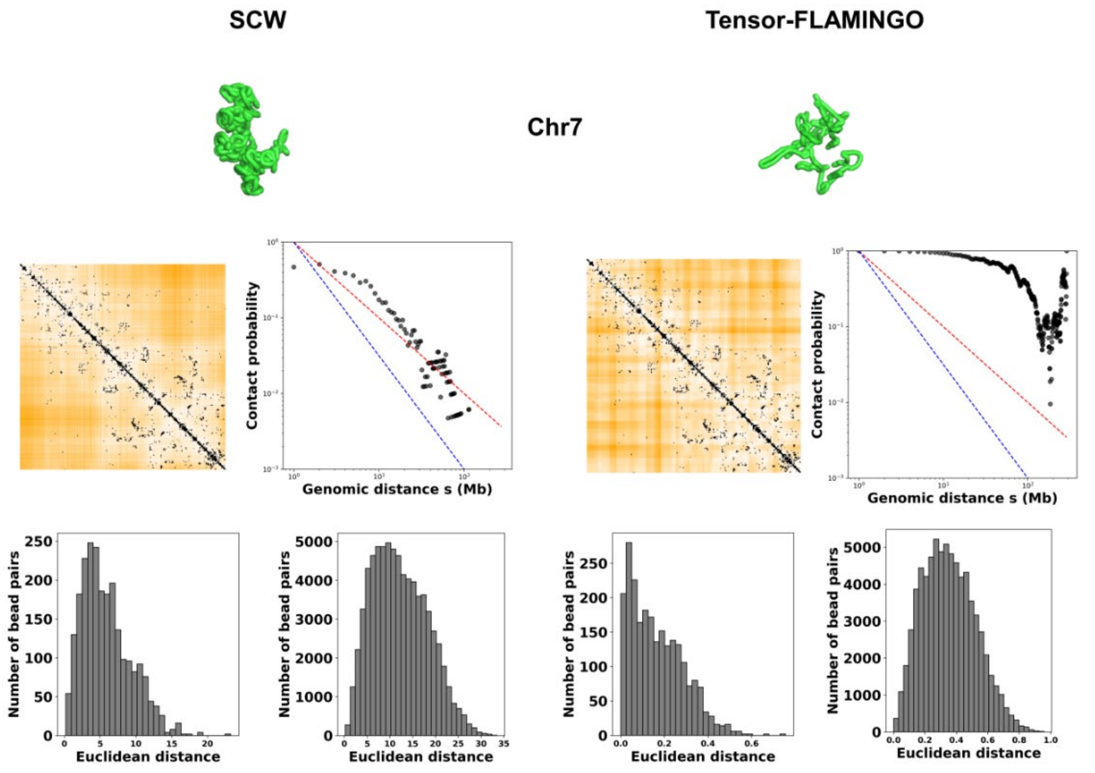

Figure S119 Intra-chromosomal structures and evaluations for TH1 Cell1 Chr7 by using SCW and Tensor-FLAMINGO

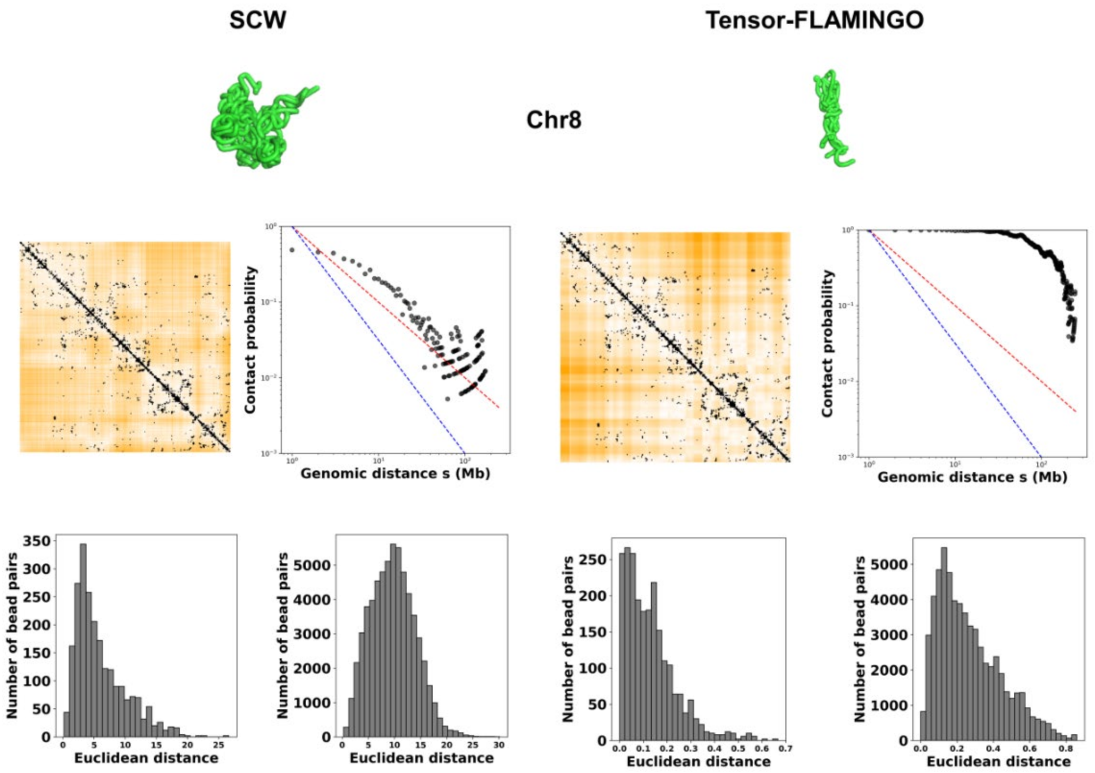

Figure S120 Intra-chromosomal structures and evaluations for TH1 Cell1 Chr8 by using SCW and Tensor-FLAMINGO

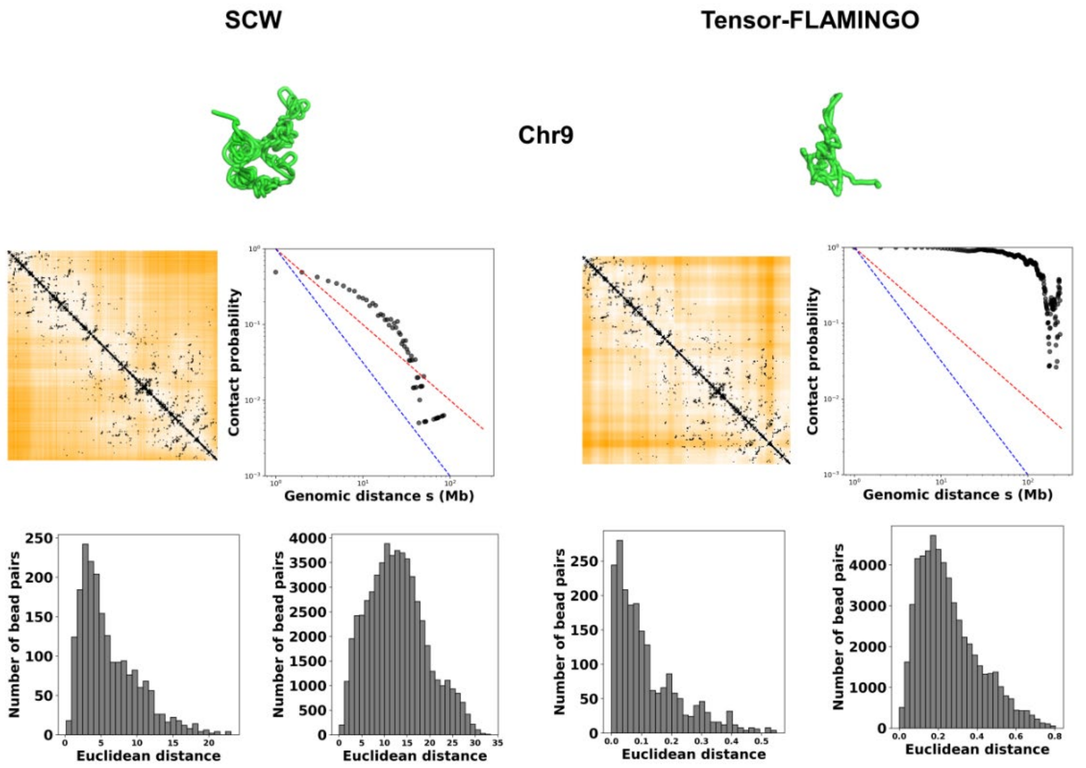

Figure S121 Intra-chromosomal structures and evaluations for TH1 Cell1 Chr9 by using SCW and Tensor-FLAMINGO

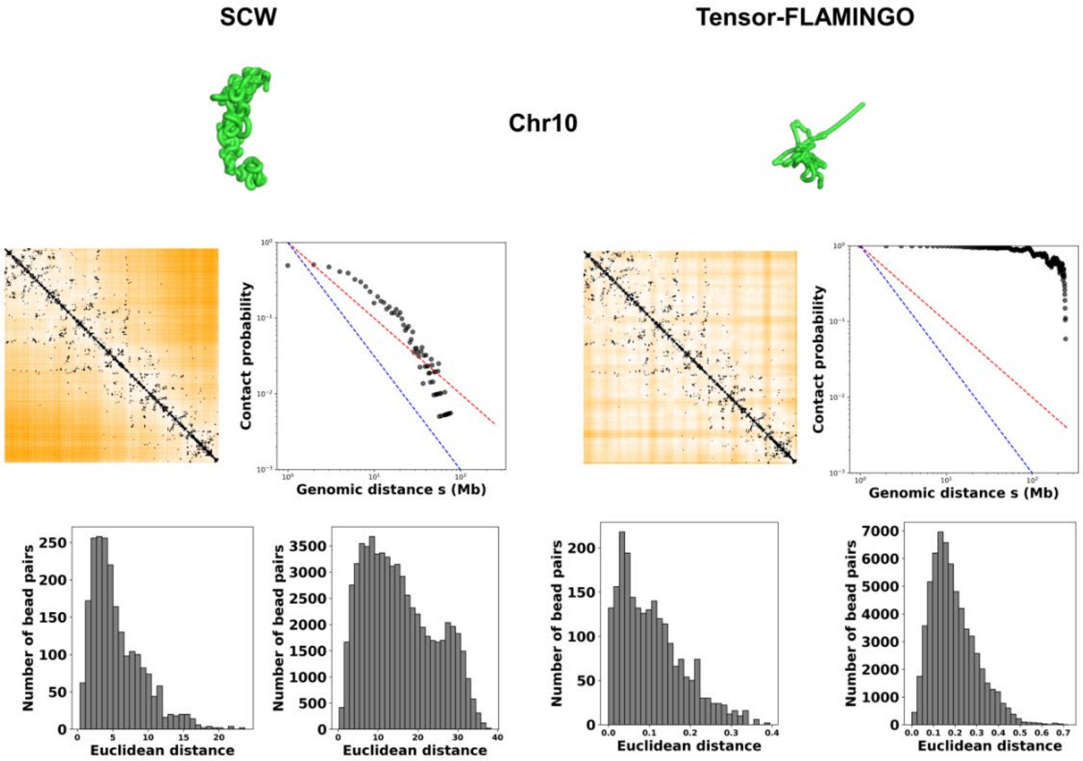

Figure S122 Intra-chromosomal structures and evaluations for TH1 Cell1 Chr10 by using SCW and Tensor-FLAMINGO

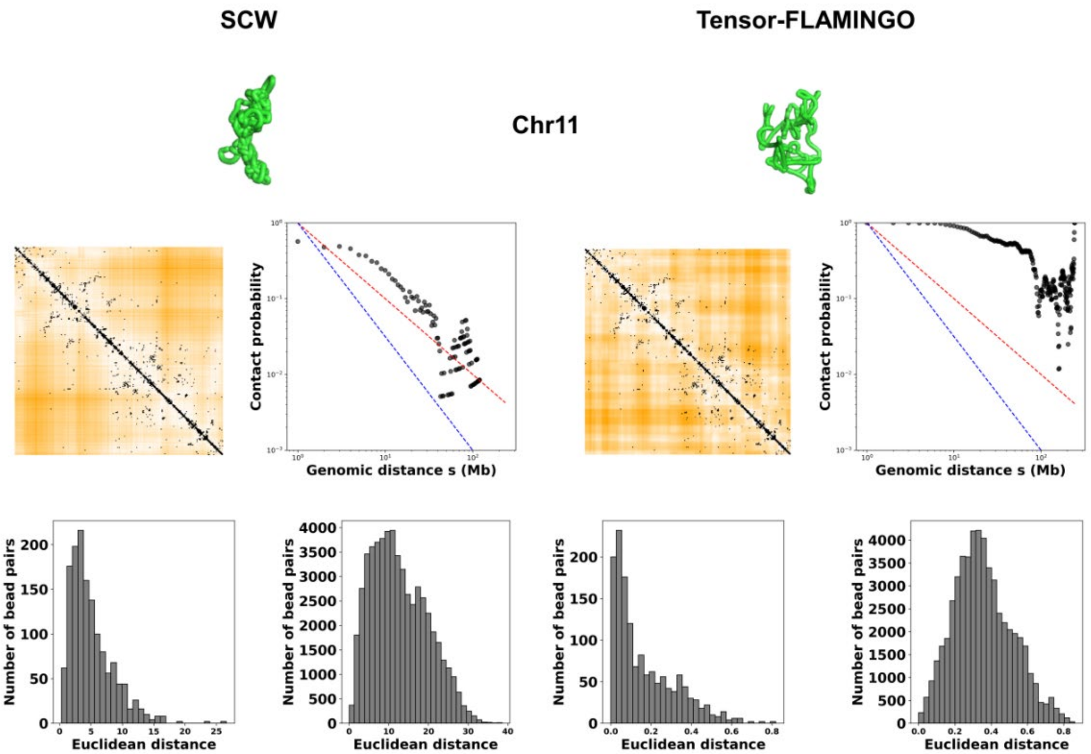

Figure S123 Intra-chromosomal structures and evaluations for TH1 Cell1 Chr11 by using SCW and Tensor-FLAMINGO

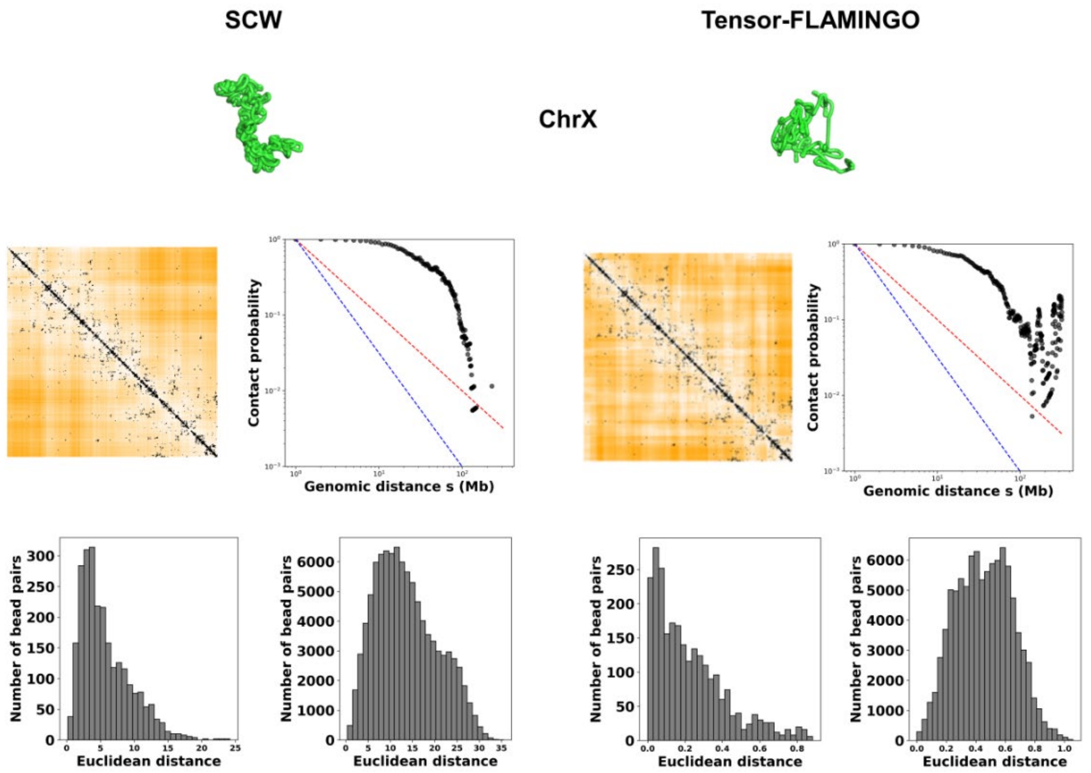

Figure S124 Intra-chromosomal structures and evaluations for TH1 Cell1 ChrX by using SCW and Tensor-FLAMINGO

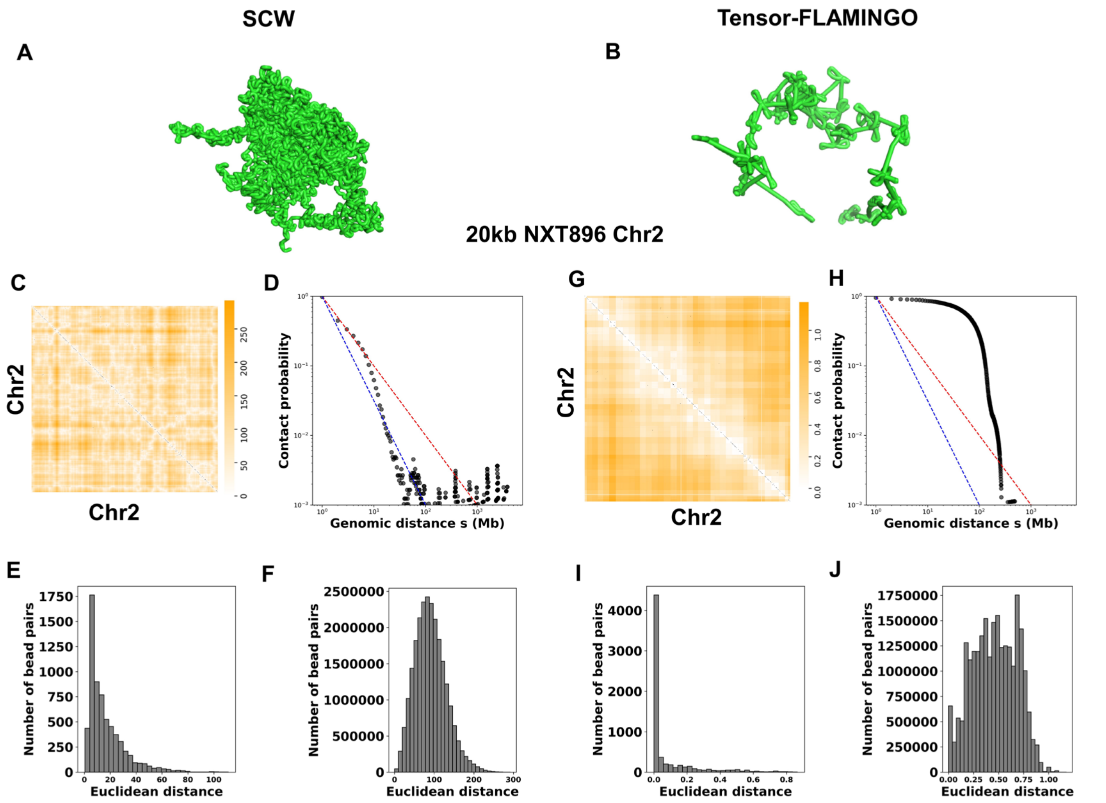

Figure S125 Intra-chromosomal structures and evaluations for NXT896 chromosome 2 at 20 Kbp resolution by using SCW and Tensor-FLAMINGO

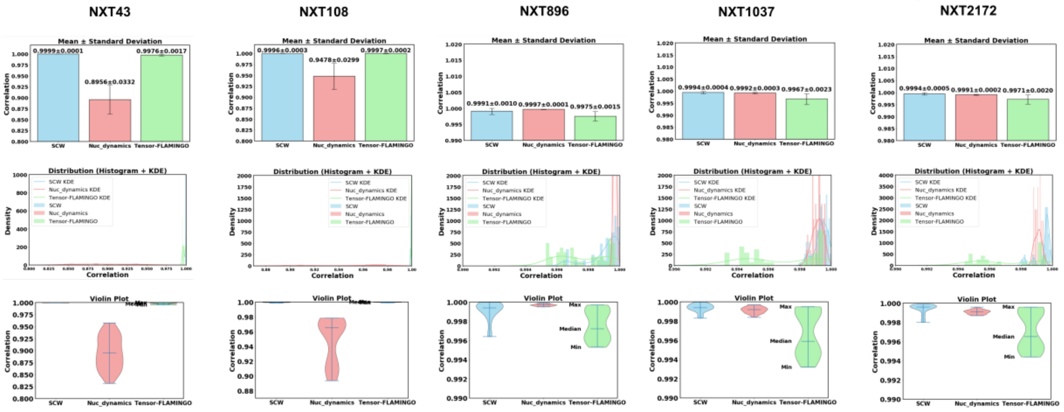

Figure S126 3D coordinates correlation across 20 structural models for 5 NXT cells at 500 Kbp resolution, structures generated by SCW, Nuc_dynamics, and Tensor-FLAMINGO.

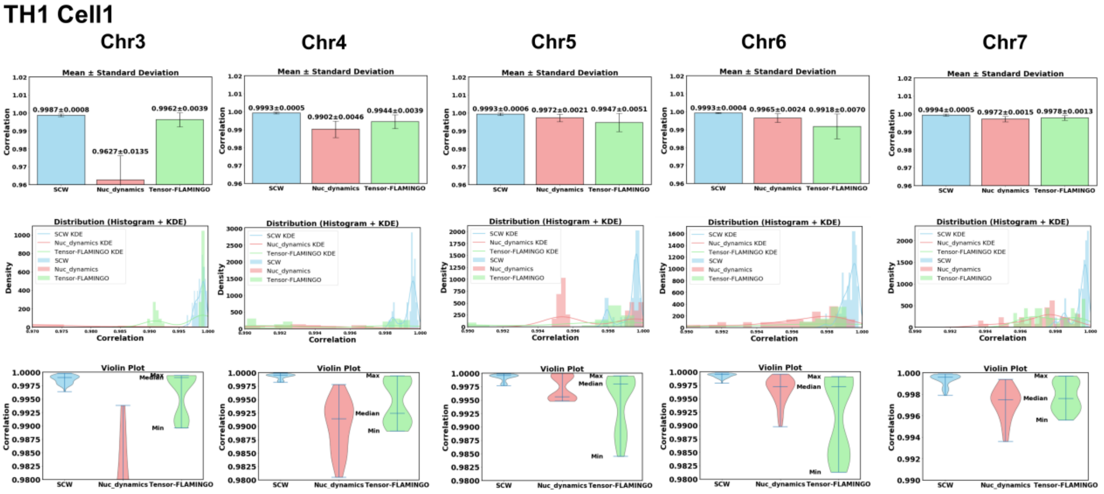

Figure S127 3D coordinates correlation across 20 structural models for 5 chromosomes of TH1 cell1 at 500 Kbp resolution, structures generated by SCW, Nuc_dynamics, and Tensor-FLAMINGO.

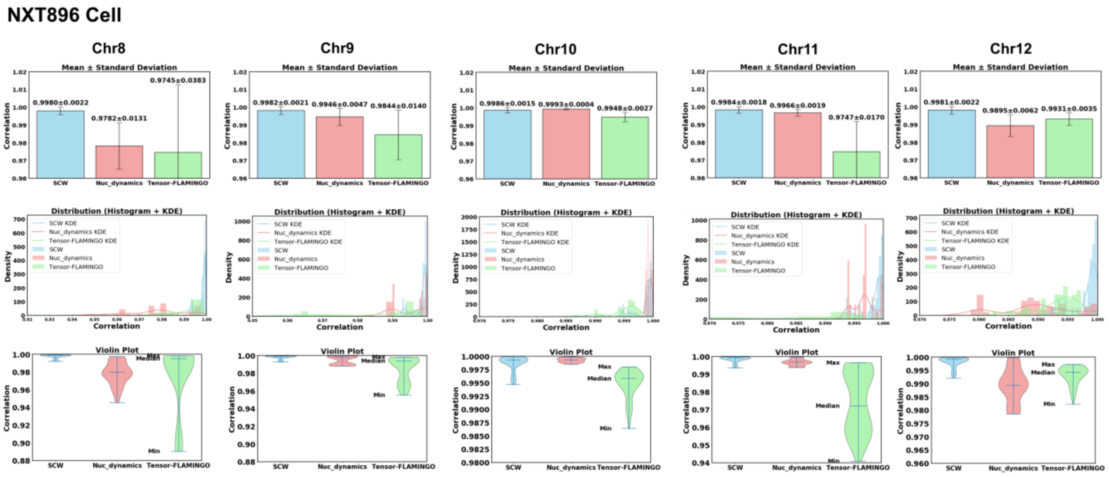

Figure S128 3D coordinates correlation across 20 structural models for 5 chromosomes of NXT896 cell at 500 Kbp resolution, structures generated by SCW, Nuc_dynamics, and Tensor-FLAMINGO.

Reference

1. Tan L, Xing D, Chang CH, Li H, Xie XS. Three-dimensional genome structures of single diploid human cells. Science. 2018;361(6405):924-8. Epub 20180830. doi: 10.1126/science.aat5641. PubMed PMID: 30166492; PubMed Central PMCID: PMCPMC6360088.
